# GALNT14-mediated *O*-glycosylation on PHB2 serine-161 enhances cell growth, migration and drug resistance by activating IGF1R cascade in hepatoma cells

**DOI:** 10.1038/s41419-022-05419-y

**Published:** 2022-11-14

**Authors:** Yu-De Chu, Tan-Chi Fan, Ming-Wei Lai, Chau-Ting Yeh

**Affiliations:** 1 Liver Research Center, Chang Gung Memorial Hospital, Taoyuan, Taiwan; 2 Institute of Stem Cell and Translational Cancer Research, Chang Gung Memorial Hospital at Linkou, Taoyuan, Taiwan; 3 Division of Pediatric Gastroenterology Department of Pediatrics, Linkou Chang Gung Memorial Hospital, Taoyuan, Taiwan; 4 Molecular Medicine Research Center, College of Medicine, Chang Gung University, Taoyuan, Taiwan

**Keywords:** Liver cancer, Insulin signalling

## Abstract

The single nucleotide polymorphism (SNP) rs9679162 located on *GALNT14* gene predicts therapeutic outcomes in patients with intermediate and advanced hepatocellular carcinoma (HCC), but the molecular mechanism remains unclear. Here, the associations between SNP genotypes, GALNT14 expression, and downstream molecular events were determined. A higher GALNT14 cancerous/noncancerous ratio was associated with the rs9679162-GG genotype, leading to an unfavorable postoperative prognosis. A novel exon-6-skipped *GALNT14* mRNA variant was identified in patients carrying the rs9679162-TT genotype, which was associated with lower GALNT14 expression and favorable prognosis. Cell-based experiments showed that elevated levels of GALNT14 promoted HCC growth, migration, and resistance to anticancer drugs. Using a comparative lectin-capture glycoproteomic approach, PHB2 was identified as a substrate for GALNT14-mediated *O*-glycosylation. Site-directed mutagenesis experiments revealed that serine-161 (Ser161) was the *O*-glycosylation site. Further analysis showed that *O*-glycosylation of PHB2-Ser161 was required for the GALNT14-mediated growth-promoting phenotype. *O*-glycosylation of PHB2 was positively correlated with GALNT14 expression in HCC, resulting in increased interaction between PHB2 and IGFBP6, which in turn led to the activation of IGF1R-mediated signaling. In conclusion, the *GALNT14*-rs9679162 genotype was associated with differential expression levels of *GALNT14* and the generation of a novel exon-6-skipped *GALNT14* mRNA variant, which was associated with a favorable prognosis in HCC. The GALNT14/PHB2/IGF1R cascade modulated the growth, migration, and anticancer drug resistance of HCC cells, thereby opening the possibility of identifying new therapeutic targets against HCC.

## Introduction

Hepatocellular carcinoma (HCC) is a leading cause of death in Southeast Asia [1, 2]. Major risk factors for HCC include chronic hepatitis B or C virus infection (HBV and HCV, respectively) and alcoholic liver disease [3]. Of all the therapeutic modalities for HCC, surgical removal of the liver tumor remains the most effective [2]. However, only a subset of HCC patients with HCC, who have an acceptable liver function and are diagnosed in an early stage, are eligible for surgical resection or liver transplantation [4]. In patients with unresectable HCC, the mainstay alternative therapies include transarterial chemoembolization (TACE) for intermediate-stage HCC and systemic therapies for advanced HCC. The latter include targeted drugs (sorafenib or lenvatinib) and immunotherapy [5]. Despite the availability of these treatments, a wide range of therapeutic responses has been observed, leading to the need for biomarkers to predict the efficacy of these therapies.

Using genome-wide association studies followed by prospective validation, germline-derived genetic variants of the polypeptide N-acetylgalactosaminyltransferase 14 (*GALNT14*) gene have been identified as predictors of response in patients with HCC receiving chemotherapy [6]. Among them, the leading single nucleotide polymorphism (SNP) rs9679162 located in intron-5 of *GALNT14* was found to be associated with therapeutic response to chemotherapy in patients with Barcelona Clinic Liver Cancer (BCLC) stage C, including time-to-tumor progression and overall survival (OS) [7, 8]. The rs9679162 genotype has also been associated with therapeutic response in patients with BCLC stage B HCC treated with TACE [9]. In these studies, the rs9679162-TT genotype was associated with favorable outcomes, whereas rs9679162-GG was associated with unfavorable outcomes. Despite these findings, the association between the rs9679162 genotype and clinical outcomes in patients with early-stage HCC treated with surgical resection has not been studied, and the underlying mechanisms leading to effective outcome prediction remain unclear.

The gene containing rs9679162 encodes the enzyme GALNT14, which belongs to the N-acetyl-galactosaminyl transferase (GalNAc-T) family. Altered expression of GALNT14 has been reported to enhance tumorigenesis and progression of breast cancer and ovarian cancer through regulation of cell proliferation, migration, invasion, and susceptibility to anticancer drugs [10–12]. As a member of the GalNAc-T family, GALNT14 is believed to achieve these goals through modulation of its transferase activity. Certain putative substrates of GALNT14 in other (not HCC) cancer cells have been proposed, including MUC13 [13], EFEMP2 [14], and DR5 [15]. However, the functional role of GALNT14 and the molecular mechanisms responsible for its prognostic role in HCC remain unclear.

Here, we investigated the correlation between the rs9679162 genotype and postoperative prognosis in patients with HCC. Additionally, differential lectin-capture analysis and cell-based experiments were conducted to identify GALNT14 enzyme substrates and understand their functional roles in HCC.

## Results

### The rs9679162 genotype was associated with postoperative prognosis in patients with HCC

**Fig. 1:**
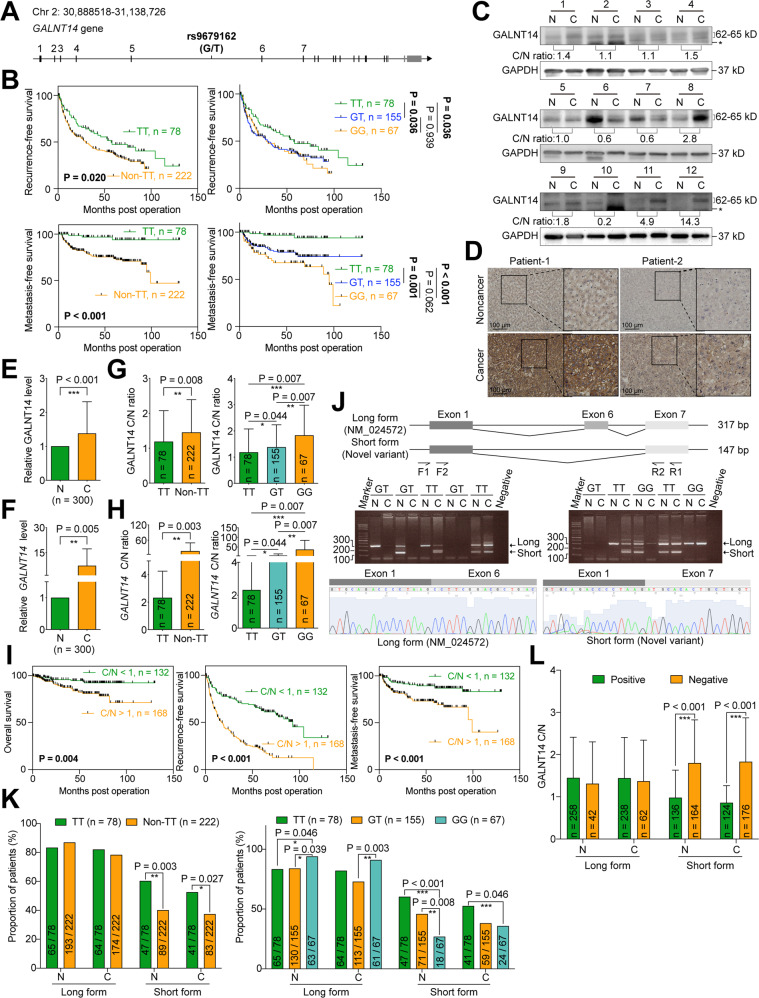
Higher cancerous/noncancerous ratios for GALNT14 and the rs9679162-non-TT genotype are associated with unfavorable postoperative outcomes in patients with HCC. **A** Genomic structure of the *GALNT14* locus. Black vertical lines indicate predicted translatable regions, while gray lines indicate non-coding regions. **B** Kaplan–Meier analysis of postoperative outcomes in patients with HCC stratified according to rs9679162 genotypes. *P* values were obtained by log-rank test. **C** Representative images of western blots of patient samples. N noncancerous part, C cancerous part. The anti-GALNT14 reactive bands migrating between 62-65 kD were verified to be *GALNT14*-derived protein species by mass spectrometry. However, the band migrating below 62 kD, marked by “*”, was not GALNT14-related. **D** Representative IHC images obtained from patients with HCC are shown. Statistical comparison of GALNT14 protein (**E**) and mRNA (**F**) levels between noncancerous (N) and cancerous (**C**) tissues. *P* values were calculated by paired two-tailed student’s *t* test. Cancerous/noncancerous (C/N) ratios of GALNT14 in protein (**G**) and mRNA (**H**) levels were compared between rs9679162-TT and non-TT (GT or GG) genotypes. *P* values were calculated by the two-tailed Mann–Whitney *U* test. **I** Kaplan–Meier analysis of overall (left), recurrence-free (middle), and metastasis-free (right) survival, stratified by the cancerous/noncancerous (C/N) ratio of GALNT14. *P* values were calculated by log-rank test. **J** Schematic representation of alternative splicing occurring on designated exons, with exon-6 deleted in the short form *GALNT14* mRNA (a novel variant). Agarose gel images show the presence of long (NM_024572, with product size 317 bp) and short (product size 147 bp) forms of *GALNT14* mRNA in HCC tissues. **K** Comparison of the proportion of patients carrying the long and short forms of *GALNT14* mRNA between the TT and non-TT (GG or GT) genotypes. Comparisons were made within the noncancerous (N) and cancerous (C) groups, respectively. *P* values were calculated by Fisher’s exact test. **L** Comparison of GALNT14 cancerous/noncancerous (C/N) ratios between patients positive and negative for the long or short form of *GALNT14* mRNAs. Again, comparisons were made between the noncancerous (N) and cancerous (C) groups. *P* values were calculated by the two-tailed Mann–Whitney *U* test.

Rs9679162 is located on intron-5 of *GALNT14* gene, between exon-5 and exon-6 (Fig. 1A). To address whether the SNP genotype was associated with postoperative prognosis, 300 patients with HCC treated with surgical resection were retrospectively enrolled. The baseline characteristics are listed in Table S1. After genotyping, the rs9679162-TT genotype was found to be associated with microvascular invasion (rs9679162-TT vs. non-TT, 19.2% vs. 35.1%, *P* = 0.014), but not with other factors. Univariate Cox regression analysis showed that the rs9679162-TT genotype was associated with favorable recurrence-free (*P* = 0.021) and metastasis-free (*P* = 0.001) survivals (RFS and MFS, respectively), but not with OS (*P* = 0.149) (Tables S2–4). Notably, multivariate analysis showed that SNP genotype was an independent predictor for MFS (Table S4, *P* = 0.002 in multivariate analysis). Kaplan–Meier analysis confirmed these findings (Figs. 1B, S1).

### GALNT14 expression was frequently upregulated in HCC with the rs9679162-GG genotype, which was associated with unfavorable postoperative prognosis

Since rs9679162 is located in intron-5 of *GALNT14* gene, we hypothesized that SNP genotype affects *GALNT14* expression levels. Accordingly, western blot (WB) and immunohistochemistry (IHC) analyses were conducted. As shown in Fig. 1C, D, GALNT14 levels were significantly increased in HCC. Quantitative results of GALNT14 protein levels in WB are shown in Fig. 1E. Real-time quantitative PCR (RT-qPCR) results confirmed that *GALNT14* transcript levels were also increased in HCC (Fig. 1F). Comparison of the cancerous/noncancerous (C/N) ratios of proteins or transcripts between groups of patients with distinct SNP genotypes demonstrated that patients with the rs9679162-non-TT genotype, particularly those with the rs9679162-GG genotype, had higher GALNT14 protein and mRNA levels (Fig. 1G, H).

Cox proportional hazards analysis showed that high levels of GALNT14 in cancerous tissues (C/N > 1) were associated with unfavorable OS, RFS, and MFS. Notably, GALNT14 C/N was an independent predictor of RFS (*P* = 0.031 in multivariate analysis) (Tables S2–4). Kaplan–Meier analysis was also consistent with this notion (Fig. 1I).

### A novel alternatively spliced *GALNT14* mRNA variant was identified in patients carrying the rs9679162-TT genotype with a favorable prognosis

To understand whether the rs9679162 genotype was associated with alternative splicing, and therefore affected *GALNT14* mRNA levels, a nested PCR assay was designed accordingly. As shown in Fig. 1J, two alternatively spliced *GALNT14* transcripts were found, tentatively called long and short forms. After sequencing, the long form was found to be identical to the mRNA sequence of *GALNT14* registered in GenBank, NM_024572 (spliced from exon-1-to-6), while the “short form” was generated by alternative splicing from exon-1-to-7. Surprisingly, this short variant sequence has not yet been reported. A comparison of the long and short forms present in the noncancerous or cancerous tissues of HCC patients with distinct genotypes revealed that the short variant was preferentially found in patients carrying the rs9679162-TT genotype, both in the noncancerous and cancerous tissues (Fig. 1K).

To investigate whether the presence of long or short forms of *GALNT14* transcripts correlated with GALNT14 expression in HCC, the C/N ratios of GALNT14 were compared between subgroups of patients with and without long or short transcripts in the noncancerous/cancerous parts. As shown in Fig. 1L, there were no significant differences in GALNT14 C/N ratios between patients with and without long variants in noncancerous or cancerous tissues. However, those with a short *GALNT14* mRNA variant had significantly reduced GALNT14 C/N ratios in noncancerous or cancerous tissues, suggesting that the expression of the short variant might reduce GALNT14 levels in HCC.

Finally, Cox proportional hazards and Kaplan–Meier analyses were performed. As shown in Tables S2–4, the presence of a short but not a long form of *GALNT14* mRNA was associated with favorable OS, RFS, and MFS. Notably, its expression in cancerous tissues was an independent predictor of OS (*P* = 0.037) and RFS (*P* < 0.001). Kaplan–Meier analysis also confirmed these findings (Fig. S2A–C).

### Altered GALNT14 expression perturbed HCC cell growth, migration, and susceptibility to anticancer drugs

**Fig. 2:**
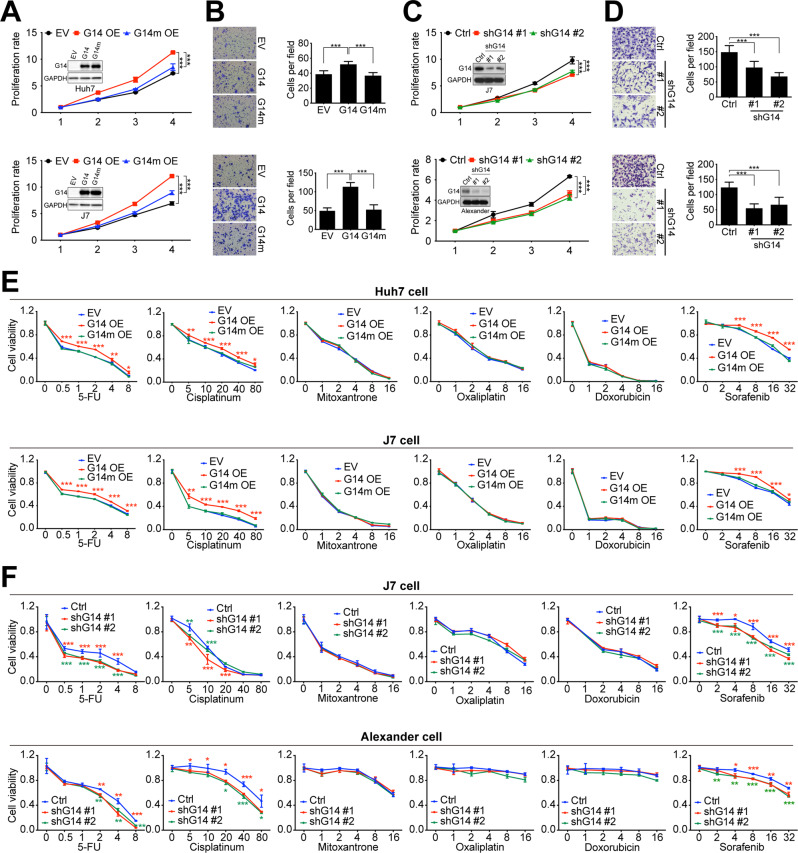
Altered expression of *GALNT14* affects the growth, migration, and sensitivity of HCC cells to anticancer drugs. **A** Cell proliferation rate of mock cells (EV), cells overexpressing GALNT14 (G14 OE), and cells overexpressing the GALNT14 mutant (G14m OE). The latter carried three enzyme-inactivating mutations. *P* values were calculated by two-way ANOVA. Western blot images are inserted to demonstrate overexpression of GALNT14 or GALNT14 mutant. Horizontal axis, days after cell seeding; vertical axis, fold increase normalized to day 1 value. **B** Representative images of transwell assays for HCC cells, Huh7 (upper) and J7 (lower), with or without overexpression of GALNT14 (or GALNT14 mutant). Quantitative results are displayed in the right panel. *P* values were calculated by paired two-tailed student’s *t* test. **C** Cell proliferation rate of HCC cells with or without GALNT14 silencing. *P* values were calculated by two-way ANOVA. Western blot images are shown indicating successful silencing of GALNT14. These blots were overexposed to demonstrate endogenous GALNT14. **D** Representative images of transwell assays for the HCC cells, Huh7 (upper) and J7 (lower), with or without GALNT14 silencing. Quantitative results are displayed in the right panel. *P* values were calculated by paired two-tailed student’s *t* test. G14 GALNT14, Ctrl mock control for shRNA (shLacZ). **E**, **F** Relative cell viability of HCC cells treated with anticancer drugs. **E** Evaluation of the sensitivity of cells overexpressing GALNT14 (G14 OE) or GALNT14 mutant (G14m OE) to anticancer drugs 5-FU (in mg/mL as marked in the horizontal axis), Cisplatin (in μg/mL), Mitoxantrone (in μg/mL), Oxaliplatin (in μg/mL), Doxorubicin (in mg/mL), and Sorafenib (in μM). **F** A similar assessment was performed on cells with GALNT14 silencing. Vertical axis, relative fold change normalized by the value of concentration “0”. *P* values at each concentration were obtained by comparing cells transfected with empty vector (EV) or control shRNA (Ctrl), and cells transfected with G14/G14m or shG14 #1/2. Unpaired two-tailed student’s *t* test was used. *P < 0.05; **P < 0.01; ***P < 0.001.

To understand the role of GALNT14 in HCC, several cell-based assays have been performed. Huh7, Alexander, and J7 cells were used for overexpression or knockdown experiments depending on their endogenous levels of GALNT14 (Fig. S3). As shown in Fig. 2A, B, GALNT14 promoted cell renewal and migration in HCC cells in a GalNAc-T activity-dependent manner. Notably, the tumorigenicity of xenografts also increased with GALNT14 overexpression (Fig. S4). Conversely, silencing GALNT14 suppressed cell proliferation and migration (Fig. 2C, D).

The rs9679162 genotype was initially found to be associated with response to cytotoxic chemotherapy, such as the combination regimens of 5-FU, cisplatin, and mitoxantrone [6–8], implying that GALNT14 might be involved in the modulation of cell susceptibility to anticancer drugs. Accordingly, cells overexpressing or knocked down for GALNT14 were assessed for their susceptibility to anticancer drugs, including 5-FU, cisplatin, mitoxantrone, oxaliplatin, doxorubicin, and sorafenib. As shown in Fig. 2E, cells overexpressing GALNT14 showed reduced sensitivity to 5-FU, cisplatin, and sorafenib in a GalNAc-T activity-dependent manner, but not to mitoxantrone, oxaliplatin and doxorubicin. Consistently, silencing GALNT14 enhanced cell sensitivity to sorafenib, 5-FU and cisplatin, but not to mitoxantrone, oxaliplatin and doxorubicin (Fig. 2F).

### PHB2 as a candidate substrate for GALNT14-mediated *O*-glycosylation in HCC

**Fig. 3:**
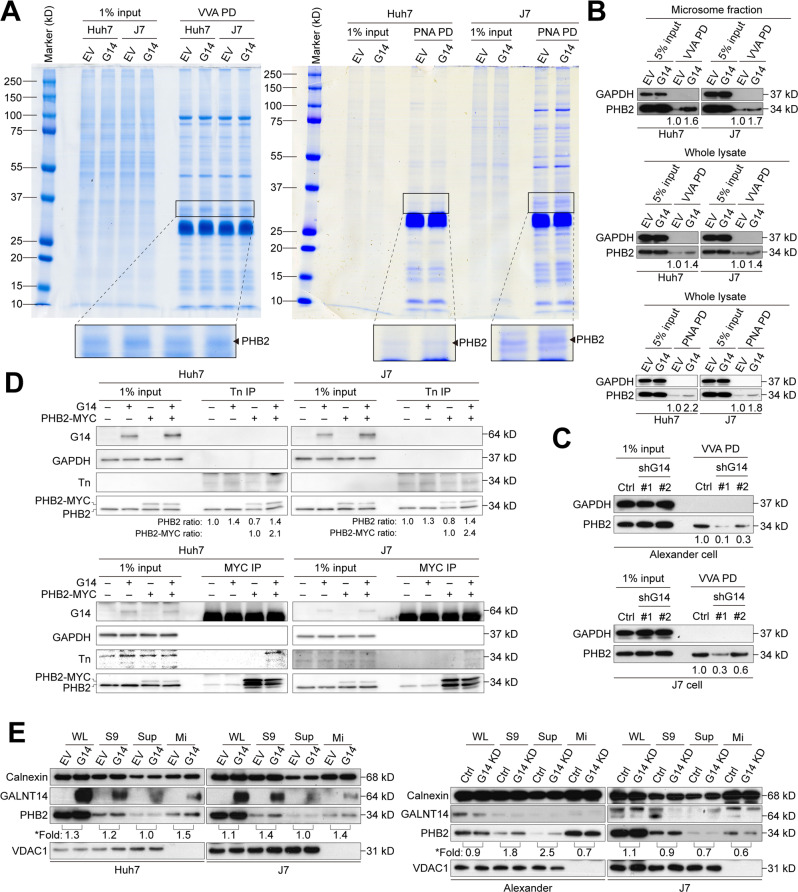
PHB2 is a promising GALNT14 substrate. **A** Microsomal fractions isolated from Huh7 and J7 cells were subjected to Vicia villosa (VVA)- or peanut agglutinin (PNA)-mediated pulldown (PD). Pulldown proteins were separated by 10% SDS-PAGE and stained by the colloidal blue silver stain. Protein bands with significant density changes were excised for LC/MS/MS identification. **B**, **C** Representative western blots demonstrating enriched PHB2 levels by VVA- or PNA-mediated pulldown in HCC cells with or without GALNT14 overexpression or silencing. **D** Western blotting following Tn antigen or MYC-tag immunoprecipitation (IP) using lysates of HCC cells transfected with the indicated plasmids. **E** Distribution of PHB2 and GALNT14 in microsomal fractions assessed by western blot using lysates of Huh7 and J7 cells with GALNT14 overexpression (upper) and silencing (lower). Calnexin was used as a specific marker for microsome fraction, while VDAC1 was used as a marker for the mitochondrial fraction. *Fold, fold of increase upon GALN14 (G14) overexpression.

To investigate whether the substrates of GALNT14-mediated *O*-glycosylation played a crucial role in modulating the aforementioned cell phenotypes, a lectin-mediated pulldown assay was performed. Consistent with recent reports [16, 17], we found that GALNT14 could translocate from the Golgi apparatus to the lumen of the endoplasmic reticulum (ER) in HCC (Fig. S5). Hence, the ER-containing microsomal fraction derived from GALNT14-overexpressing cells was used in a lectin-mediated pulldown assay (Fig. S6). Visible enrichment of protein bands was observed after gel electrophoresis and colloidal blue silver staining following Vicia villosa lectin (VVA)- or peanut agglutinin (PNA)-pulldown. Identification by LC-MS/MS after trypsinization in the gel indicated that it was most likely PHB2 (Fig. 3A).

Subsequent WB validation confirmed that PHB2 was a candidate substrate for GALNT14-mediated *O*-glycosylation. The experiments were conducted using lysates from the microsomal fraction or whole extract of GALNT14-overexpressing or silenced HCC cells (Figs. 3B, C, S7). Similar results were observed in the experiments using PNA-lectin (Fig. 3B, C). To understand whether PHB2 is indeed a substrate for GALNT14-mediated *O*-glycosylation, GalNac (Tn antigen) present in PHB2 was examined. As shown in Fig. 3D, PHB2 was enriched in Tn immunoprecipitates when GALNT14-overexpressing cell lysates were used (upper panels). Reciprocally, the Tn signal was observed only in immunoprecipitates of exo-PHB2 (with MYC tagged) from cell lysates co-expressing GALNT14 (lower panel).

Finally, subcellular fractionation experiments showed that the abundance of PHB2 in the microsomal fraction increased in a GALNT14 level-dependent manner (Fig. 3E).

### PHB2 Ser161 was a GALNT14-mediated *O*-glycosylation site that enhanced PHB2 membrane localization

**Fig. 4:**
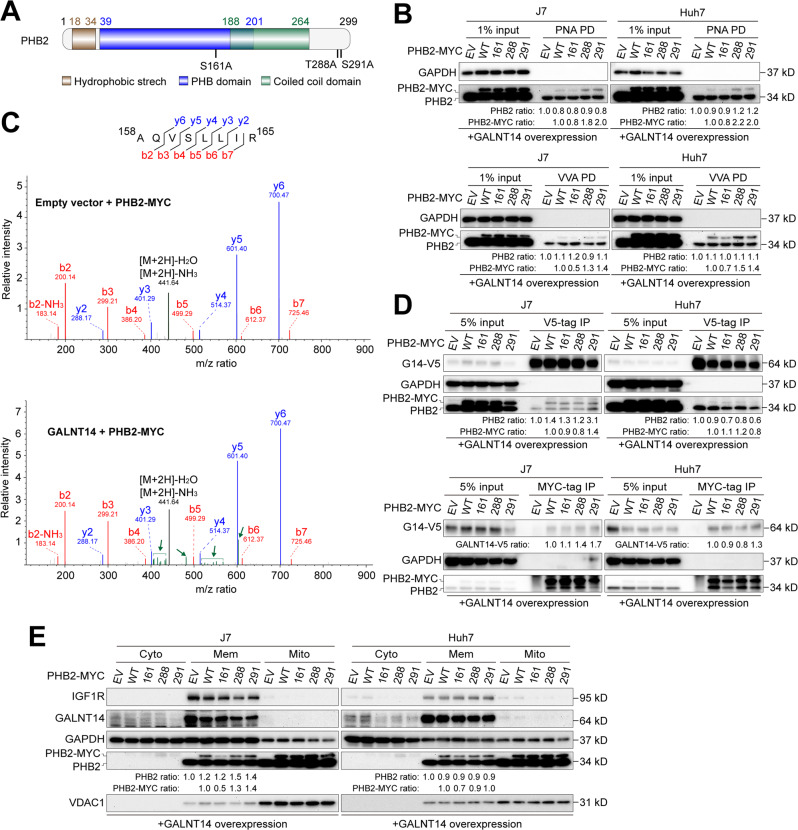
Serine residue 161 of PHB2 serves as a target site for GALNT14-mediated *O*-glycosylation. **A** Schematic representation of predicted PHB2 domains and residues as *O*-glycosylation hotspots. **B** PNA- or VVA-mediated pulldown of PHB2 was performed using lysates of cells transfected with the indicated plasmids. **C** Spectrum of Serine-161-containing peptides derived from LC/MS/MS. Green bars and arrows indicate that there were certain uncharacterized modifications on the fragmented peptides. m/z, the mass-to-charge ratio, where m is the molecular or atomic mass number and z is the charge number of the ion. **D** Western blot analysis following co-immunoprecipitation using samples of HCC cells transfected with the indicated plasmids. **E** Western blot analysis of subcellular fractions fractionated from cells transfected with the indicated plasmids, including cytosolic (Cyto), membranous (Mem) and mitochondrial (Mito). IGF1R was used as a specific marker for the membrane fraction, while VDAC1 was used as a marker for the mitochondrial fraction.

To identify specific *O*-glycosyl residues in PHB2, possible sites were estimated using a previously reported method [18]. Three residues, Ser161, Thr288, and Ser291, were predicted as potential *O*-glycosylation sites (Fig. 4A). Site-directed mutagenesis was preformed to replace serine/threonine residues with alanine. In HCC cells overexpressing GALNT14, only PHB2 with the Ser161Ala point mutation attenuated its ability to bind either VVA or PNA, but not the Thr288Ala or Ser291Ala substitutions (Fig. 4B). To further examine whether GALNT14-mediated *O*-glycosylation indeed occurred on PHB2 at Ser161, LC-MS/MS was performed using anti-MYC immunoprecipitated samples with or without GALNT14 overexpression (Fig. S8). The mass-to-charge (m/z) ratio of Ser161-containing peptides after trypsinization in the gel was analyzed. As shown in Fig. 4C, an uncharacterized set of peptides presented after peptide-b4, -b5, and -y5 was observed in GALNT14-overexpressing cell samples (green color), suggesting an increased mass of Ser161-containing peptides, likely due to GALNT14-mediated *O*-glycosylation.

As an effective substrate, PHB2 can be assumed to physically interact with GALNT14. As shown in Fig. S9A, B, exo-PHB2 was able to bind with GALNT14. This association persisted, regardless of the presence of Ser161Ala, Thr288Ala, or Ser291Ala mutations (Fig. 4D), indicating that point mutations did not influence the association between GALNT14 and PHB2 in HCC cells.

As reported previously [19], PHB2 is a membrane protein. To understand whether its membrane localization is regulated by the expression of GALNT14, the subcellular distribution of the proteins was examined. As shown in Fig. S9C, D, overexpression of GALNT14 increased the levels of both intrinsic and extraneous PHB2 in the membrane protein fraction. Intriguingly, the Ser161 mutation attenuated GALNT14-mediated membrane localization of PHB2 in HCC cells (Fig. 4E). Immunofluorescence assays also confirmed that the Ser161Ala mutation affected the subcellular localization of exo-PHB2 in HCC cells (Fig. S10).

### PHB2-Ser161 *O*-glycosylation was required for GALNT14-modulated growth promotion in HCC cells

To understand whether PHB2 is involved in the GALNT14-mediated growth-promoting phenotypes, siRNA-induced silencing of PHB2 was performed under GALNT14 overexpression. Three distinct siRNAs were used: exon-targeting siPHB2-#1 and siPHB2-#2, and 3’-untranslated region (UTR)-targeting siPHB2-#3. As shown in Fig. S11A, all of them efficiently silenced endo-PHB2. However, cells expressing siPHB2-#3 were unable to suppress exo-PHB2 because it lacked the 3’UTR of the mRNA for targeting. Silencing of PHB2 with siPHB2-#1 or siPHB2-#2 suppressed cell growth and migration, which could be attenuated by the co-expression of siPHB2-#3 and exo-PHB2 (Fig. S11B–D). Similarly, the knockdown of PHB2 by siPHB2-#1 or siPHB2-#2 attenuated GALNT14-mediated reduction in anticancer drug sensitivity in HCC cells, whereas it was restored by co-expression of siPHB2-#3 and exo-PHB2 (Fig. S11E, F). These results imply that PHB2 could be a downstream effector of GALNT14.

**Fig. 5:**
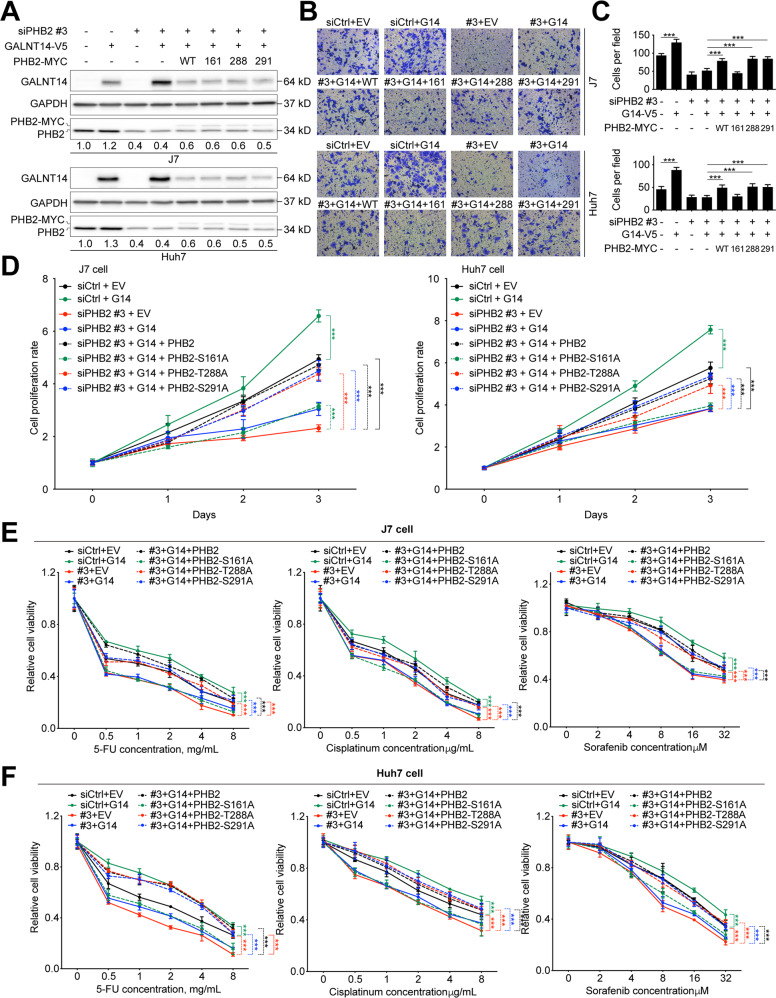
*O*-glycosylation of PHB2 at Ser161 is crucial to orchestrate the GALNT14-induced phenotype. **A** Western blot analysis of lysates of cells transfected with the indicated plasmids. **B** Representative images of transwell-based migration assays. Quantitative results of migrated cells are shown in (**C**). *P* values were calculated by the two-tailed Mann–Whitney *U* test. **D** Alarmar blue-based cell growth assay using cells transfected with the indicated plasmids. *P* values were calculated by the two-way ANOVA. **E**, **F** Relative cell viability of anticancer drug-treated HCC cells transfected with the indicated plasmids. Three anticancer drugs were tested, including 5-FU, Cisplatin, and Sorafenib. *P* values were calculated by the two-way ANOVA. ***P < 0.001.

To understand whether GALNT14-mediated *O*-glycosylation of PHB2 plays a role in HCC cell growth regulation, the growth-regulating effects of the wild-type and three PHB2 mutants were compared. In these experiments, co-expression of siPHB2-#3, exo-PHB2, and GALNT14 was achieved (Fig. 5A). Notably, GALNT14 levels decreased in the triple expression experiments, and a slight increase in PHB2 expression was observed when GALNT14 was overexpressed (Fig. 5A). As shown in Fig. 5B–D, silencing of endo-PHB2 reduced GALNT14-mediated migration and growth promotion. However, co-expression of wild-type exo-PHB2 restored the GALNT14-mediated migration and growth-promoting effects. This exo-PHB2-mediated restoration was achieved by the co-expression of exo-PHB2 carrying the wild-type sequence or exo-PHB2 carrying the Thr288Ala or Ser291Ala, but not the Ser161Ala substitution. Similarly, the reduced sensitivity of HCC cells to anticancer drugs caused by GALN14 was reversed by endo-PHB2 silencing (Fig. 5E, F). This effect was restored by supplying exo-PHB2 carrying the wild-type sequence or exo-PHB2 carrying Thr288Ala or Ser291Ala, but not the Ser161Ala substitution.

### PHB2 was upregulated in HCC and its *O*-glycosylation status correlated with GALNT14 expression

**Fig. 6:**
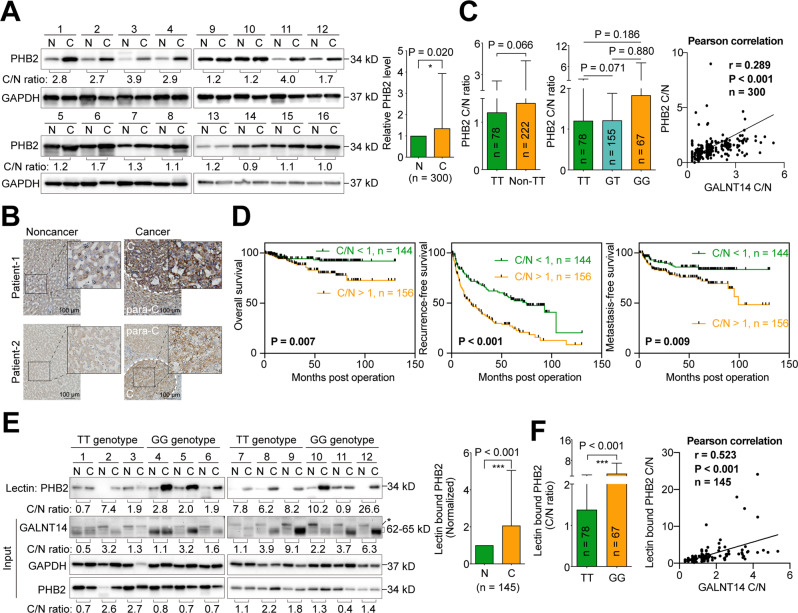
PHB2 and its *O*-glycosylated forms are upregulated in HCC. **A** Representative images of western blots of HCC patient samples. N noncancerous part, C cancerous part. Statistical comparison between PHB2 levels in the noncancerous and cancerous parts is given in the right panel. *P* values were calculated by paired two-tailed student’s *t* test. **B** Representative IHC images of HCC patient tissues. The scale bar is given in the right lower corner. C cancerous tissue, para-C para-cancerous tissue. **C** Statistical comparison of PHB2 cancerous/noncancerous (C/N) ratios between tissues from patients with the indicated rs9679162 genotypes (left and middle panel). *P* values were calculated by the two-tailed Mann–Whitney *U* test. The C/N ratios of GALNT14 protein and PHB2 protein were correlated using the Pearson correlation (right panel). **D** Kaplan–Meier analysis of overall (left), recurrence-free (middle), and metastasis-free (right) survival, stratified by the C/N ratio of PHB2 protein. *P* values were calculated by log-rank test. **E** Representative image of western blots of samples of lectin pulldown assays using lysates extracted from HCC tissues (upper panel). A statistical comparison of PHB2 protein levels between the noncancerous (N) and cancerous (C) tissues is given in the right panel. *P* value was calculated by paired two-tailed student’s *t* test. **F** Statistical comparison of the C/N ratio of lectin-bound PHB2 in HCC tissues between patients with the indicated rs9679162 genotype (left panel). The C/N ratios of GALNT14 protein and lectin-bound PHB2 protein were correlated using the Pearson correlation (right panel). TT rs9679162-TT genotype, Non-TT rs9679162-non-TT genotype, GT rs9679162-GT genotype, GG rs9679162-GG genotype.

Albeit the relationship between GALNT14 and PHB2 has been investigated in HCC cell lines, it remains unclear whether these findings can be reflected in HCC tissues. Therefore, PHB2 levels were assessed in HCC tissues. As shown in Fig. 6A, PHB2 was upregulated in the cancerous portion of HCC. The IHC staining results also supported this finding (Fig. 6B). Although PHB2 levels were not associated with the rs9679162 genotype (or were only borderline significant), they were markedly associated with GALNT14 expression (Fig. 6C), which was correlated with postoperative outcomes (Fig. 6D and Tables S2–4). These results imply that PHB2 plays an oncogenic or growth-promoting role in HCC, reflecting the cell-based findings that overexpression of PHB2 enhanced cell proliferation, migration, and resistance to anticancer drugs (Fig. S11), and overexpression of GALNT14 increased the levels of PHB2 (Fig. 5A).

To determine the *O*-glycosylation status of PHB2 in HCC, a lectin-mediated pulldown assay was performed. Tissues from patients carrying the rs9679162-TT and -GG genotypes (*n* = 78 and 67, respectively) were tested. As shown in Fig. 6E, the degree of PHB2 *O*-glycosylation was markedly increased in cancerous sections and normalized to PHB2 levels in the input. The C/N ratio of normalized lectin-bound PHB2 was significantly increased in patients carrying the rs9679162-GG genotype and was correlated with the C/N ratio of GALNT14 (Fig. 6F).

### GALNT14-mediated *O*-glycosylation of PHB2-Ser161 promoted its association with IGFBP6 and activated IGF1R signaling in HCC cells

**Fig. 7:**
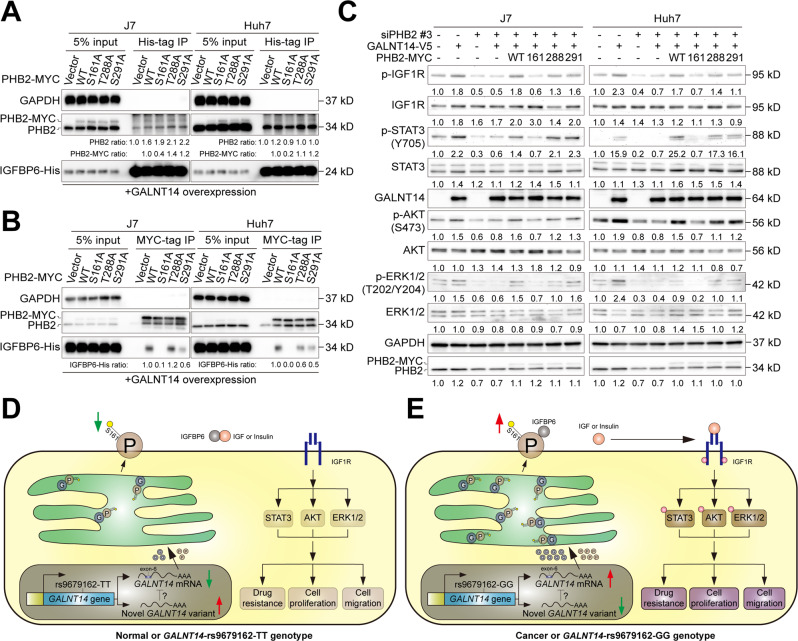
GALNT14-mediated *O*-glycosylation of PHB2 at Ser161 promotes IGF1R-mediated signaling through the association of PHB2 with IGFBP6. **A**, **B** Western blot analysis of co-immunoprecipitation assays using HCC cell samples treated as indicated. **C** Western blot analysis of HCC cell lysates treated as indicated. **D**, **E** Working model of this study. The newly discovered short RNA variant of *GALNT14* is present and able to attenuate the expression of exon-6-containing mRNA in normal hepatocytes or in patients carrying the rs9679162-TT genotype. Consequently, lower levels of GALNT14, PHB2, and *O*-glycosylated PHB2 are produced, preventing activation of IGF1R-mediated signaling (**D**). However, in cancerous hepatocytes or patients carrying the rs9679162-GG genotype, the novel RNA variant of *GALNT14* was absent and failed to suppress the expression of mRNA containing exon-6, resulting in increased levels of GALNT14, PHB2, and *O*-glycosylated PHB2. The increase in *O*-glycosylated PHB2 competes for binding with IGFBP6, thereby increasing IGF (or insulin) release to activate the IGF1R-mediated signaling cascade.

PHB2 has been demonstrated to co-localize and physically interact with the IGF1R modulator IGFBP6 on the cell membrane [19]. To understand whether GLANT14-mediated *O*-glycosylation of Ser161 plays a role in the association between PHB2 and IGFBP6, a series of co-immunoprecipitation (co-IP) experiments were conducted. As shown in Fig. 7A, when IGFBP6-His immunoprecipitation was performed, a comparable amount of endo-PHB2 was detected in all the pellets. However, a significantly reduced abundance of exo-PHB2 was detected in the samples expressing the Ser161Ala mutant. Consistently, when PHB2-MYC was immunoprecipitated, IGFBP6 was detectable in the co-immunoprecipitants of the wild-type or Thr288Ala and Ser291Ala mutants, but not in the Ser161Ala mutant. IGFBP6 was almost absent (or present in trace amounts) in the last immunoprecipitant (Fig. 7B). These results indicate that GALNT14-mediated *O*-glycosylation of PHB2-Ser161 is essential for its association with IGFBP6 in HCC cells.

To assess whether IGF1R signaling regulated by IGFBP6 was activated following enhanced PHB2-IGFBP6 association, the activation status of several key protein players in this pathway was examined. As shown in Fig. 7C, activation of IGF1R-mediated signaling upon GALNT14 overexpression depended on *O*-glycosylation of PHB2-Ser161, indicating that GLANT14-mediated *O*-glycosylation of PHB2-Ser161 is required for activation of the IGF1R cascade.

## Discussion

Over the past decade, genetic variants of *GALNT14* and its gene product have emerged as novel prognostic or therapeutic predictors for various gastrointestinal, ovarian, breast, and lung cancers [20]. However, the underlying mechanisms of the associations between genetic variants and prognosis remain largely unknown. In HCC, a novel mRNA variant of *GALNT14* has been identified, wherein alternative splicing occurs in exon-1-to-7 (skipping the original constituent exon-6). This variant was enriched in patients with the rs9679162-TT genotype (Fig. 1K). Although the exact mRNA sequence and its biological function remain unknown, it can be speculated that such variants may lead to the translation of small peptides (or truncated proteins) or the production of non-coding RNAs (ncRNAs), since the inclusion of exon-6 is essential for an intact open reading frame of GALNT14. Since the presence of the exon-6-skipping variant was found to be significantly associated with lower levels of GALNT14 in this study (Fig. 1L), these putative small peptides or ncRNAs might inhibit GALNT14 expression in HCC through several previously reported pathways [21].

In contrast, regulation of gene expression by alternative splicing associated with genetic variants has been demonstrated in other genetic variants, where relationships between genotypic and phenotypic differences can be established [22, 23]. A compelling explanation for alternative splicing mediated by genetic variation is that putative RNA-binding proteins (RBPs) have different binding abilities to bind motifs on nascent RNAs with distinct genotypes [24]. Although differential binding can occur on RNAs with only one SNP [25], it is more convincing if there are multiple adjacent SNPs that are genetically closely related. In the case of rs9679162, linkage disequilibrium has been found in a 5-kb region centered on rs9679162, which contains at least 18 SNPs. This was assessed using a dataset of 45 unrelated Chinese subjects from the HapMap Chinese Han Beijing Phase II cohort [6]. In our recently unpublished results, we discovered that this haplotype block was not limited to the 18-SNP region, but the linkage disequilibrium extended to 39 other loci close to rs9679162, spanning a region of 36-kb in the same intron. These data suggest that the rs9679162-containing introns derived from individuals with the rs9679162-TT and -GG genotypes are genetically (and thus functionally) significantly different. Therefore, distinct alternative splicing patterns (and thus GALNT14 expression levels and clinical outcomes) can be expected.

The functional role of GALNT14 in HCC has not yet been clearly elucidated. In other cancers, GALNT14 has been shown to promote tumorigenesis or cancer progression by enhancing cell proliferation, migration, and invasion and by reducing cell sensitivity to anticancer drugs [10–12]. However, the underlying molecular mechanisms remain unclear, alhtough a group of substrates has been proposed as master regulators downstream of GALNT14 [13–15]. The mechanism revealed in the present study may not be the only pathway contributing to the different malignant phenotypes between patients carrying the rs9679162-TT and non-TT genotypes. The SNP is a germline variant, and thus, its effects may be systemic. Other alterations in the microenvironment, such as immunological differences between the different genotypes, can be speculated.

In this study, GALNT14 expression was higher in patients with HCC having the rs9679162-GG genotype (Fig. 1). A higher GALNT14 level was found to promote HCC cell growth and migration, which can explain why a higher percentage of patients with microvascular invasion was observed in patients with the rs9679162-non-TT genotype (Table S1). These results are consistent with previously reported findings regarding the roles of GALNT14 in other cancers, although the downstream substrates differed between previous and present studies [10–15]. Here, PHB2 was identified and verified as a substrate for GALNT14-mediated *O*-glycosylation in HCC (Figs. 3, 4). A previous study demonstrated that PHB2 promotes the progression of malignancy in a hypoxic microenvironment and increases the resistance of HCC cells to chemotherapy-induced apoptosis [26]. PHB2 is predominantly present in the inner membrane of mitochondria and acts as a mitophagy receptor [27]. However, it can also be distributed to other subcellular locations, including the nucleus [28], perinuclear [29], and cell membrane [19]. Consistent with previous studies, elevated levels of PHB2 were found in patient-derived HCC tissues (Fig. 6A), and PHB2 expression promoted cell growth, migration, and resistance to anticancer drugs (Fig. 5). Moreover, we found that these effects resulted from GALNT14-mediated *O*-glycosylation at Ser161 of PHB2. Interestingly, although the mechanism remains unclear, GALNT14 expression was found to slightly upregulate PHB2 levels in cell-based experiments, and the abundance of these two proteins was positively correlated in HCC tissues (Figs. 5–7), suggesting a dual mechanism of functional improvement. Notably, while abundant levels of PHB2 were localized to the mitochondria, a sustained proportion of PHB2 was distributed to the membrane (microsomal) fraction under GALNT14 overexpression (Figs. 4F, S9C, D). This evidence supports that PHB2 is a downstream effector of GALNT14 in HCC.

In conclusion, we identified a novel *GALNT14* mRNA variant generated by alternative splicing that was associated with the rs9679162-TT genotype. Patients with HCC carrying the rs9679162-TT genotype had lower GALNT14 expression and favorable postoperative clinical outcomes. Overexpression of GALNT14 enhanced hepatocarcinogenesis/progression and resistance to anticancer drugs via *O*-glycosylation at Ser161 of PHB2 in the ER and/or cell membrane, which subsequently promoted the PHB2-IGFBP6 association, thereby releasing IGF to activate IGF1R-mediated signaling pathways (Fig. 7D, E).

## Materials and methods

### Patients

For the analysis of clinical outcomes, 300 HCC patients having received surgical resection were enrolled retrospectively. Their frozen and paraffin-embedded tissues were retrieved from the Tissue Bank with the approval of the Institutional Review Board of Chang Gung Memorial Hospital, Taiwan (201900261B0C102). Clinical parameters, including gender, age, HBV surface antigen (HBsAg), anti-HCV antibody, alcoholism, liver cirrhosis status, presence of ascites, histology grade, vascular invasion status, tumor number, largest tumor size (in diameter), alpha-fetoprotein, albumin, bilirubin, aspartate transaminase (AST), alanine transaminase (ALT), creatinine, and prothrombin time, date of hepatic recurrence, date of extrahepatic metastasis, and date of last follow-up or HCC-related death were analyzed retrospectively.

### GALNT14-rs9679162 genotyping

*GALNT14*-rs9679162 genotyping was conducted as described previously [30]. Details are available in Supplementary Information.

### Lysate preparation and western blot (WB)

Protein lysate preparation and WB were conducted as described previously [31]. Details are available in Supplementary Information.

### RNA isolation, cDNA preparation and RT-qPCR

Total RNA isolation was performed as described previously [32]. First strand cDNA synthesis was conducted using ToolScript MMLV RT kit (BIOTOOLS, New Taipei City, Taiwan, Cat: TGKRA04) according to the manufacturer’s instructions. Real-time quantitative PCR (RT-qPCR) was performed using the QuantStudio 5 Real-Time PCR system (Applied Biosystems, Waltham, MA, USA, Cat: A34322).

### IHC staining

IHC was conducted as described previously [33]. The antibodies used were listed in Supplementary Information.

### Nested PCR for detection of alternatively spliced variant

*GALNT14* mRNA variants generated by alternative splicing were assessed by using tissue-derived cDNAs, 200 ng per reaction. Primers, *GALNT14*_alternative_splicing_F1: GAAGCGGCAAAGGGGACCAT, *GALNT14*_alternative_splicing_R1: TGGAAGGTGATGATGATGCT, *GALNT14*_alternative_splicing_F2: GTCGGCTGGTTCTGCCAGTCTTCG, and *GALNT14*_alternative_splicing_R2: TGGAAGGTCCGTGCAATACACCAG, were used for nested RT-PCR analysis. All the PCR products were sequence verified.

### Cell culture and transfection

The HCC cell lines, Huh7 [Research Reseource Identification (RRID): CVCL_0336], J7 (RRID: CVCL_4Z69) and Alexander (RRID: CVCL_0485), were used in this study. They were gifts from Dr. Kwang-Huei Lin, Chang Gung University, Taoyuan, Taiwan. They were maintained in Dulbecco’s Modified Eagle Medium (DMEM) or alpha-Minimum Essential Medium (MEM) with 10% of fetal bovine serum in a humidified 37 °C incubator with 5% CO_2_. Contamination of mycoplasma was routinely examined. Transfection was performed using the Maestrofectin transfection reagent (Omics Bio, New Taipei City, Taiwan, Cat: MF002) according to the manufacturer’s instructions. Insulin-like growth factor-1 (IGF1) (SinoBiological, BDA, Beijing, China, Cat: 10598-HNAE) was supplemented to the medium at a final concentration of 50 ng/mL to improve the IGF1 Receptor (IGF1R) signaling 3-h before cells were harvested.

### Lentivirus-mediated knockdown of GALNT14

The lentivirus-mediated downregulation of *GALNT14* was performed using a previously described procedure [31]. Control shRNA against *LacZ* (TRCN0000231700), and *GALNT14* shRNA clones (TRCN0000035215 and TRCN0000035216), were purchased from RNAi Core Laboratory, Academic Sinica, Taipei, Taiwan.

### Plasmid construction

Details are available in Supplementary Information. GALNT14 transferase mutant containing triple mutations at D199A, H201A and E203A was constructed according to a previous report [10].

### Cell proliferation and viability assays

Cell growth rates were measured as previously described [34]. For cell viability assay, 1 × 10^4^ cells were seeded onto a culture disk at least 16-h before adding anticancer drugs to the medium. The ranges of drugs used were: 5-FU, 0–8 mg/mL; Cisplatin, 0–80 μg/mL; Mitoxantrone, 0–16 μg/mL; Oxaliplatin, 0–160 μg/mL; Doxorubicin, 0–160 mg/mL; Sorafenib, 0–32 μM. After 24 h of incubation, the cell viability was assessed using the Alarmar Blue assay (Invitrogen, Waltham, MA, USA, Cat: DAL1025).

### Transwell-based migration assay

Cell migration assays were conducted as previously described [33]. Briefly, 5 × 10^4^ cells were seeded in the upper chamber of a transwell filter. After 24 h of incubation, the cells were fixed with formaldehyde and stained with crystal violet.

### Microsome isolation

Microsome isolation was performed using a Microsome Isolation kit (BioVision, Waltham, MA, USA, Cat: K249) according to the manufacturer’s instructions. Details are available in Supplementary Information.

### Colloidal blue silver staining

To visualize the proteins in the gel, the colloidal staining method was employed using the Colloidal Blue Staining kit (Invitrogen, LC6025) according to the manufacturer’s instructions.

### Lectin-mediated pulldown assay

To pulldown *O*-glycosylated proteins, agarose-bound VVA (Vector Laboratories, Newark, CA, USA, Cat: AL-1233-2) and PNA (Vector Laboratories, AL-1073) were utilized. For each assay, 500 μg of lysate extracted with RIPA buffer (BIOTOOLS, TAAR-ZBZ5) was mixed with 30 μL of VVA- or PNA-conjugated agarose beads and incubated at 4 °C with constant rotation for 16 h. Subsequently, the beads were washed 5-times with 1 mL of pre-chilled RIPA buffer and then used for WB analysis.

### Liquid chromatography-mass spectrometry/mass spectrometry (LC-MS/MS) analysis

Peptides were extracted from excised gels. After trypsinization and dilution, the samples were loaded onto a reverse-phase column. The desalted peptides were then separated and submitted to LC-MS/MS analysis. Details are available in Supplementary Information.

### Co-immunoprecipitation (co-IP) assay

Protein lysates for co-IP were extracted using NET buffer (50 mM Tris-HCl pH = 7.5, 5 mM EDTA pH = 8, 150 mM NaCl, 0.5% Triton X-100). One mg of lysate was incubated with 1–2 μg of antibody and 30 μL of protein-A Sepharose (GE Healthcare, Chicago, IL, USA, Cat: 17-0469-01) with constant rotation at 4 °C for over 12 h. To examine the association between PHB2 and IGFBP6, 50 ng of recombinant IGFBP6 with His-tag (SinoBiological, 13026-H08H) was supplemented to the lysate. After incubation, the beads were washed 5-times with pre-chilled NET buffer and then used for WB analysis.

### Cytosol, membranous, and mitochondrial fractionation

The Mem-PER Plus Membrane Protein Extraction kit (Thermo Scientific, Waltham, MA, USA, Cat: 89842) was used to isolate different subcellular fractionations according to the manufacturer’s instructions. Details are available in Supplementary Information.

### siRNA-mediated knockdown of PHB2

Three siPHB2 RNA oligonucleotides were used: siPHB2#1: CCCAGGUAUCCCUGUUGAUTT, siPHB2#2: GCCACAUCACAGAAUCGUATT, and siPHB2#3: CCAACCCAGGAA-UUCUCAATT. Lipofectamine RNAiMax (Invitrogen, 13778150) was applied to transfect siRNA according to the manufacturer’s instructions.

### Statistical analysis

Details are available in Supplementary Information. All statistical analysis were performed using the Statistical Package for the Social Sciences (SPSS) statistics Version 20.

## Supplementary information

Reproducibility checklist

Supplementary information

Original Data File

## Data Availability

The authors declare that all relevant data of this study are available within the article and its supplementary files or from the corresponding author on reasonable request.
