# Supplementary material for: GALNT14-mediated O-glycosylation on PHB2 serine-161 enhances cell growth, migration and drug resistance by activating IGF1R cascade in hepatoma cells
Source: Cell Death Dis. 2022 Nov 14;13(11):956. doi: 10.1038/s41419-022-05419-y (PMC9663550; doi:10.1038/s41419-022-05419-y)

Supplementary Information

Supplementary Materials and Methods

GALNT14-rs9679162 genotyping

Analysis of GALNT14-rs9679162 genotypes was conducted as previously described [1]. Briefly, the genomic DNA was isolated from noncancerous tissues derived from HCC patients using QIAamp DNA mini kit (QIAGEN, Cat: 51306) according to the instruction provided by manufacture. Totally 100-500 ng genomic DNA per reaction was used. The primers, GALNT14-F1: 5'-TCACGAGGCCAACATTCTAG-3' and GALNT14-R1: 5'-TTAGATTCTGCATGGCTCAC-3', were used for amplification of the SNP-containing DNA fragment. The PCR product was directly submitted to Sanger's sequencing for genotyping.

Lysate preparation and western blot (WB)

The protein lysate preparation and WB was conducted as described previously [2]. Briefly, for each lane, 20 μg denatured protein was loaded and separated by the 10% bis-Tris buffered polyacrylamide gel. The rabbit polyclonal antibodies against GALNT14 (Proteintech, Cat: 16939-1-AP, 1:1000 dilution), VDAC1 (Protintech, Cat: 55259-1-AP, 1:1000 dilution), Calnexin (GeneTex, Cat: GTX109669, 1:5000 dilution), PHB2 (Proteintech, Cat: 12295-1-AP, 1:2000 dilution), ERK1/2 (Abclonal, Cat: A166686, 1:1000 dilution), STAT3 (Proteintech, Cat: 10253-2-AP, 1:2000 dilution), the rabbit monoclonal antibodies against GAPDH (Proteintech, Cat: 60004-1-Ig), AKT1 (Abclonal, Cat: A17909, 1:1000 dilution), phospho-AKT1-ser473 (Cell Signaling Technology, Cat: #4060, 1:1000 dilution), phospho-ERK1/2-Thr202/Tyr204 (Cell Signaling Technology, Cat: #4370, 1:1000 dilution), phospho-STAT3-Tyr705 (Cell Signaling Technology, Cat: #9145, 1:1000 dilution), IGF1R (Cell Signaling Technology, Cat: #9760, 1:1000 dilution), phospho-IGF1R-Tyr1135/1136 (Cell Signaling Technology, Cat: #3024, 1:500 dilution), and the mouse monoclonal antibody against IGFBP6 (Santa Cruz Technology, Cat: sc-293295, 1:500 dilution) and CD175/Tn antigen (Aviva Systems Biology, Cat: OASA01388) were used.

Immunohistochemical staining (IHC)

The IHC was conducted as mentioned previously [3]. The rabbit polyclonal antibodies against GALNT14 (Proteintech, Cat: 16939-1-AP) and PHB2 (Proteintech, Cat: 12295-1-AP) were utilized in 1:100 dilution.

Plasmid construction

The primers, EcoRI-GALNT14-F: GGTGGAATTCATGCGGCGCCTGACTCGTCG and NotI-GALNT14-R: TCGAGCGGCCGCCAGAGCTCACCATGTC, were used to amplify *GALNT14* open reading frame (ORF). The underlined sequences were respectively for EcoRI and NotI. The restriction enzymes-digested ORF and pcDNA3.1/V5-HisB (Invitrogen, Cat: V81020) expression vector was ligated using T4 DNA ligase (New England Biolabs, Cat: M0202) to generate the wild type GALNT14 expression vector. To acquire the GALNT14 transferase mutant, containing D199A, H201A, and E203A, as previously described [4], the GALNT14-mutant-F: TCCTCGCCAGCGCCTGTGCGGTGAACAGGGACT primer and QuickChange II site-directed mutagenesis kit (Agilent Technologies, Cat: 200523) were employed according to the manufacture provided instructions. The PHB2 ORF was amplified using primers, EcoRI-PHB2-F: ATCGGAATTCATGGCCCAGAACTTGAAGGA and XhoI-PHB2-R: ATCGCTCGAGTTTCTTACCCTTGATGAGGCT. The underlined sequences were respectively for EcoRI and XhoI. The restriction enzymes-digested ORF and pCMV6-Entry (Origene, Cat: PS100001) expression vector was ligated using T4 DNA ligase to generate the wild type PHB2 expression vector. Mutagenesis for obtaining PHB2-S161A, -T288A, and -S291A was conducted using S161A-mutation: ACCCAGCGGGCCCAGGTAGCCCTGTTGATCCGCCGGGA, T288A-mutation: TACAGGATGAAAGTTTCGCCAGGGGAAGTGACAGCCTCA, and S291A-mutation: TGAAAGTTTCACCAGGGGAGCTGACAGCCTCATCAAG-GGT primers and aforementioned mutagenesis kit. All the expression vectors were sequence verified.

Microsome isolation

The microsome isolation was performed using Microsome Isolation kit (BioVision, Cat: K249) according to the manufacture provided instructions with certain modifications. Briefly, cells harvested from a 10-cm cultural dish were resuspended in 300 μL homogenization buffer and transferred to a pre-chilled Dounce homogenizer. After gently homogenizing the samples with 15 strokes, additional 900 μL homogenization buffer was supplemented to the homogenizer. Followed pipetting and transferring the homogenate to a new tube, an aliquot with 300 μL was preserved as “whole lysate”. The rest was subjected into centrifugation with 10000 *g* for 15 minutes at 4°C. The

supernatant was transferred to a new tube and another 300 μL aliquot of it was also preserved and named as "S9 fraction". The rest was then centrifuged at 22000 *g* for 20 minutes at 4°C. The supernatant, roughly 550 μL, was transferred to a new tube named as "microsome-free supernatant". The pellet was washed by the homogenization buffer once. Followed removing the supernatant, the pellet was resuspended by 550 μL storage buffer and named as "microsome". The protein concentration of the whole lysate was determined. The equal volume of the S9 fraction, microsome-free supernatant, and microsome was used for subsequent analysis.

Liquid-chromatography (LC)-MS/MS analysis

The excised gel spot was first de-stained, and then reduced with 10 mM dithiothreitol (DTT) at 60°C for 45 minutes, followed by cysteine-blocking with 55 mM iodoacetamide (IAM) at 25°C for 30 minutes. Samples were digested with modified porcine trypsin at 37°C for 16-hours. The peptides were extracted from the gel and diluted in HPLC buffer A (0.1% formic acid), and then loaded onto a reverse-phase column (Zorbax 300SB-C18, 0.3 × 5 mm; Agilent Technologies). The desalted peptides were then separated on a homemade column (Waters BEH 1.7 μm, 100 μm I.D. × 10 cm with a 15 μm tip) using a multi-step gradient of HPLC buffer B (99.9% acetonitrile/0.1% formic acid) for 70 minutes with a flow rate of 0.3 μl/min. The LC apparatus was coupled with a 2D linear ion trap mass spectrometer (Orbitrap Elite ETD; Thermo Fisher) operated using Xcalibur 2.2 software (Thermo Fisher). The full-scan MS was performed in the Orbitrap over a range of 400 to 2,000 Da and a resolution of 120,000 at m/z 400. Internal calibration was performed using the ion signal of protonated dodecamethylcyclohexasiloxane ion at m/z 536.165365 as lock mass. The 20 data- dependent MS/MS scan events were followed by one MS scan for the 20 most abundant precursor ions in the preview MS scan. The m/z values selected for MS/MS were dynamically excluded for 40 seconds with a relative mass window of 15 ppm. The electrospray voltage was set to 2.0 kV, and the temperature of the capillary was set to 200°C. MS and MS/MS automatic gain control were set to 1,000 ms (full scan) and 200 ms (MS/MS), or 3×10^6 ions (full scan) and 3,000 ions (MS/MS) for maximum accumulated time or ions, respectively. The data analysis was carried out using Proteome Discoverer software (version 1.4, Thermo Fisher Scientific). The MS/MS spectra were searched against the Swissprot database using the Mascot search engine

(Matrix Science, London, UK; version 2.5). For peptide identification, 10 ppm mass tolerance was permitted for intact peptide masses, and 0.5 Da for CID fragment ions with allowance for two missed cleavages made from the trypsin digestion: oxidized (methionine) and acetyl (protein N-terminal) as variable modifications; carbamidomethyl (cysteine) as static modification. Peptide-spectrum match (PSM) were then filtered based on high confidence and Mascot search engine rank 1 of peptide identification to ensure an overall false discovery rate below 0.01. Proteins with single peptide hit were removed.

Cytosol, membranous, and mitochondrial fractionation

The Mem-PER Plus Membrane Protein Extraction kit (Thermo Scientific, Cat: 89842) was used to isolate different fractionations according to the manufacture provided instructions with certain modifications. Briefly, after 3-times wash of the cells harvested from a 10-cm cultural dish by Cell Wash Solution, 1 mL for each, 300 μL Permeabilization Buffer was added to resuspend cell pellet. Followed 10 minutes constantly rotating at 4°C, it was then centrifuged at 16000 *g* for 15 minutes at 4°C. The supernatant was moved to a new tube named as "Cytosolic protein", and the pellet was resuspended by 300 μL Solubilization Buffer. After 30 minutes constantly rotating at 4°C, it was centrifuged at 4000 *g* for 15 minutes at 4°C. The pellet was resuspended by 300 μL RIPA buffer and named as "mitochondrial protein". The supernatant was transferred to a new tube and then centrifuged at 16000 *g* for 15 minutes at 4°C. The supernatant was moved to a new tube and named as "membranous protein". The concentration of the cytosolic protein was determined, and the equal volume of the mitochondrial and membranous proteins were loaded into the same gel analysis.

Statistical analysis

Parametric data were presented as mean ± standard deviation and compared by using a two-sample *t*-test. Dichotomized data were presented as numbers and percentages (%) and compared utilizing Chi-square test. Univariate and following multivariate Cox proportional hazard models were performed to estimate prognosis for clinical factors, genotypes, and RNA variants. In this study, significant factors determined from the univariate analysis could be included for multivariate Cox proportional hazards. The Kaplan–Meier method was performed to estimate the survival probability between the different groups, and the log-rank test was employed. All tests were two-tailed, and the

$P < 0.05$ was considered statistically significant. All statistical analyses were performed using Statistical Package for the Social Sciences (SPSS) statistics Version 20.

Supplementary tables

Table S1. The comparison of clinical parameters between rs9679162 genotypes.

Characteristics	Total patients (n = 300)	rs9679162 genotype		P
		TT (n = 78)	Non-TT (n = 222)	
Age (years), mean ± SD	55.7 ± 13.9	56.1 ± 15.3	55.5 ± 13.5	0.868
Gender, Male, n (%)	234 (78.0%)	61 (78.2%)	173 (77.9%)	0.960
HBsAg positive, n (%)	215 (71.7%)	50 (64.1%)	165 (74.3%)	0.115
Anti-HCV positive, n (%)	79 (26.3%)	19 (24.4%)	60 (27.0%)	0.756
Alcoholism, n (%)	80 (26.7%)	23 (29.5%)	57 (25.7%)	0.613
Cirrhosis, n (%)	184 (61.3%)	47 (60.3%)	137 (61.7%)	0.927
Ascites, n (%)	17 (5.7%)	6 (7.7%)	11 (5.0%)	0.539
Tumor characteristics				
Capsule, n (%)	214 (71.3%)	60 (76.9%)	154 (69.4%)	0.261
Histology grade ≧3, n (%)	160 (53.3%)	44 (56.4%)	116 (52.3%)	0.616
Microvascular invasion, n (%)	93 (31.0%)	15 (19.2%)	78 (35.1)	**0.014**
Macrovascular invasion, n (%)	33 (11.0%)	9 (11.5%)	24 (10.8%)	0.860
Tumor number, mean ± SD	1.7 ± 1.1	1.6 ± 1.0	1.7 ± 1.1	0.162
Largest tumor size (diameter, cm), mean ± SD	5.7 ± 3.9	5.9 ± 4.0	5.7 ± 3.9	0.673
Biochemistry				

AFP (ng/mL), median (range)	25 (1.0 – 685353.0)	23 (1.1 – 286979.0)	25 (1.0 – 685353.0)	0.998
Albumin (g/dL), mean ± SD	4.0 ± 0.6	4.0 ± 0.6	4.0 ± 0.6	0.718
Bilirubin (mg/dL), mean ± SD	1.1 ± 1.6	1.4 ± 2.6	1.0 ± 1.0	0.683
Prothrombin time (sec), mean ± SD	12.1 ± 1.4	11.9 ± 1.3	12.1 ± 1.4	0.456
Creatinine (mg/dL), mean ± SD	1.2 ± 1.1	1.1 ± 0.6	1.2 ± 1.2	0.864
AST (U/L), mean ± SD	65.5 ± 75.4	67.0 ± 66.9	65.0 ± 78.4	0.791
ALT (U/L), mean ± SD	68.8 ± 79.9	69.4 ± 78.4	68.7 ± 80.6	0.599

Bold value indicates statistical significance. $P < 0.05$ was considered as significant. AFP, alpha-fetoprotein; AST, aspartate transaminase; ALT, alanine transaminase.

Table S2. Cox regression analysis to estimate the association between clinical parameters, rs9679162 genotypes, presence of *GALNT14* variants, GALNT14 expression, or PHB2 levels and OS.

	Univariate			Multivariate		
Clinical parameters	HR	95% CI	P	HR	95% CI	P
Age, per year increase	0.988	0.963 – 1.014	0.351			
Gender, Male = 1	0.715	0.329 – 1.554	0.397			
HBsAg, Positive = 1	1.072	0.479 – 2.399	0.866			
Anti-HCV, Positive = 1	0.661	0.271 – 1.612	0.363			
Alcoholism, Yes = 1	0.969	0.433 – 2.168	0.939			
Cirrhosis, Yes = 1	1.745	0.780 – 3.901	0.175			
Ascites, Yes = 1	3.139	1.097 – 8.983	**0.033**	1.581	0.528 – 4.736	0.413
Tumor characteristics						
Capsule, Yes = 1	0.718	0.344 – 1.499	0.378			
Histological grade, per grade increase	1.038	0.618 – 1.746	0.887			
Microvascluar invasion, Yes = 1	1.986	0.971 – 4.059	0.060			
Macrovascular invasion, Yes = 1	3.059	1.306 – 7.074	**0.010**	1.642	0.613 – 4.402	0.324
Tumor number, per number increase	1.398	1.072 – 1.821	**0.013**	1.380	1.016 – 1.873	**0.039**
Tumor size, per cm increase	1.058	0.973 – 1.150	0.187			
Biochemistry						

AFP, per 1000 ng/mL increase	0.998	0.989 – 1.008	0.752			
Albumin, per g/dL increase	0.817	0.428 – 1.559	0.541			
Bilirubin, per mg/dL increase	1.046	0.860 – 1.273	0.652			
Prothrombin time, per sec increase	1.067	0.839 – 1.355	0.598			
Creatinine, per mg/dL increase	0.992	0.743 – 1.323	0.955			
AST, per U/L increase	1.001	0.995 – 1.006	0.766			
ALT, per U/L increase	0.997	0.990 – 1.003	0.332			
Rs9679162 genotype, TT = 1	0.494	0.190 – 1.288	0.149			
GALNT14 level, C/N>1 = 1	3.079	1.368 – 6.933	**0.007**	0.969	0.346 – 2.711	0.952
GALNT14 long variant in N, Yes = 1	0.750	0.308 – 1.829	0.528			
GALNT14 long variant in C, Yes = 1	0.701	0.314 – 1.569	0.388			
GALNT14 short variant in N, Yes = 1	0.130	0.045 – 0.373	**<0.001**	0.564	0.133 – 2.399	0.438
GALNT14 short variant in C, Yes = 1	0.092	0.028 – 0.306	**<0.001**	0.168	0.031 – 0.901	**0.037**
PHB2 level, C/N>1 = 1	2.817	1.293 – 6.135	**0.009**	1.562	0.598 – 4.083	0.363

Bold value indicates statistical significance. $P < 0.05$ was considered as significant. AFP, alpha-fetoprotein; AST, aspartate transaminase; ALT, alanine transaminase; N, noncancerous tissue; C, cancerous tissue; HR, hazard ratio; CI, confidence interval.

Table S3. Cox regression analysis to estimate the association between clinical parameters, rs9679162 genotypes, presence of *GALNT14* variants, GALNT14 expression, or PHB2 levels and RFS.

	Univariate			Multivariate		
Clinical parameters	HR	95% CI	P	HR	95% CI	P
Age, per year increase	1.001	0.989 – 1.012	0.910			
Gender, Male = 1	1.017	0.706 – 1.466	0.927			
HBsAg, Positive = 1	0.950	0.678 – 1.332	0.766			
Anti-HCV, Positive = 1	1.044	0.737 – 1.478	0.810			
Alcoholism, Yes = 1	1.013	0.721 – 1.425	0.939			
Cirrhosis, Yes = 1	1.521	1.096 – 2.110	**0.012**	1.201	0.818 – 1.763	0.350
Ascites, Yes = 1	2.755	1.588 – 4.780	**<0.001**	1.742	0.967 – 3.140	0.065
Tumor characteristics						
Capsule, Yes = 1	1.076	0.769 – 1.507	0.668			
Histological grade, per grade increase	1.265	1.021 – 1.567	**0.032**	1.118	0.871 – 1.434	0.407
Microvascluar invasion, Yes = 1	2.674	1.960 – 3.647	**<0.001**	2.088	1.490 – 2.926	**<0.001**
Macrovascular invasion, Yes = 1	1.752	1.127 – 2.726	**0.013**	0.930	0.556 – 1.556	0.783
Tumor number, per number increase	1.200	1.047 – 1.375	**0.009**	1.187	1.020 – 1.381	**0.026**
Tumor size, per cm increase	1.086	1.046 – 1.127	**<0.001**	1.048	0.999 – 1.099	0.054
Biochemistry						

AFP, per 1000 ng/mL increase	1.002	0.999 – 1.004	0.197			
Albumin, per g/dL increase	0.669	0.513 – 0.873	**0.003**	0.865	0.614 – 1.219	0.408
Bilirubin, per mg/dL increase	0.946	0.817 – 1.094	0.451			
Prothrombin time, per sec increase	1.126	1.026 – 1.237	**0.012**	0.986	0.866 – 1.122	0.831
Creatinine, per mg/dL increase	0.983	0.833 – 1.159	0.837			
AST, per U/L increase	1.003	1.002 – 1.005	**<0.001**	1.004	1.001 – 1.007	**0.017**
ALT, per U/L increase	1.002	1.000 – 1.003	**0.022**	0.998	0.996 – 1.001	0.173
Rs9679162 genotype, TT = 1	0.646	0.446 – 0.936	**0.021**	0.883	0.594 – 1.313	0.538
GALNT14 level, C/N>1 = 1	3.340	2.370 – 4.708	**<0.001**	1.637	1.047 – 2.559	**0.031**
GALNT14 long variant in N, Yes = 1	0.779	0.515 – 1.176	0.235			
GALNT14 long variant in C, Yes = 1	0.969	0.661 – 1.421	0.872			
GALNT14 short variant in N, Yes = 1	0.216	0.150 – 0.311	**<0.001**	0.614	0.361 – 1.046	0.073
GALNT14 short variant in C, Yes = 1	0.143	0.096 – 0.213	**<0.001**	0.248	0.140 – 0.439	**<0.001**
PHB2 level, C/N>1 = 1	2.261	1.646 – 3.105	**<0.001**	0.932	0.625 – 1.390	0.729

Bold value indicates statistical significance. $P < 0.05$ was considered as significant. AFP, alpha-fetoprotein; AST, aspartate transaminase; ALT, alanine transaminase; N, noncancerous tissue; C, cancerous tissue; HR, hazard ratio; CI, confidence interval.

Table S4. Cox regression analysis to estimate the association between clinical parameters, rs9679162 genotypes, presence of *GALNT14* variants, GALNT14 expression, or PHB2 levels and MFS.

	Univariate			Multivariate		
Clinical parameters	HR	95% CI	P	HR	95% CI	P
Age, per year increase	0.984	0.966 – 1.003	0.102			
Gender, Male = 1	1.321	0.665 – 2.624	0.426			
HBsAg, Positive = 1	1.232	0.661 – 2.297	0.512			
Anti-HCV, Positive = 1	0.545	0.267 – 1.113	0.096			
Alcoholism, Yes = 1	0.828	0.444 – 1.545	0.553			
Cirrhosis, Yes = 1	1.168	0.670 – 2.038	0.583			
Ascites, Yes = 1	0.793	0.193 – 3.257	0.747			
Tumor characteristics						
Capsule, Yes = 1	0.930	0.524 – 1.648	0.802			
Histological grade, per grade increase	1.286	0.878 – 1.882	0.197			
Microvascluar invasion, Yes = 1	2.907	1.711 – 4.939	**<0.001**	1.841	1.065 – 3.181	**0.029**
Macrovascular invasion, Yes = 1	3.592	1.952 – 6.611	**<0.001**	2.650	1.342 – 5.231	**0.005**
Tumor number, per number increase	1.438	1.161 – 1.781	**0.001**	1.258	0.972 – 1.628	0.080
Tumor size, per cm increase	1.130	1.072 – 1.191	**<0.001**	1.070	1.003 – 1.143	**0.042**
Biochemistry						

AFP, per 1000 ng/mL increase	1.004	1.002 – 1.007	**0.002**	1.003	1.000 – 1.006	0.057
Albumin, per g/dL increase	0.880	0.542 – 1.428	0.605			
Bilirubin, per mg/dL increase	0.829	0.524 – 1.312	0.423			
Prothrombin time, per sec increase	1.030	0.851 – 1.246	0.761			
Creatinine, per mg/dL increase	0.604	0.243 – 1.501	0.278			
AST, per U/L increase	1.003	1.001 – 1.006	**0.009**	1.002	0.999 – 1.006	0.136
ALT, per U/L increase	0.999	0.995 – 1.003	0.689			
Rs9679162 genotype, TT = 1	0.138	0.043 – 0.442	**0.001**	0.141	0.042 – 0.474	**0.002**
GALNT14 level, C/N>1 = 1	2.872	1.578 – 5.226	**0.001**	1.321	0.614 – 2.841	0.476
GALNT14 long variant in N, Yes = 1	0.981	0.462 – 2.080	0.959			
GALNT14 long variant in C, Yes = 1	1.086	0.547 – 2.158	0.813			
GALNT14 short variant in N, Yes = 1	0.308	0.167 – 0.567	**<0.001**	0.581	0.235 – 1.435	0.239
GALNT14 short variant in C, Yes = 1	0.301	0.161 – 0.560	**<0.001**	0.546	0.208 – 1.436	0.222
PHB2 level, C/N>1 = 1	2.074	1.187 – 3.627	**0.010**	0.879	0.434 – 1.780	0.720

Bold value indicates statistical significance. P < 0.05 was considered as significant. AFP, alpha-fetoprotein; AST, aspartate transaminase; ALT, alanine transaminase; N, noncancerous tissue; C, cancerous tissue; HR, hazard ratio; CI, confidence interval.

Supplementary Figure legends

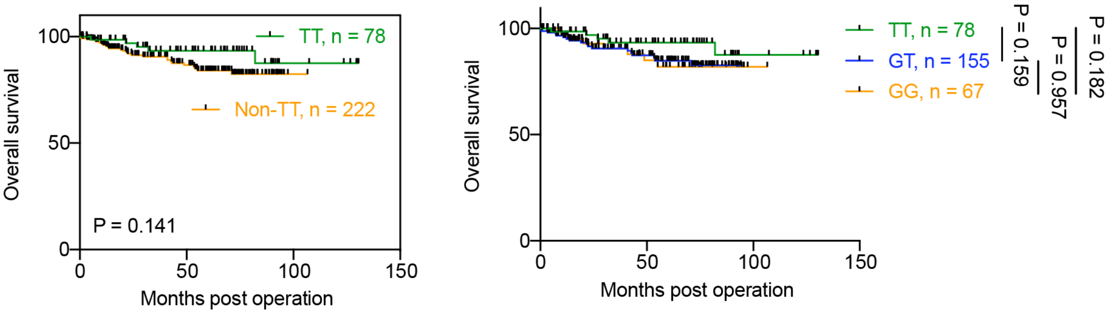

Figure S1. The Kaplan-Meyer analysis of the overall survival rate in HCC patients, stratified according to the rs9679162 genotypes. This figure was related to Figure 1B in the main-text.

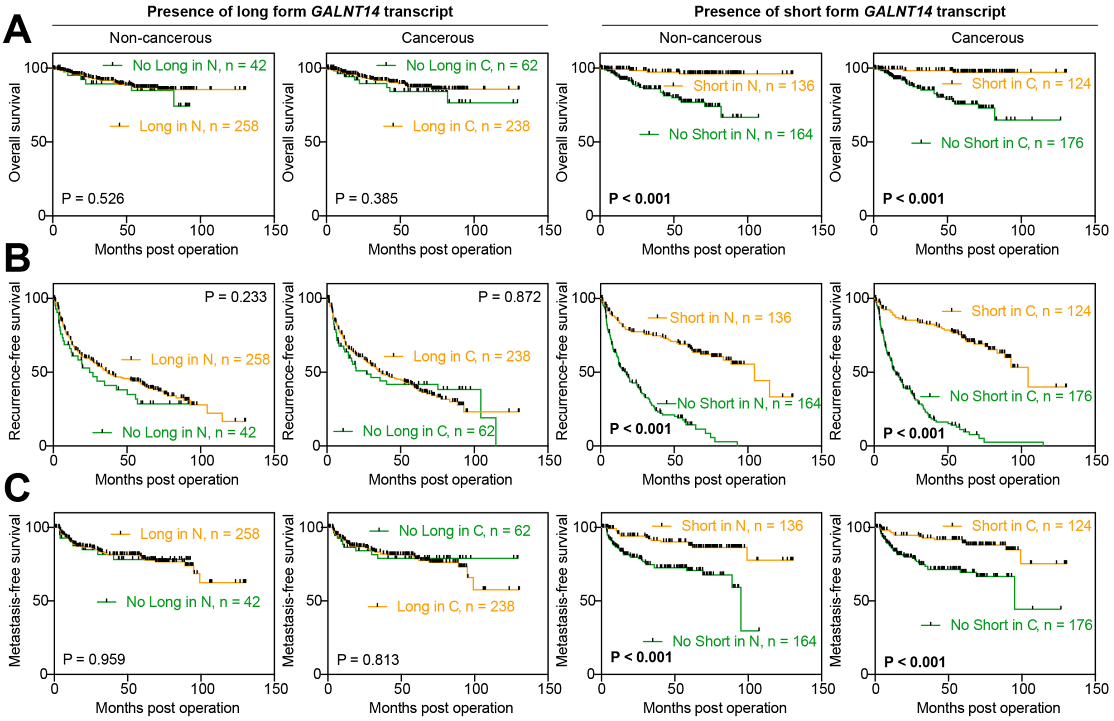

Figure S2. A novel *GALNT14* RNA variant associates with the postoperative clinical outcomes in HCC. The Kaplan-Meier analysis of the (A) overall, (B) recurrence-free, and (C) metastasis-free survival rates, stratified according to the presence of long or short form GALNT14 RNA variant in either noncancerous (N) or cancerous (C) tissue. The P-values were obtained by the log-rank test.

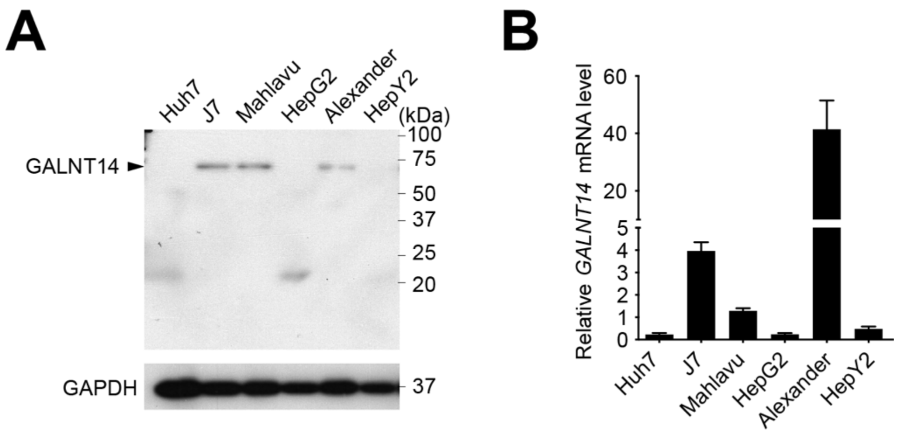

Figure S3. The GALNT14 expression levels in HCC cell lines. (A) The western blot and (B) RT-qPCR analyses of samples from indicated HCC cells. This figure was related to Figure 3 in the main-text.

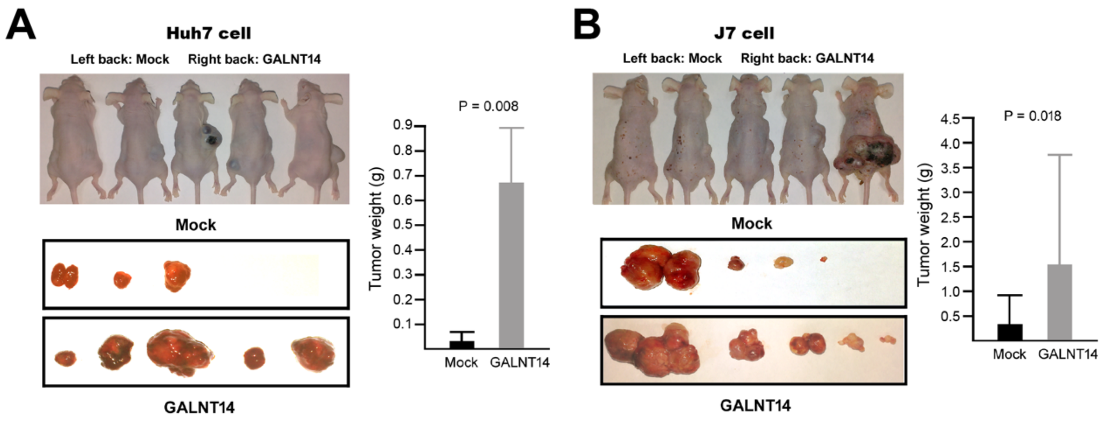

Figure S4. Elevating GALNT14 expression promotes the xenografted tumor formation. The (A) Huh7 and (B) J7 cells with or without GALNT14 overexpression was used and subcutaneously injected into the 6-weeks old nude mice to see the capabilities to form a solid tumor. After sacrifice, the tumors were collected and weighted for comparison as shown in the right panel. The P-values were acquired by using two-tail Mann-Whitney U test. This figure was related to Figure 3 in the main-text.

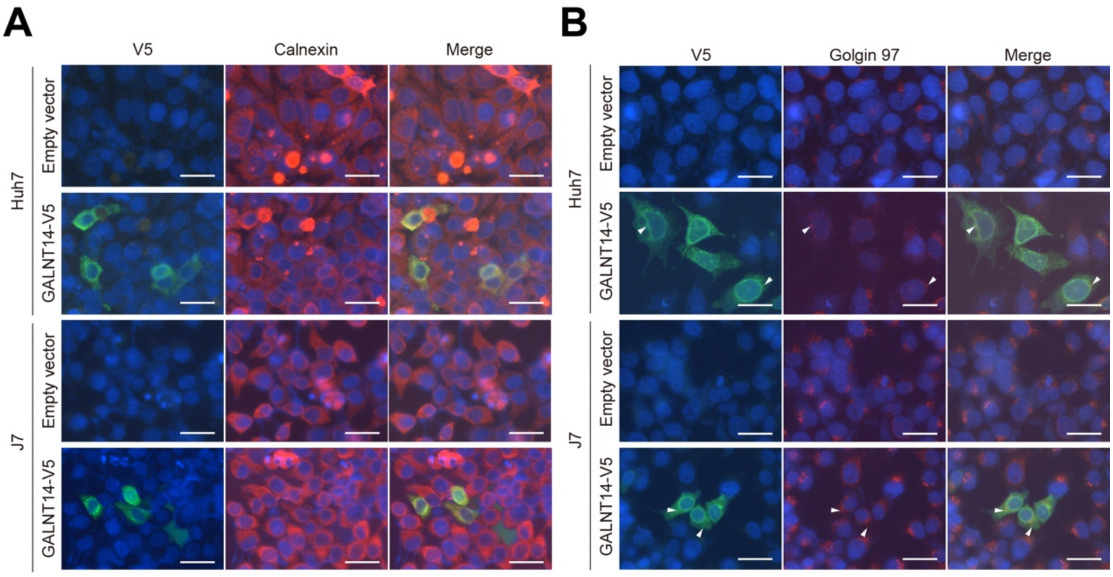

Figure S5. GALNT14 is located in the Golgi-body and endoplasmic reticulum. The immunofluorescent assay showed the localization of GALNT14 in the (A) endoplasmic reticulum and (B) Golgi-body. Calnexin, endoplasmic reticulum marker; Golgin 97, Golgi-body marker. This figure was related to Figure 4A in the main-text.

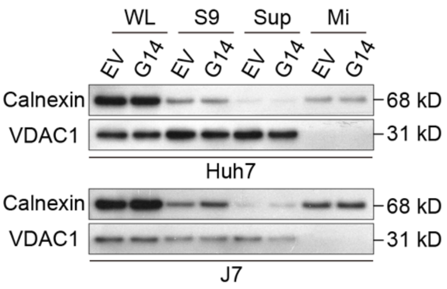

Figure S6. The representative western blot images to confirm the efficacy of micorsomal fractionation. EV, empty vector; G14, GALNT14 expressing vector; WL, whole lysate; S9, S9 fraction; Sup, microsome-free suppernatant; Mi, microsomal fraction.

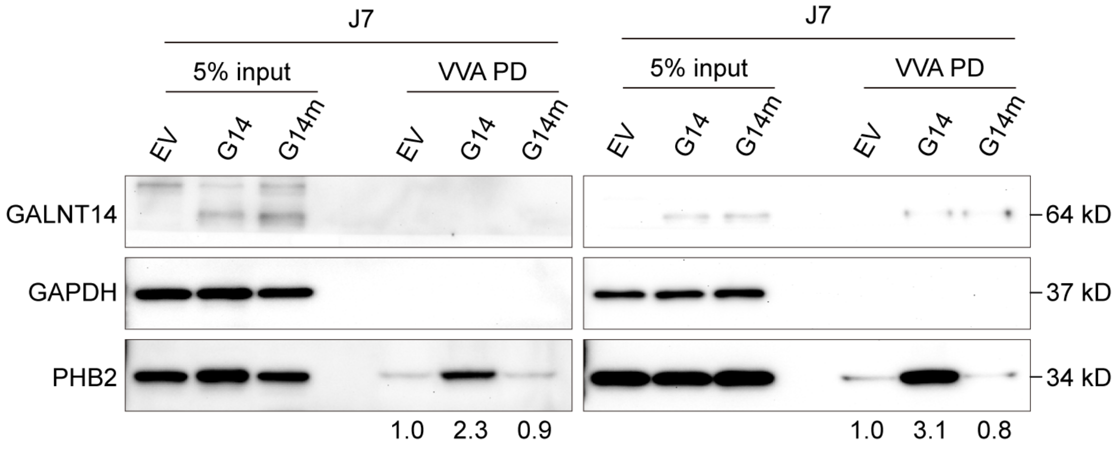

Figure S7. GALNT14 GalNAc-T activity dependent pull-down of PHB2 in HCC cells. The representative western blots to demonstrate the levels of PHB2 enriched by VVA-mediated pull-down in HCC cells with overexpression of GALNT14 (G14) or mutant (G14m).

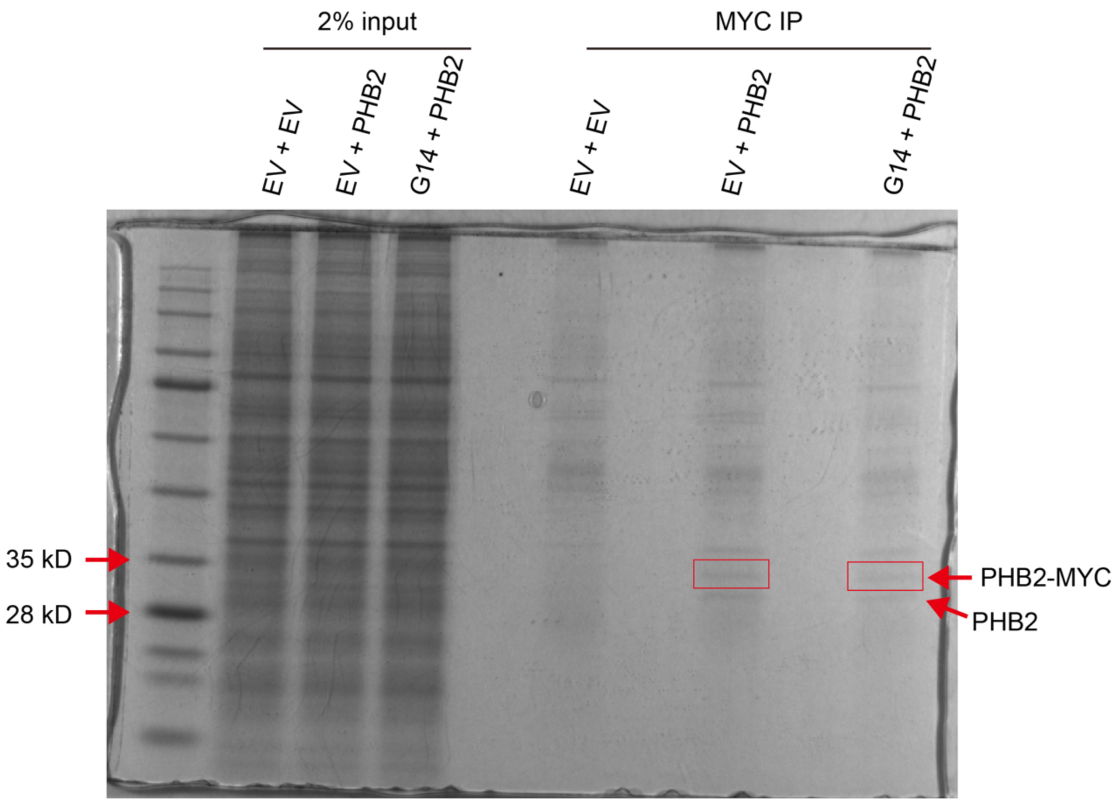

Figure S8. Protein gel for LC/MS/MS analysis. The immunoprecipitation was performed as indicated and the precipitates were separated by the 10% Bis-Tris buffered protein gel. After colloidal blue silver staining, the proteins bands with the red color boxes were excised for LC/MS/MS analysis. This figure was related to Figure 5C in the main-text.

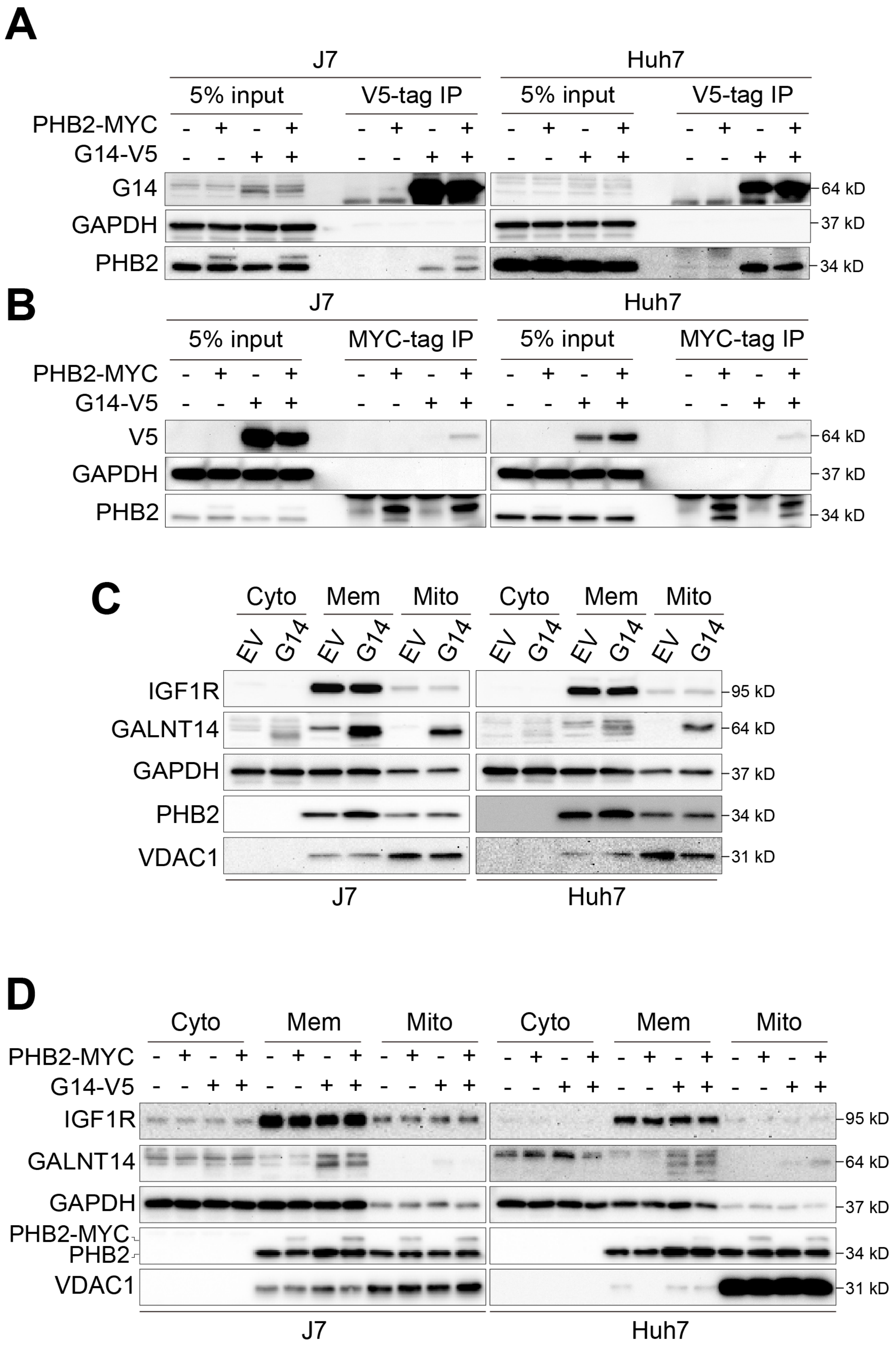

Figure S9. GALNT14 physically interacts with PHB2 and enhances its membranous localization. (A-B) The western blot analysis of the co-immunoprecipitation assay by using samples from HCC cells with indicated treatments. (C-D) The fractionated

fractions, including cytosolic (Cyto), membranous (Mem) and mitochondrial (Mito), derived from cells with indicated treatments were subjected into western blot analysis. IGF1R is a membranous fraction specific marker; while VDAC1 is for the mitochondrial fraction. This figure was related to Figure 5D-F in the main-text.

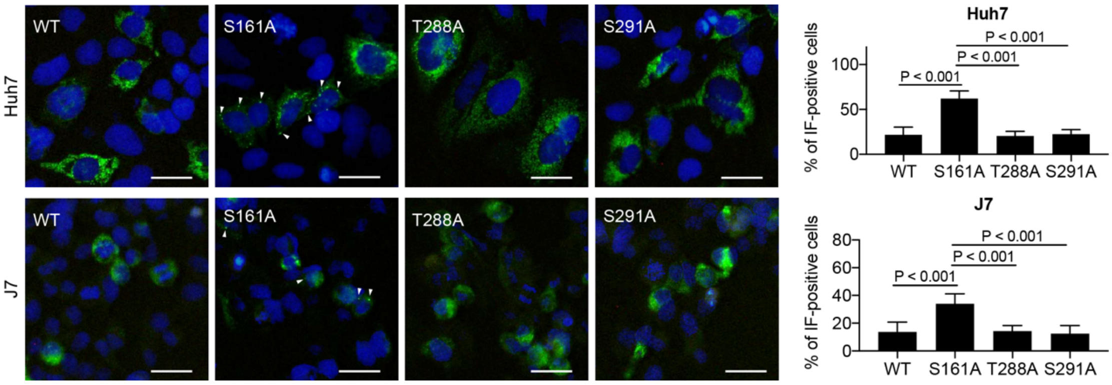

Figure S10. Mutation occurred at S161 residue hampers on the subcellular localization of PHB2 in HCC cells. The immunofluorescent staining of PHB2 were performed using indicated HCC cells with indicated treatments. This figure was related to Figure 5F in the main-text.

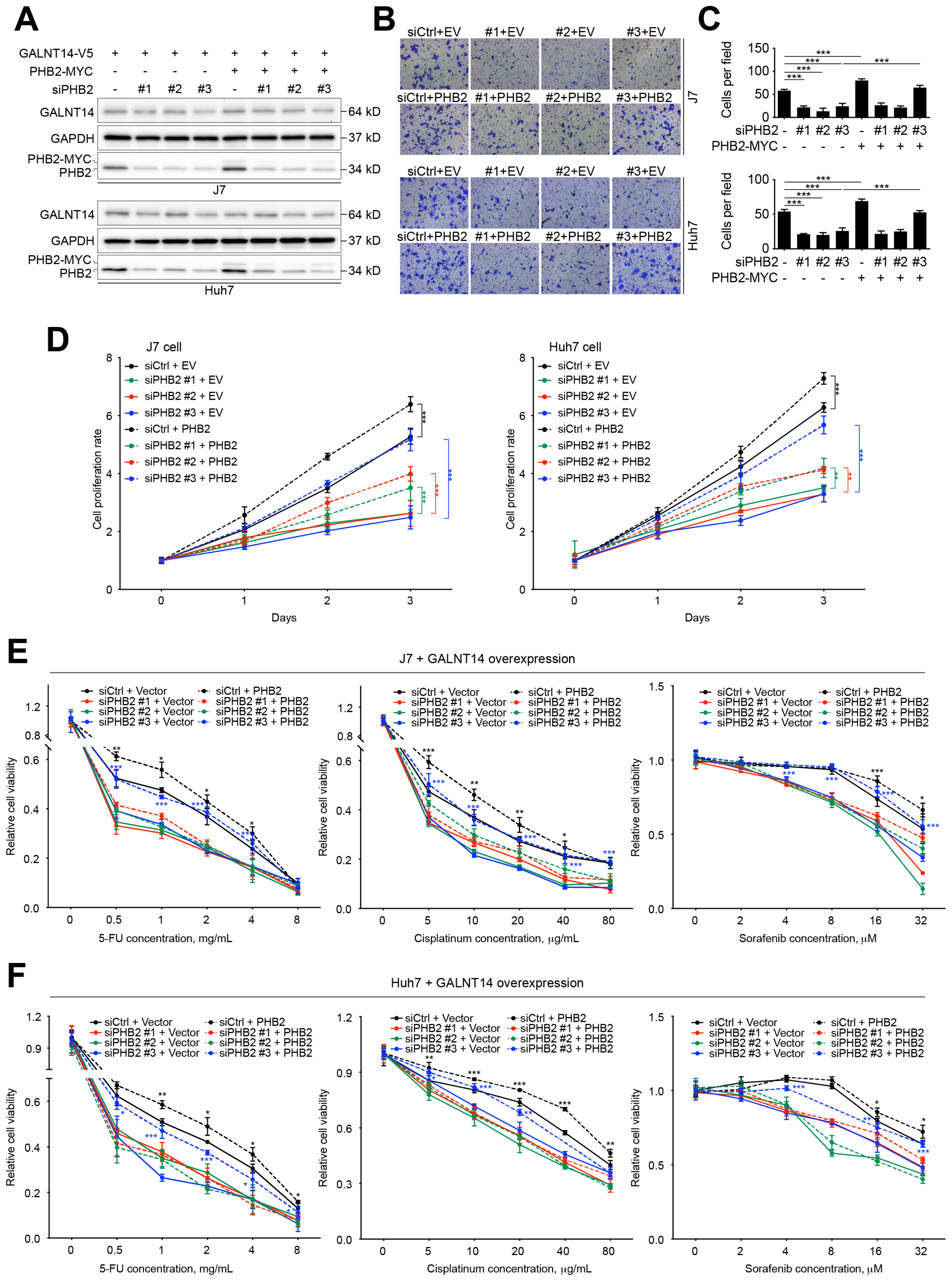

Figure S11. Altered PHB2 expression influences the GALNT14-modulated phenotypes in HCC. (A) The western blot analysis of lysates derived from cells with indicted treatments. pCMV6, empty vector. pCMV6-PHB2, plasmid for expression of PHB2-MYC protein. (B) The representative images of the transwell-based migration. The quantitative result of the migratory cells was shown in (C). The P-values were obtained by using two-tail Mann-Whitney U test. (D) The alarmar blue-based cell

growth assays by using cells with indicated treatments. The P-values were obtained by using the two-way ANOVA. (E-F) The relative cell viabilities of anticancer drug-treated HCC cells with indicated treatments. Three independent anticancer drugs were tested, including 5-FU, Cisplatin, Sorafenib. The P-values at each concentration were obtained by using the unpaired two-tail student *t*-test. *, P<0.05; **, P<0.01; ***, P< 0.001. This figure was related to Figure 6 in the main-text.

Supplementary references:

1 Lin WR, Hsu CW, Yeh CS, Chen YC, Chang ML, Liang KH *et al*. Combinations of single nucleotide polymorphisms WWOX-rs13338697, GALNT14-rs9679162 and rs6025211 effectively stratify outcomes of chemotherapy in advanced hepatocellular carcinoma. Asia Pac J Clin Oncol. 2018;14:e54-63.

2 Chu YD, Lai HY, Pai LM, Huang YH, Lin YH, Liang KH *et al*. The methionine salvage pathway-involving ADI1 inhibits hepatoma growth by epigenetically altering genes expression via elevating S-adenosylmethionine. Cell Death Dis. 2019;10:240.

3 Chu YD, Lin WR, Lin YH, Kuo WH, Tseng CJ, Lim SN *et al*. COX5B-Mediated Bioenergetic Alteration Regulates Tumor Growth and Migration by Modulating AMPK-UHMK1-ERK Cascade in Hepatoma. Cancers (Basel). 2020;12;1646.

4 Song KH, Park MS, Nandu TS, Gadad S, Kim SC, Kim MY. GALNT14 promotes lung-specific breast cancer metastasis by modulating self-renewal and interaction with the lung microenvironment. Nat Commun. 2016;7:13796.

Original western blots for Fig 1C

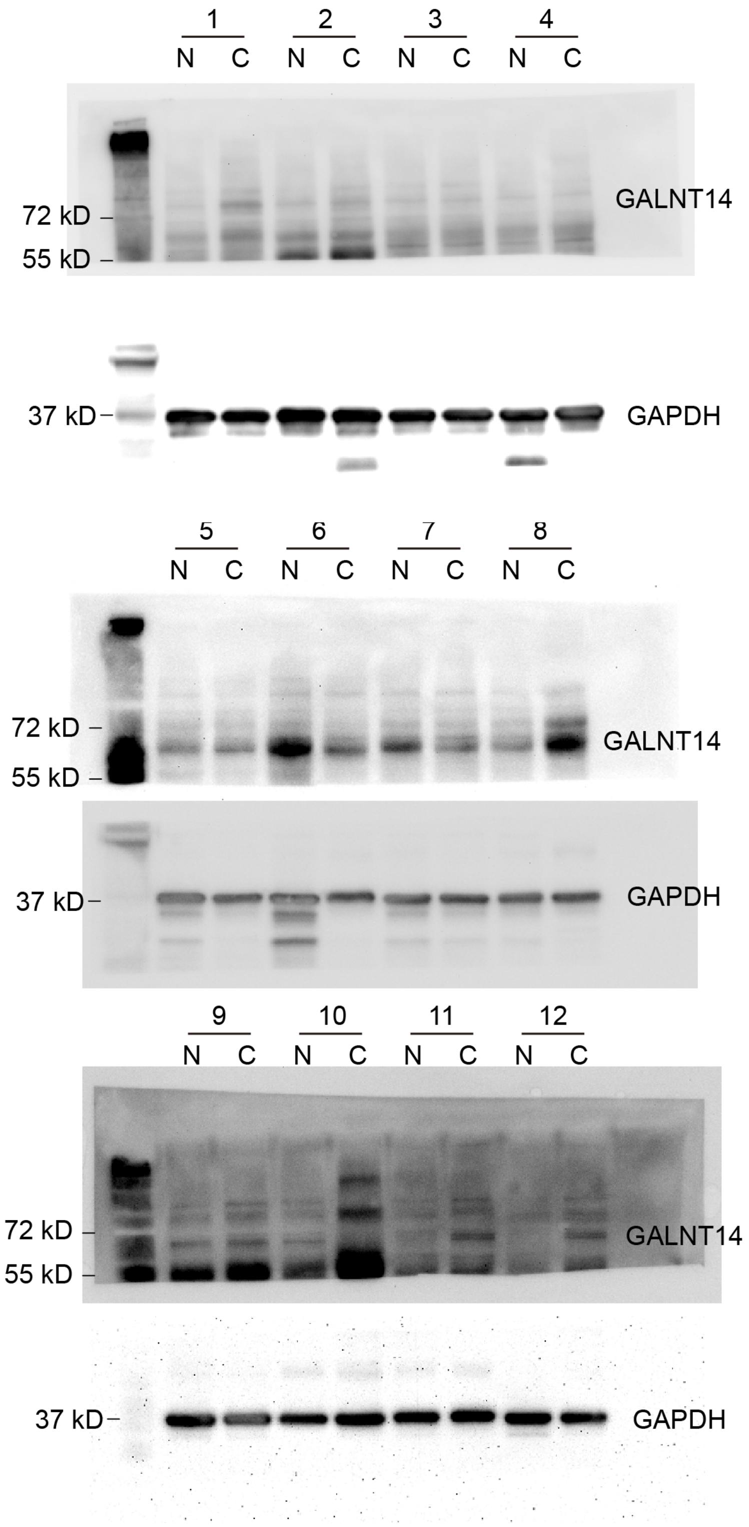

Original western blots for Fig 2A

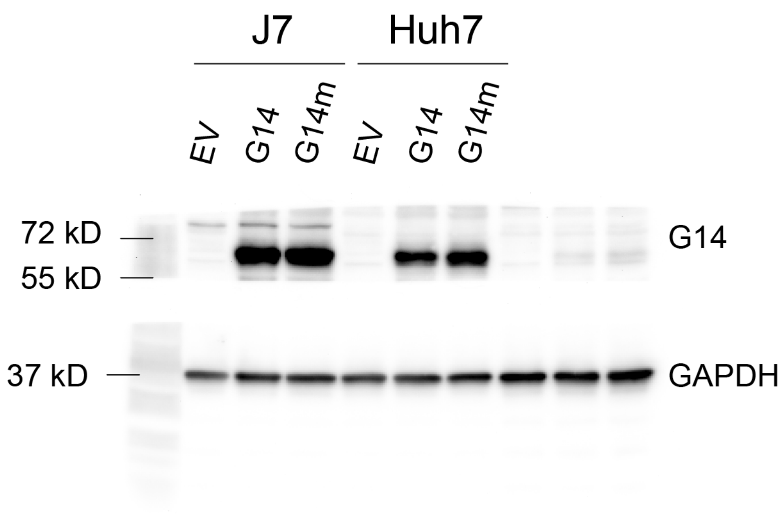

Original western blots for Fig 2C

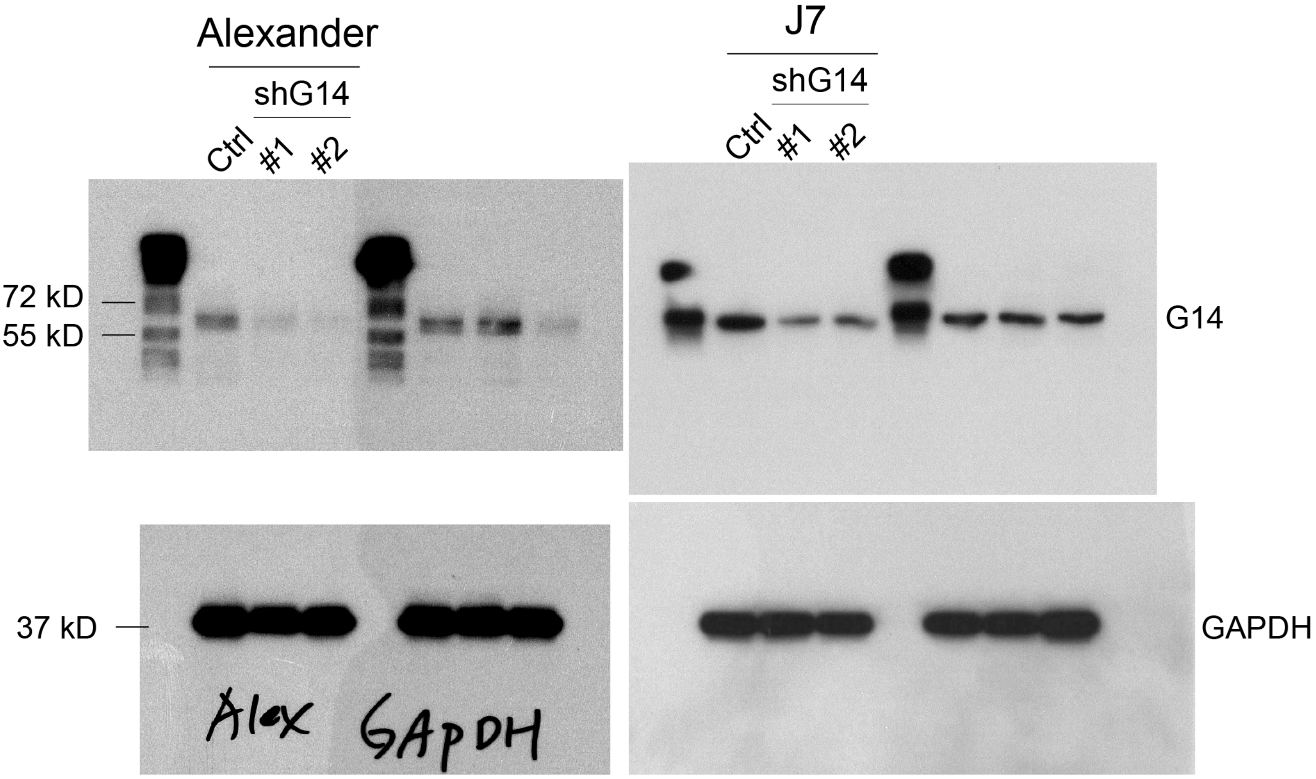

Original western blots for Fig 3B

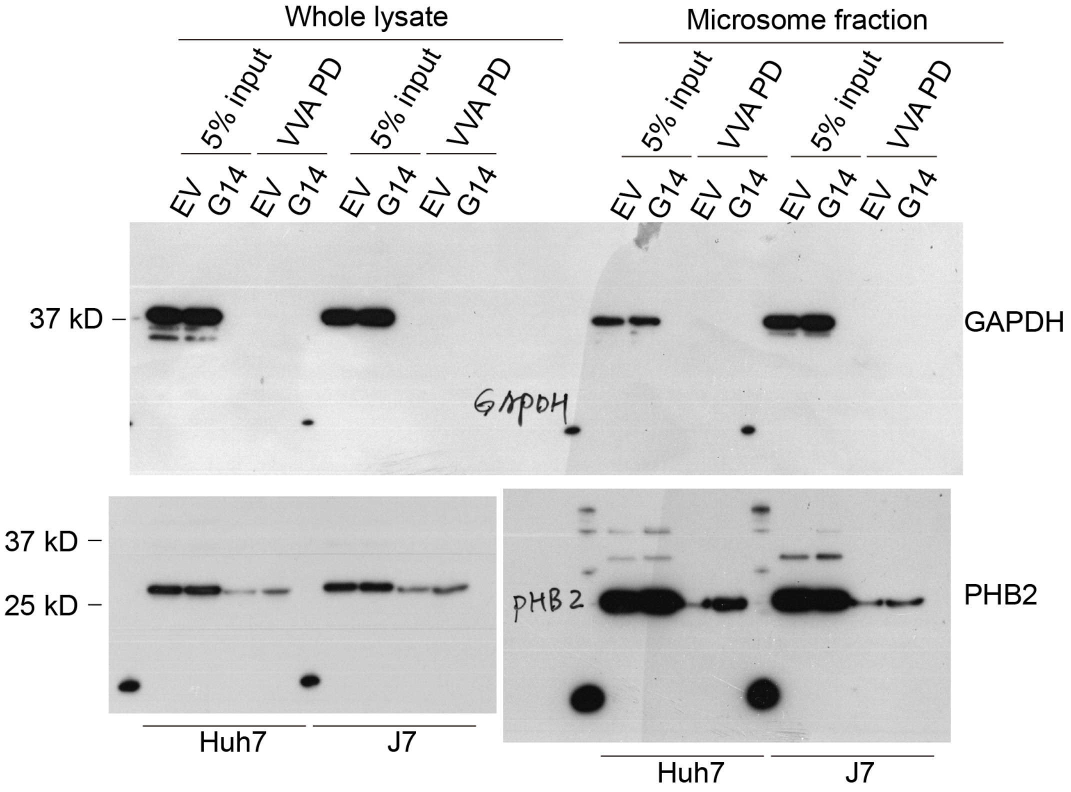

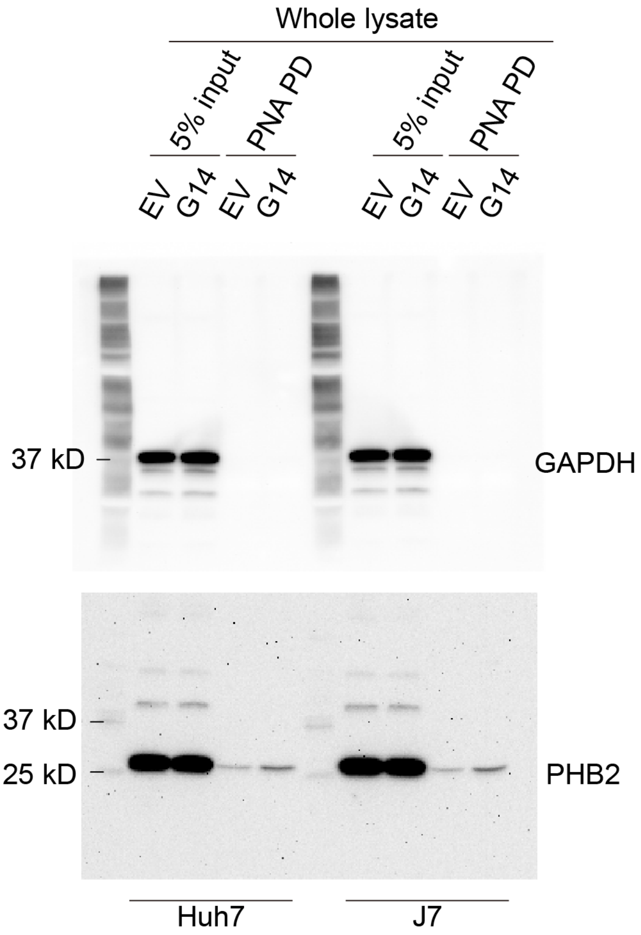

Original western blots for Fig 3C

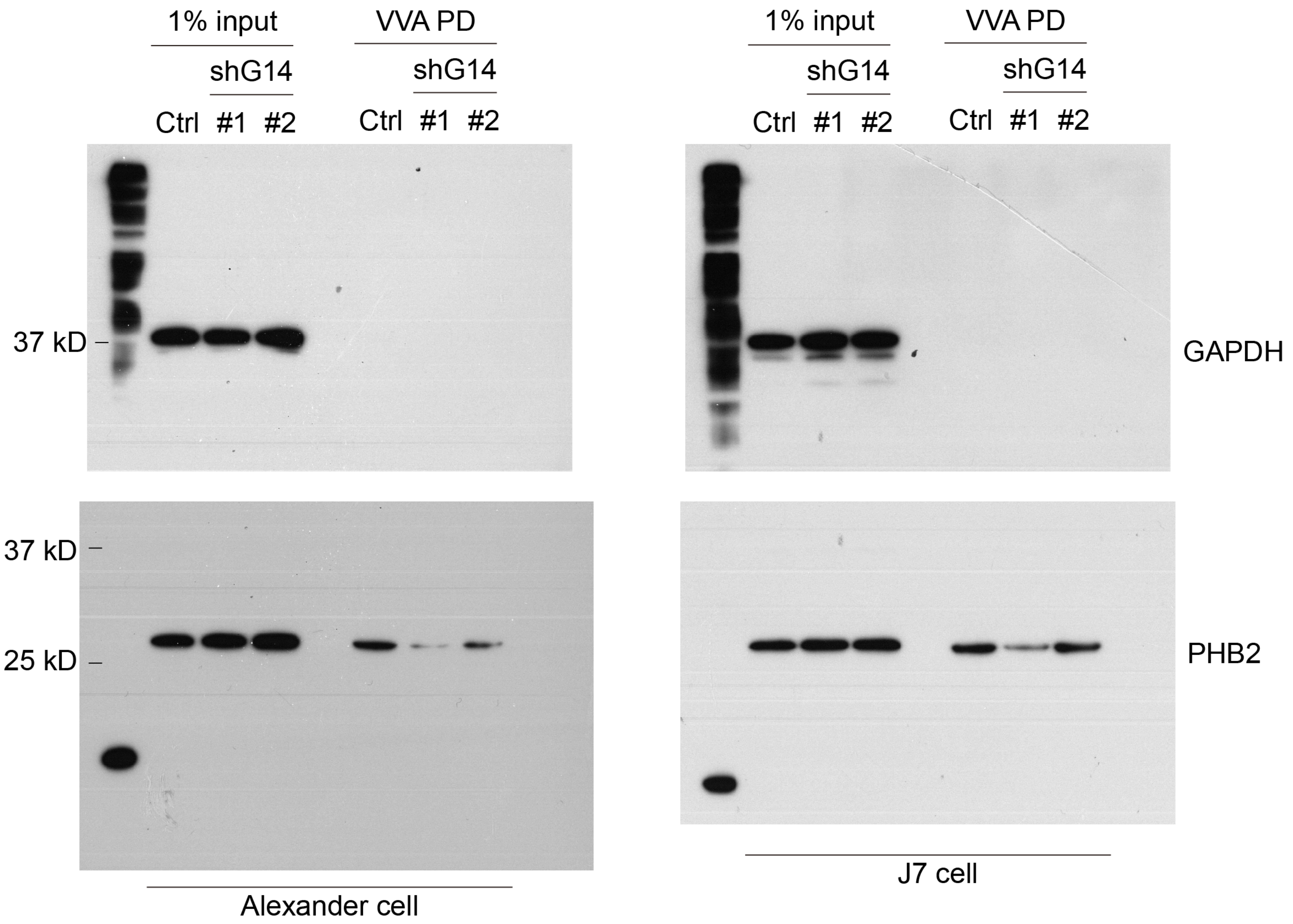

Original western blots for Fig 3D

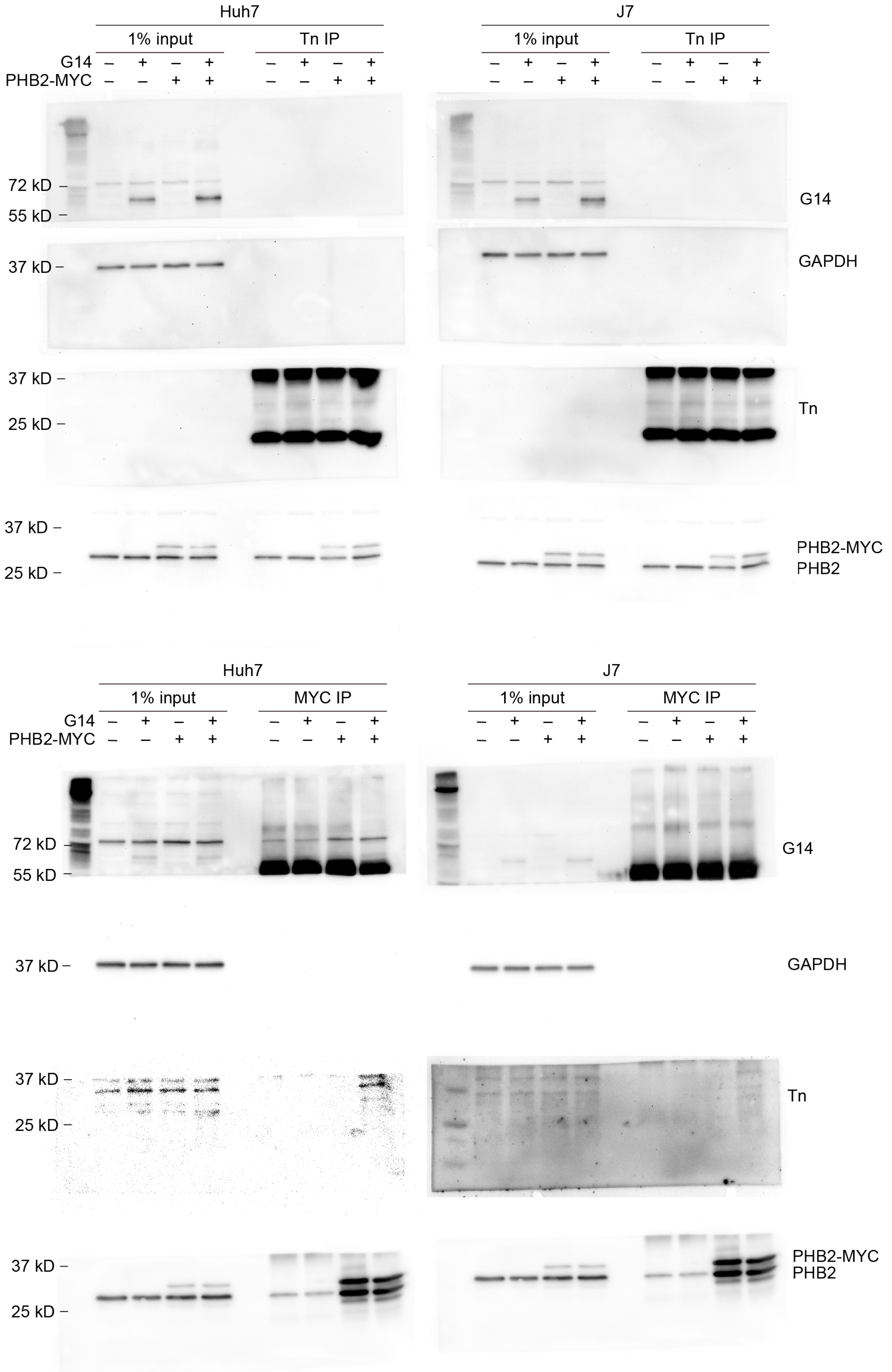

Original Western blots for Fig 3E

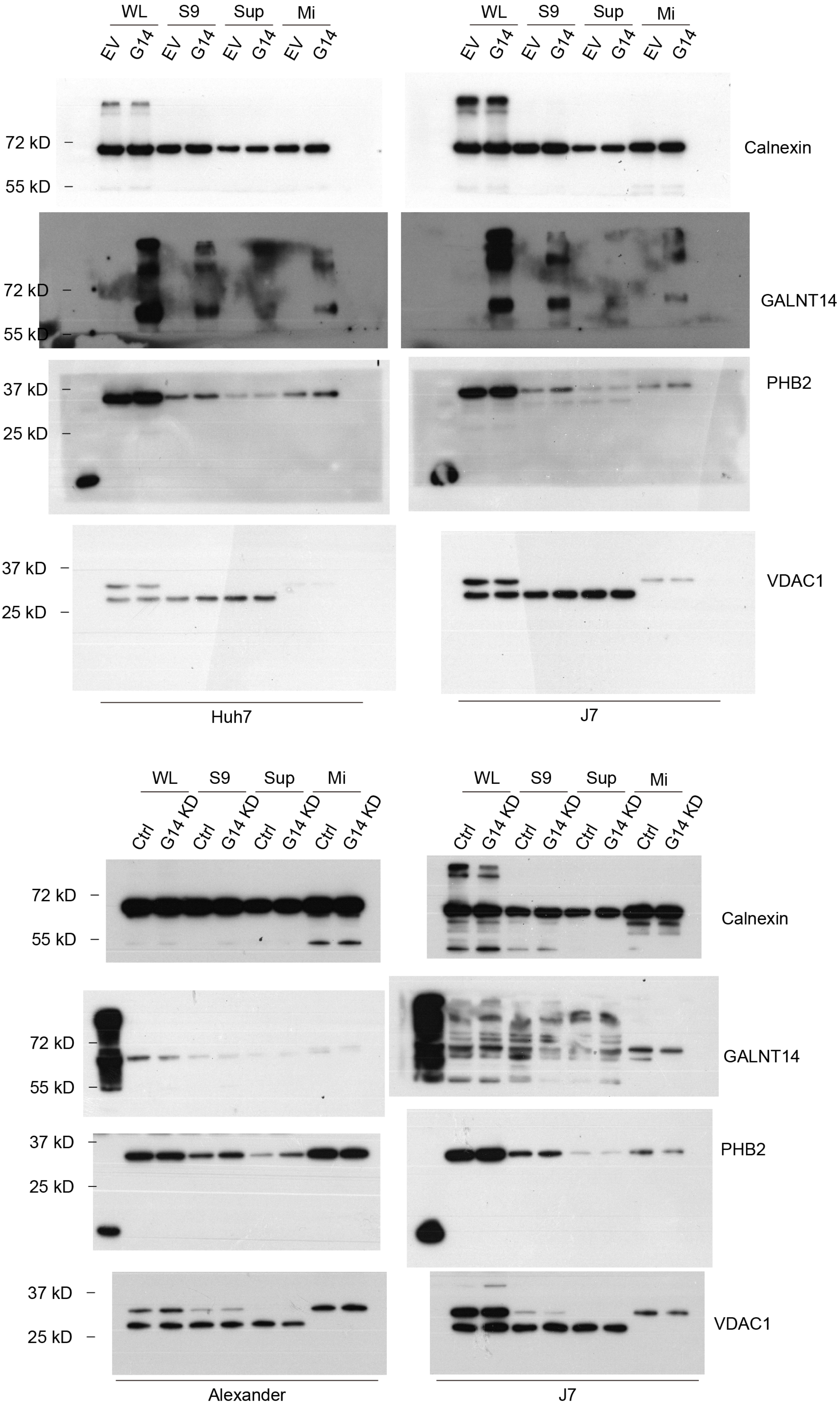

Original western blots for Fig 4B

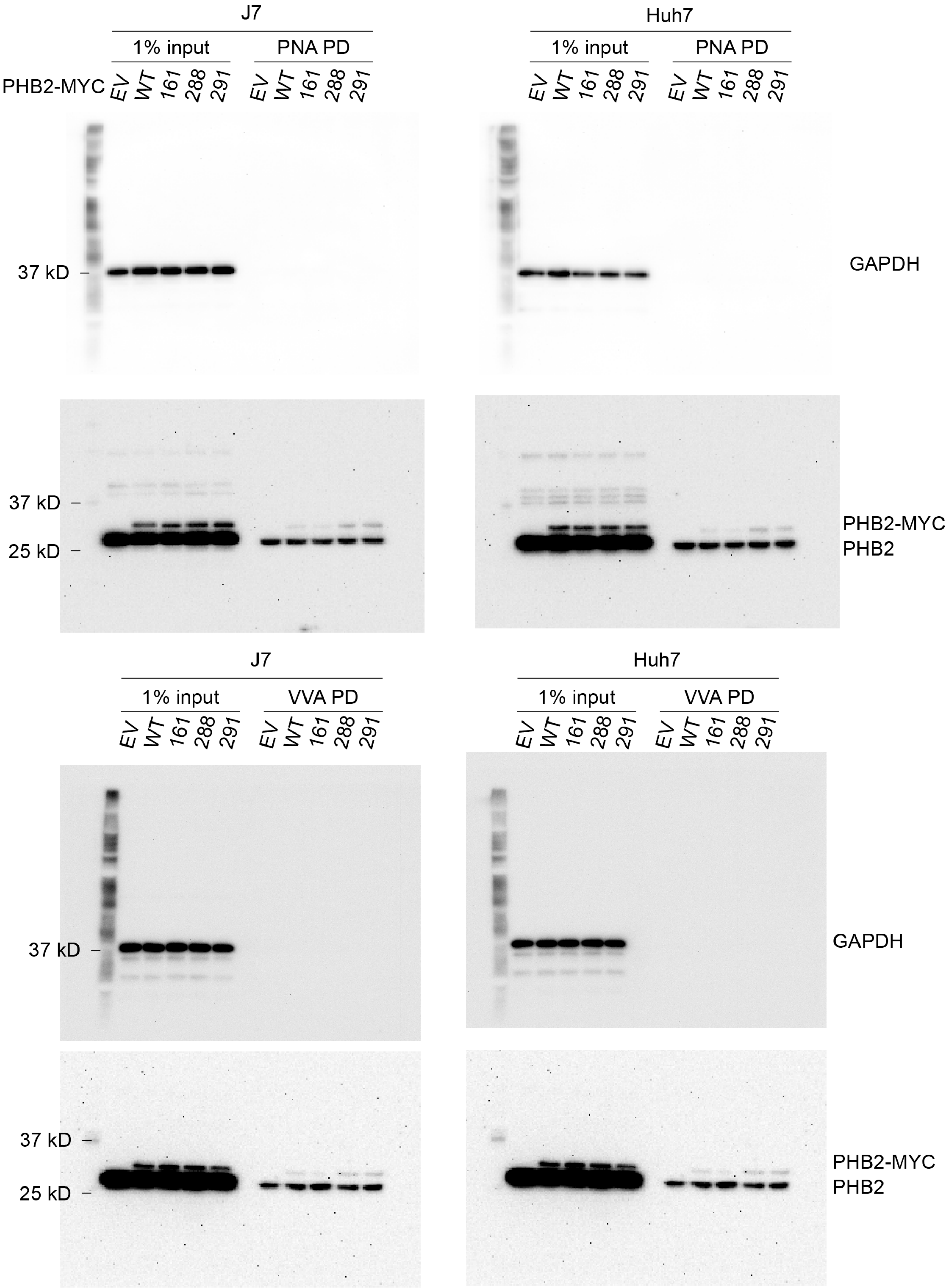

Original western blots for Fig 4D

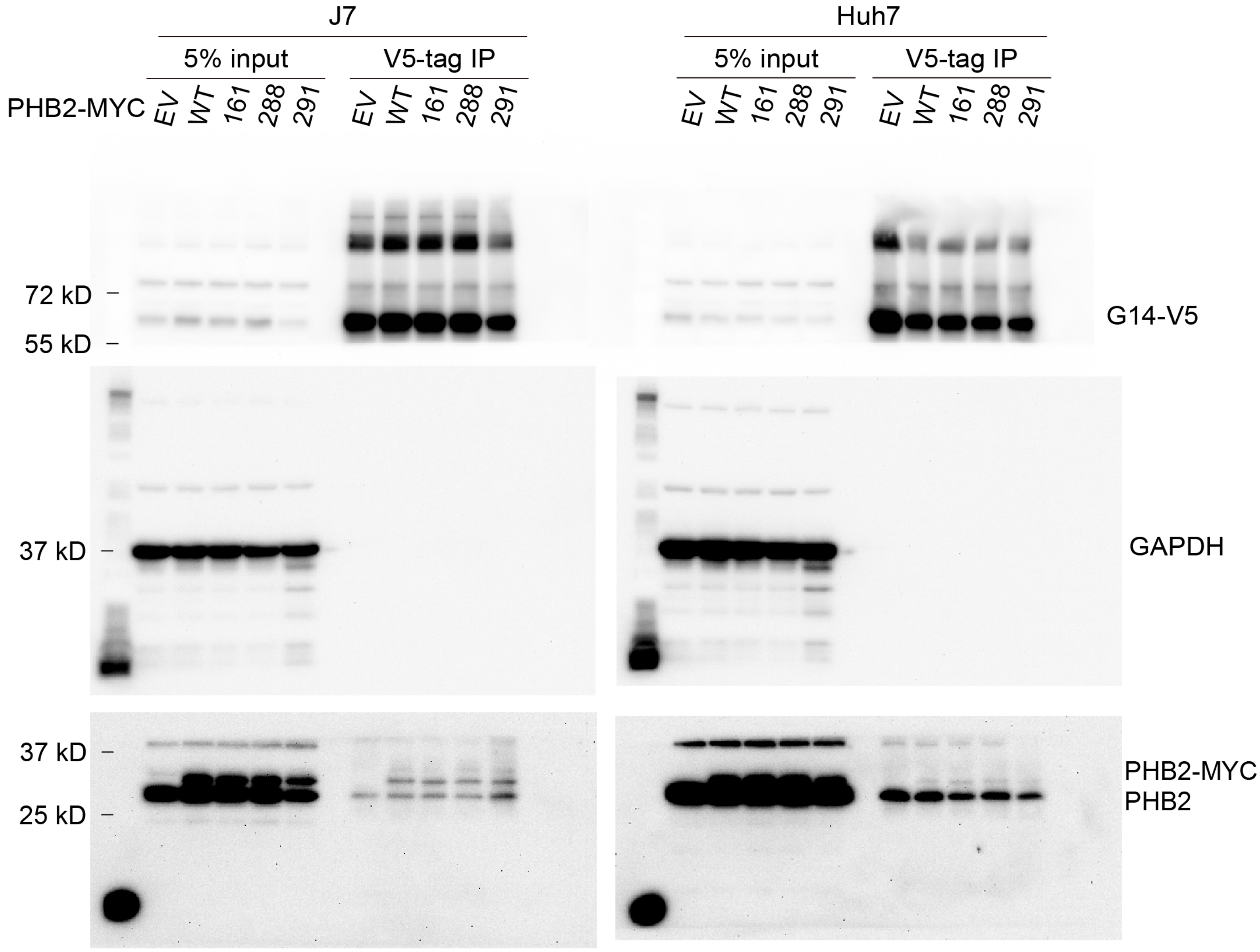

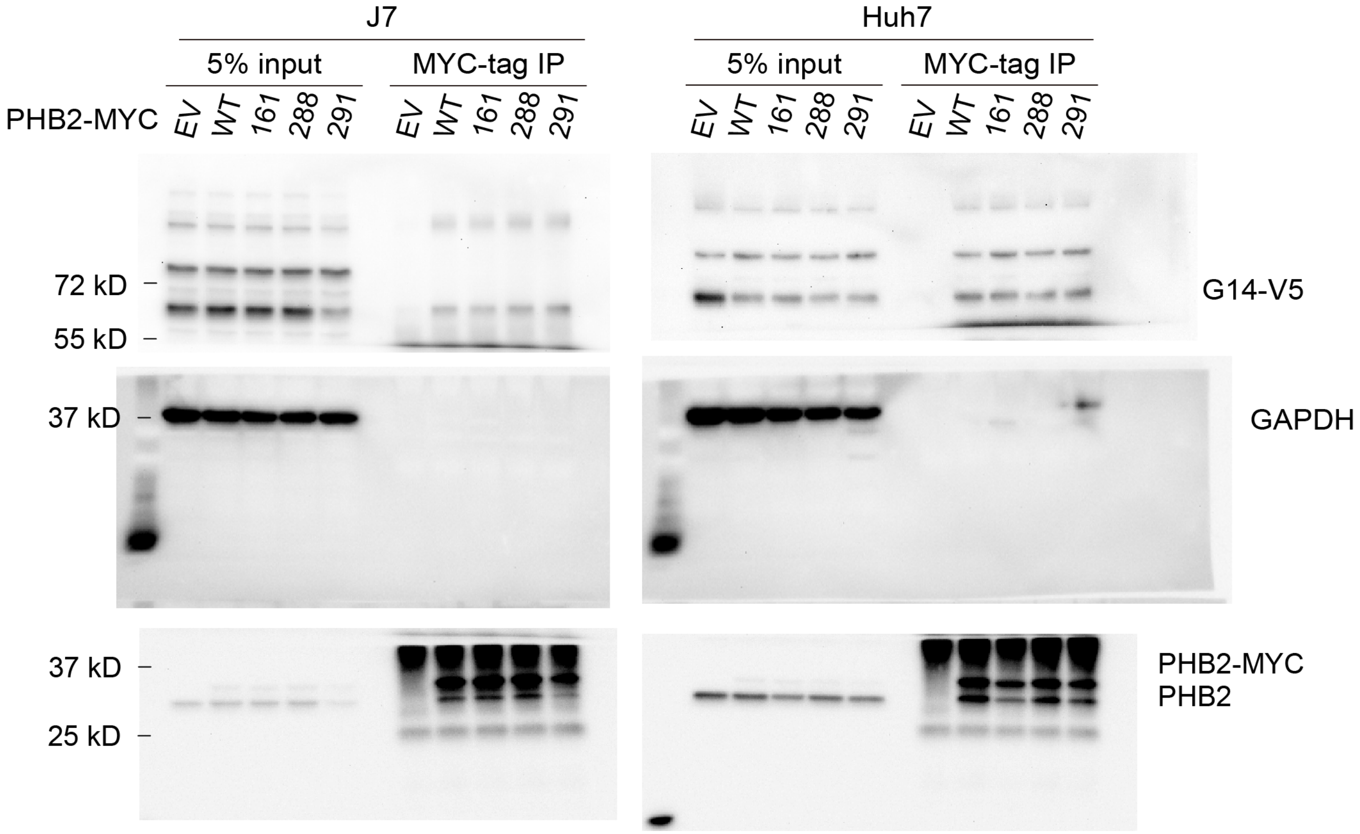

Original western blots for Fig 4E

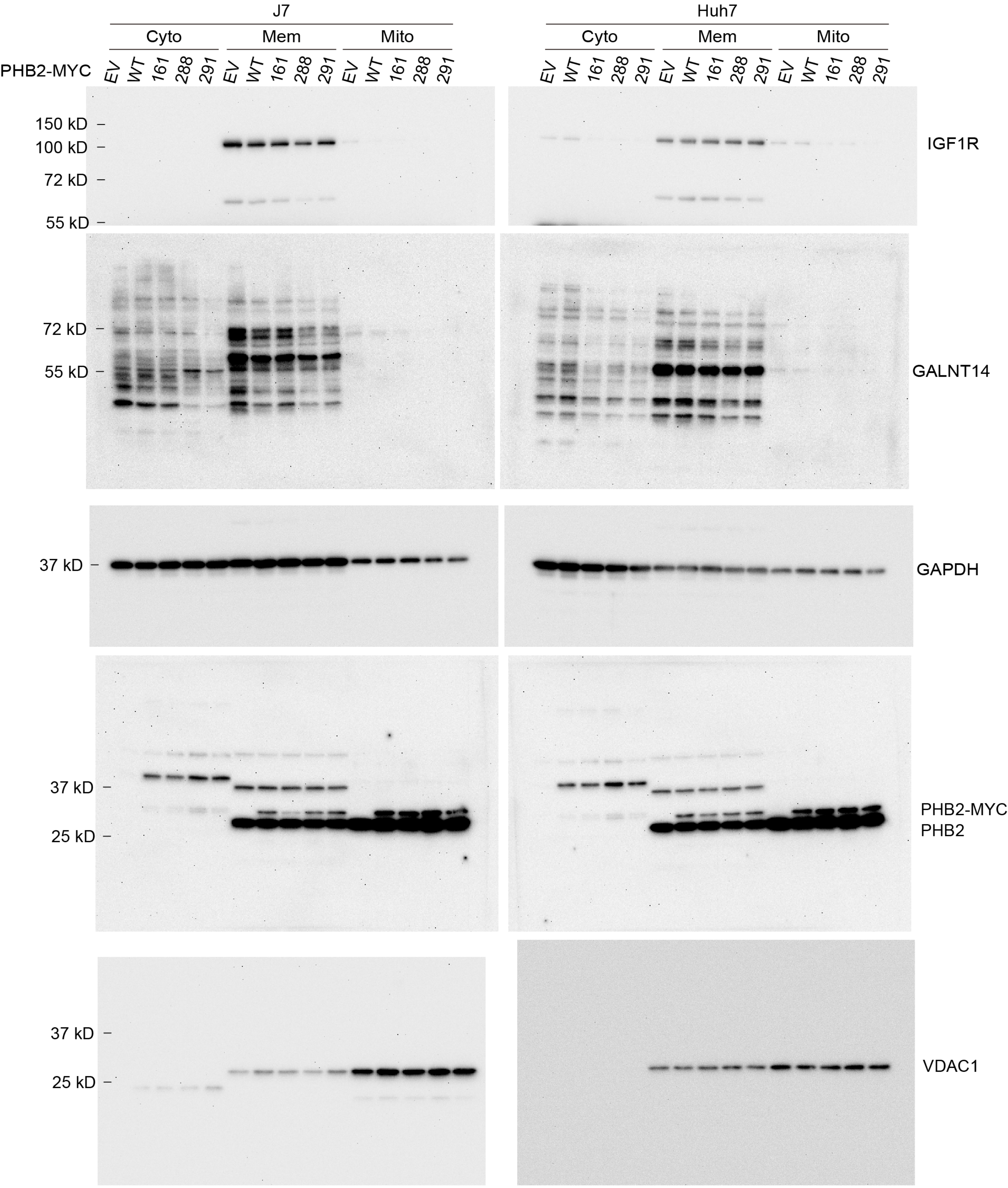

Original western blots for Fig 5A

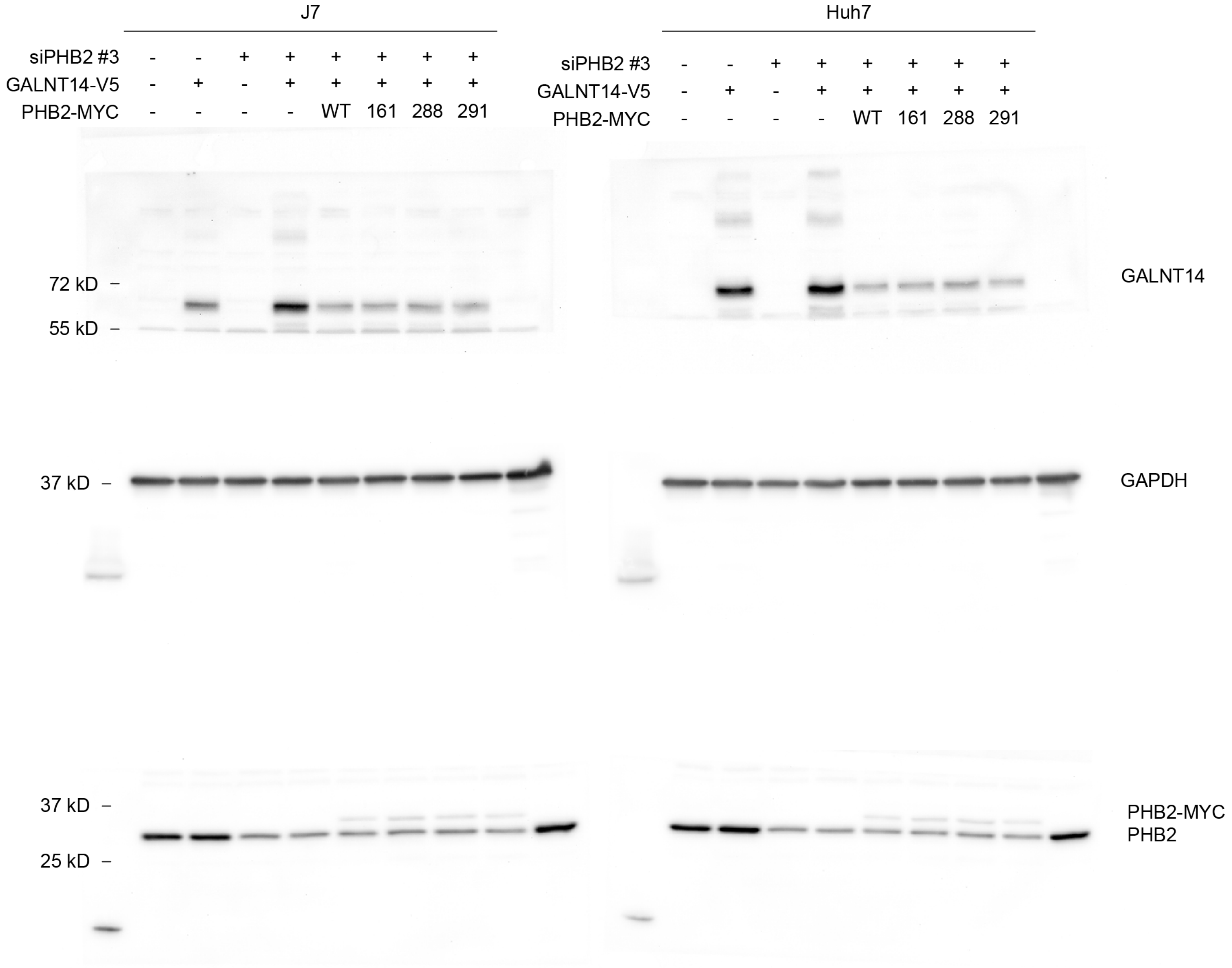

Original western blots for Fig 6A

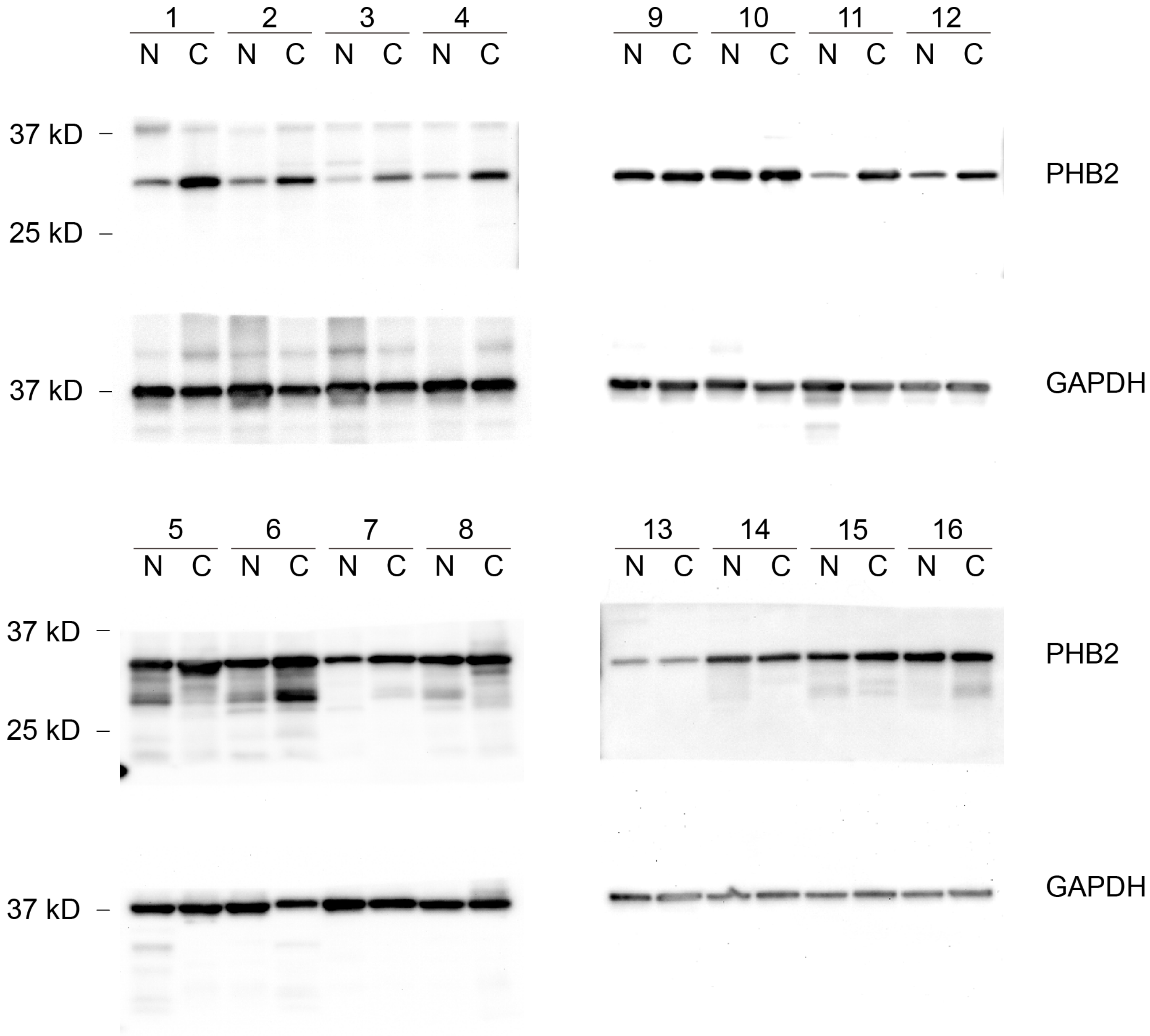

Original western blots for Fig 6E

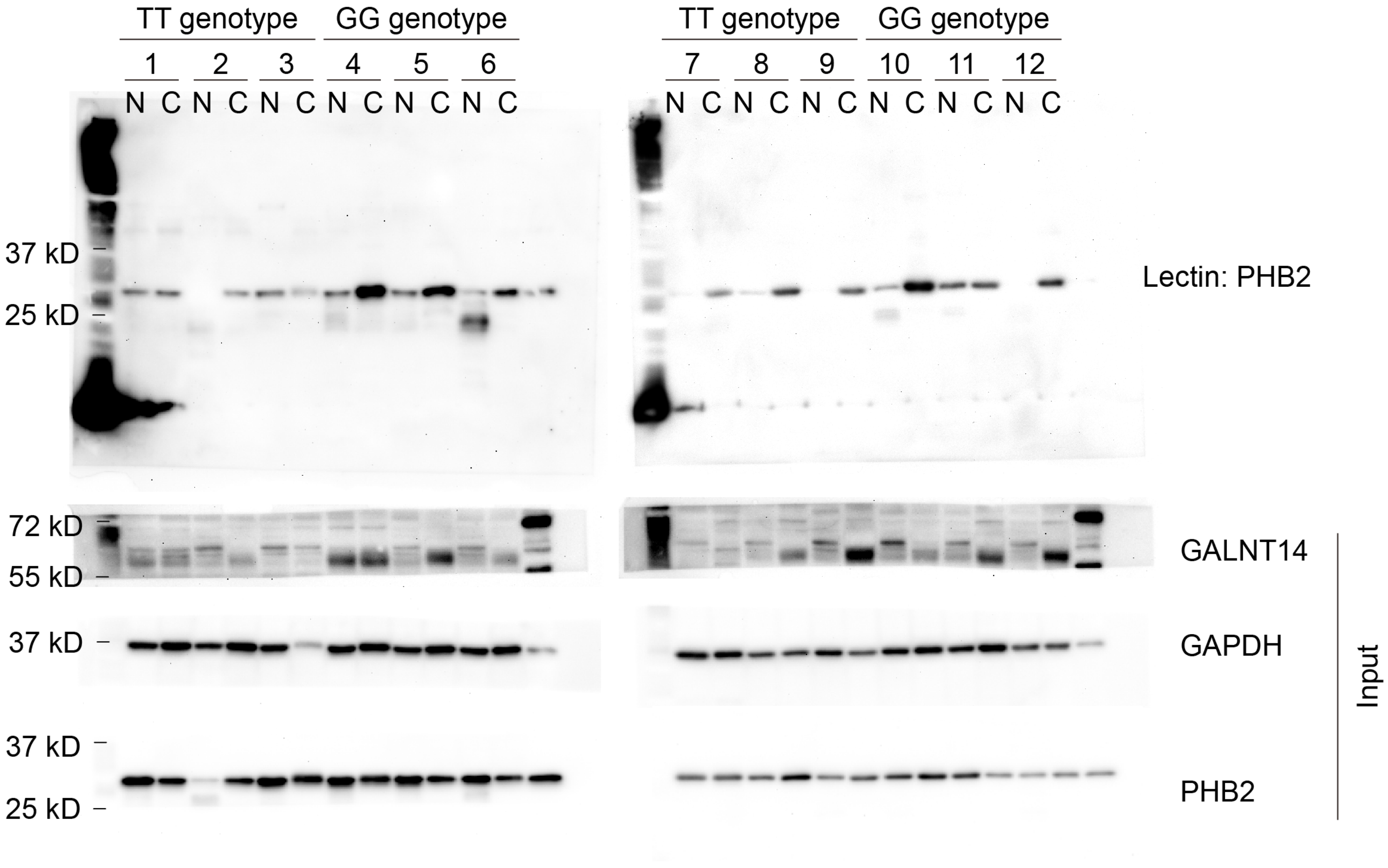

Original western blots for Fig 7A

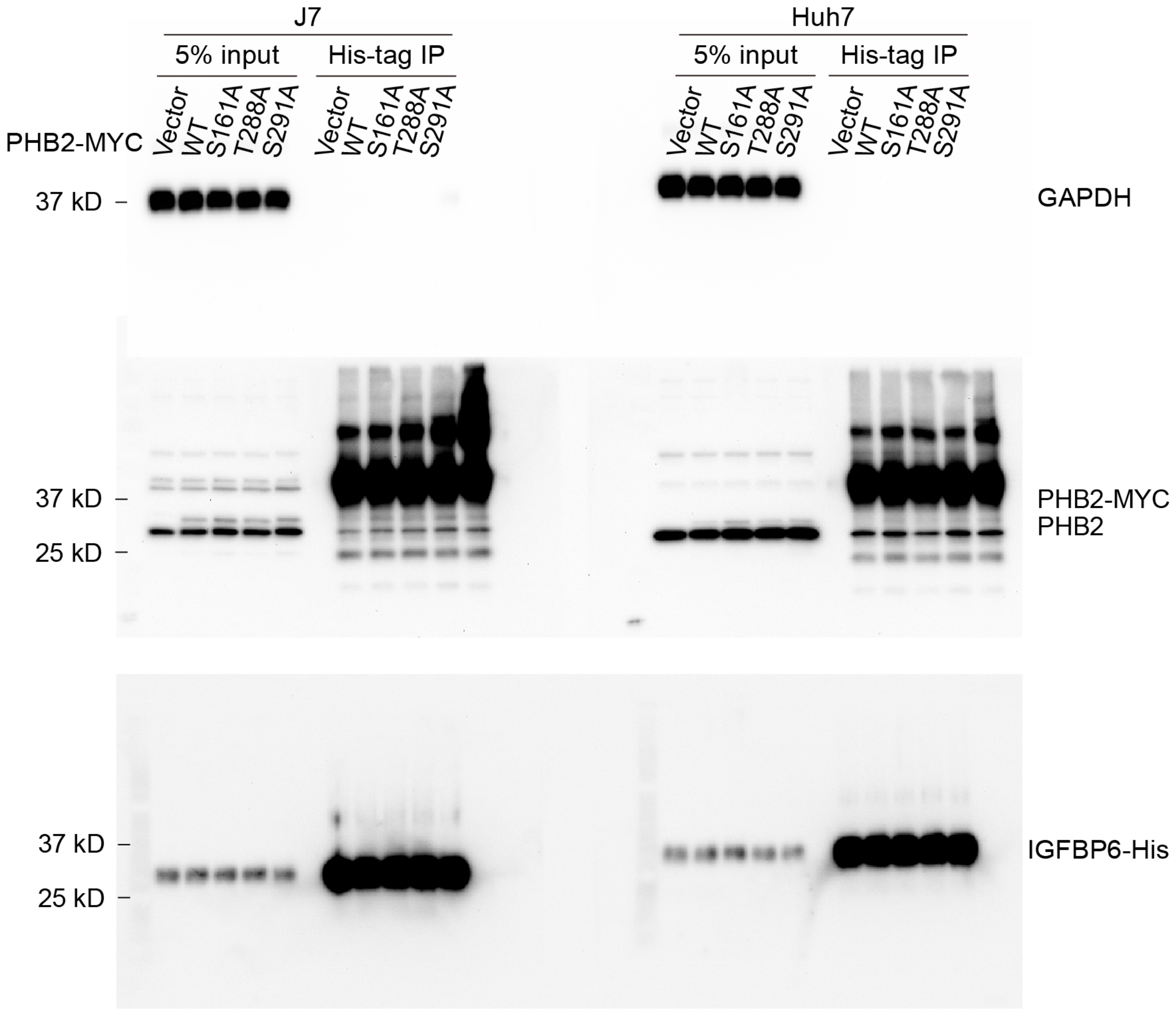

Original western blots for Fig 7B

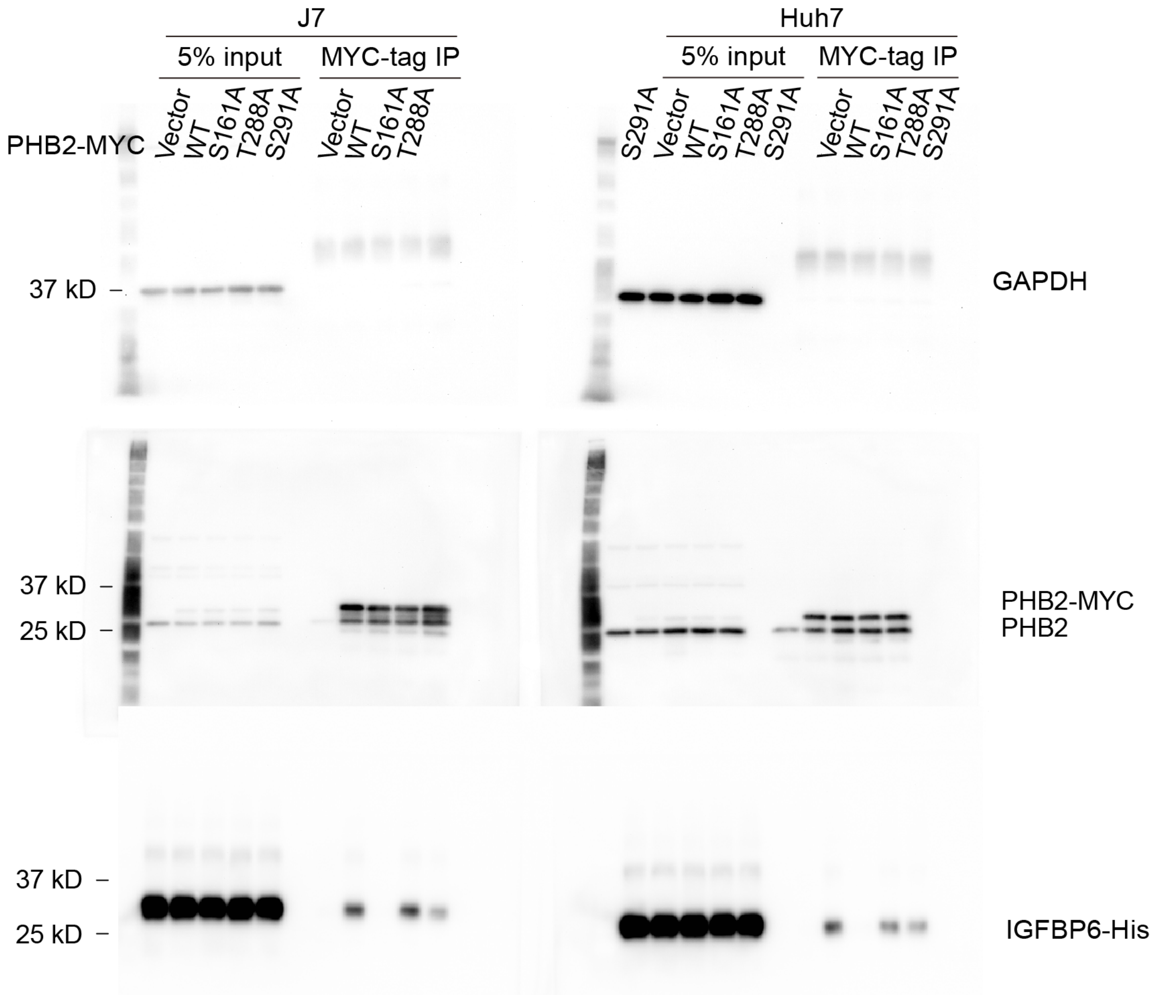

Original western blots for Fig 7C

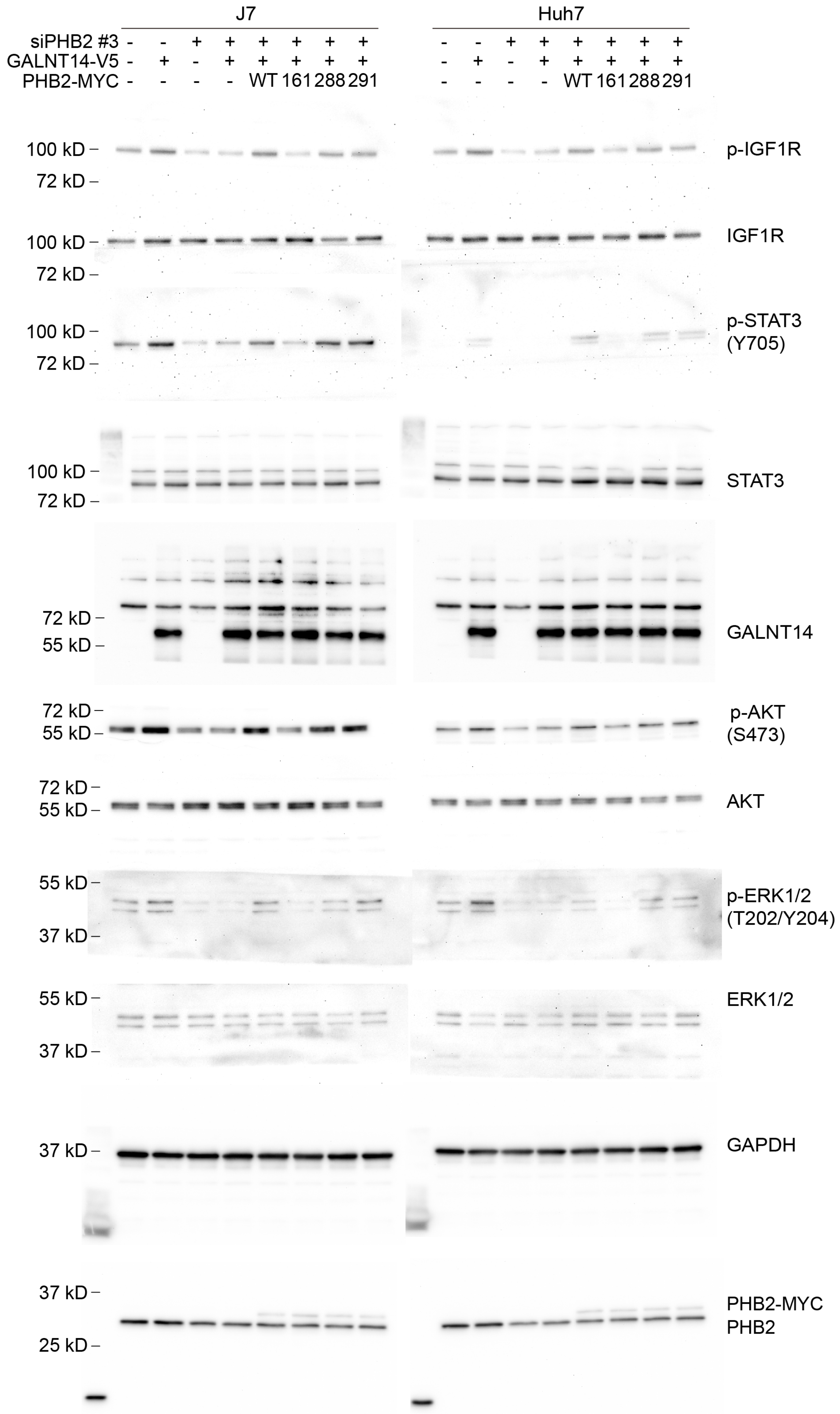

Supplement: Supplementary file 2 — Supplementary information [file 41419_2022_5419_MOESM2_ESM.pdf]
