# Supplementary material for: GALNT14-mediated O-glycosylation on PHB2 serine-161 enhances cell growth, migration and drug resistance by activating IGF1R cascade in hepatoma cells
Source: Cell Death Dis. 2022 Nov 14;13(11):956. doi: 10.1038/s41419-022-05419-y (PMC9663550; doi:10.1038/s41419-022-05419-y)

Original western blots for Fig 1C

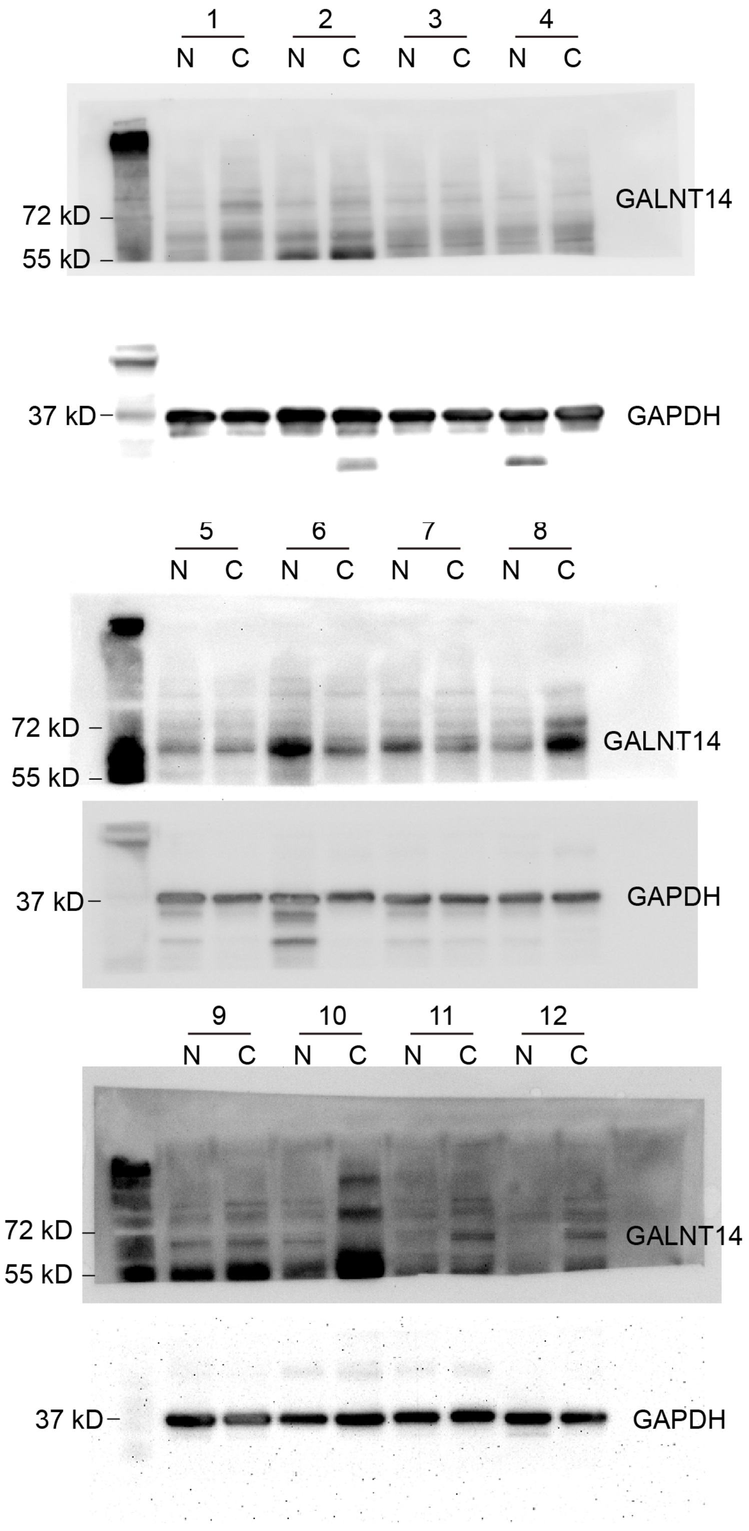

Original western blots for Fig 2A

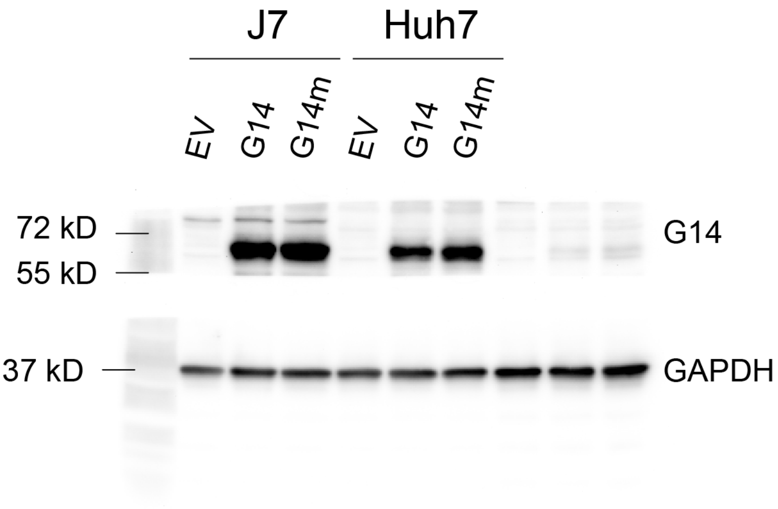

Original western blots for Fig 2C

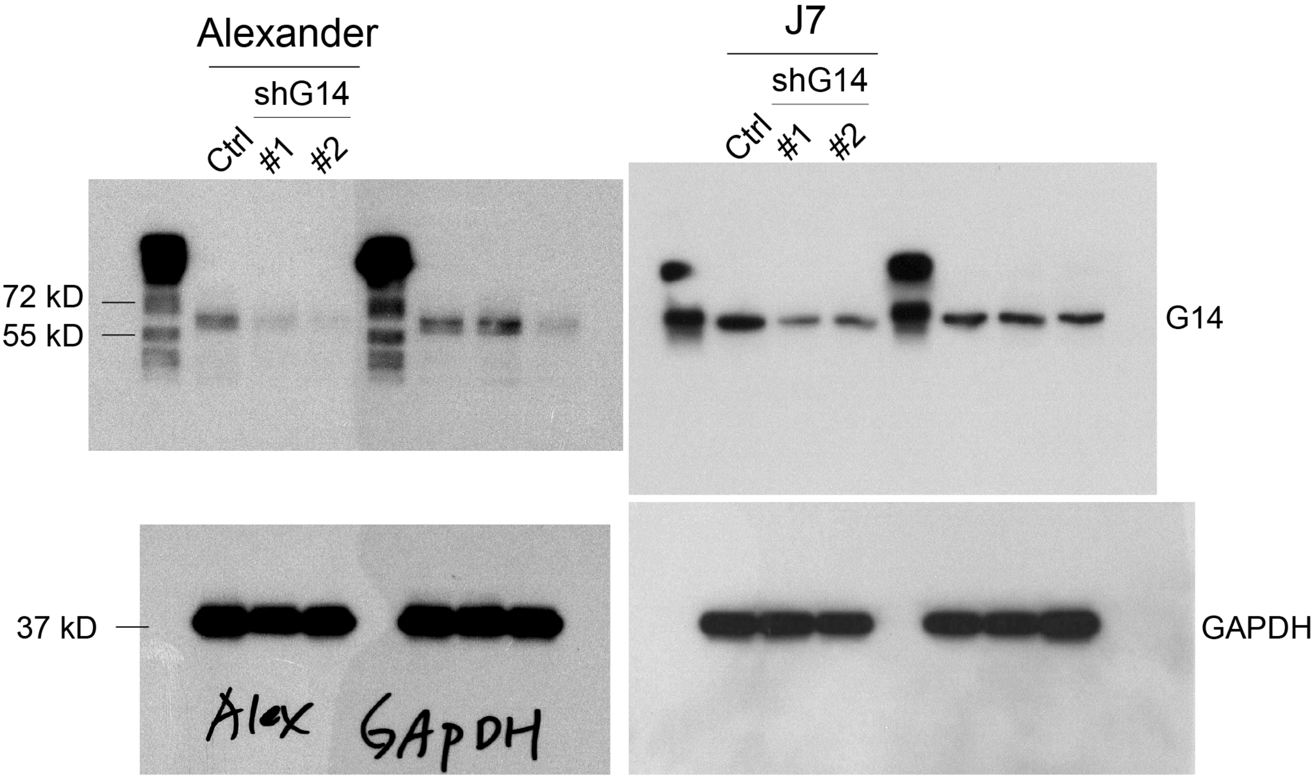

Original western blots for Fig 3B

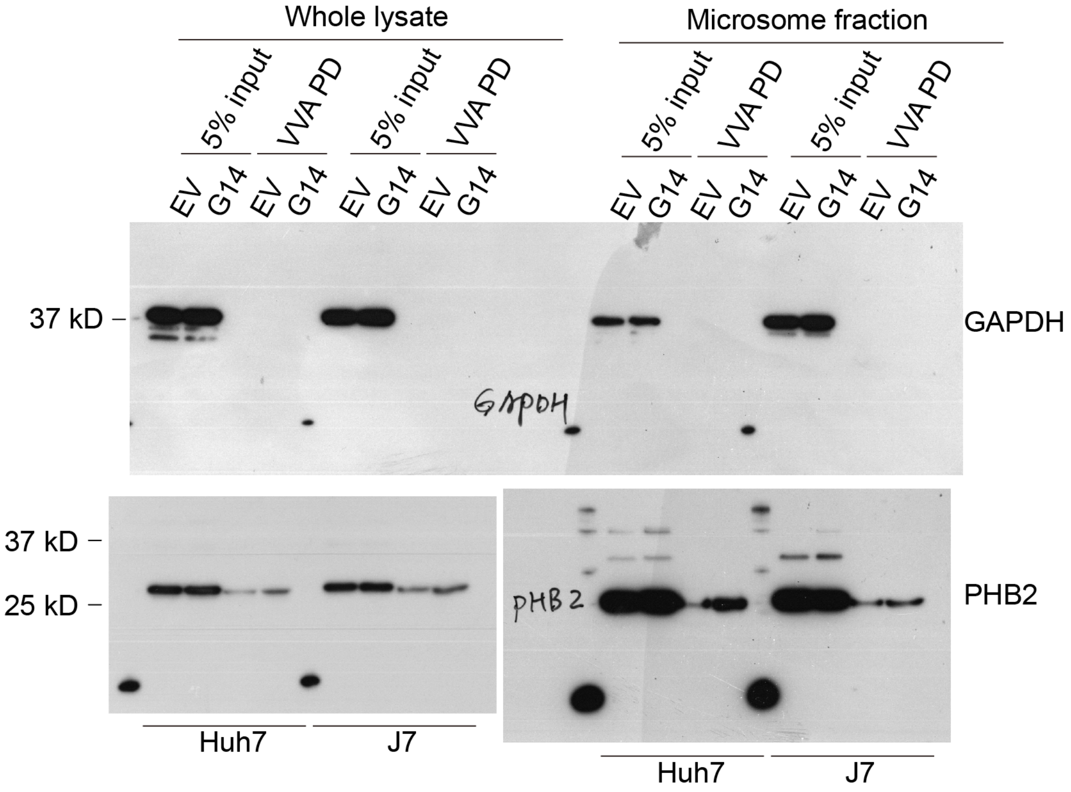

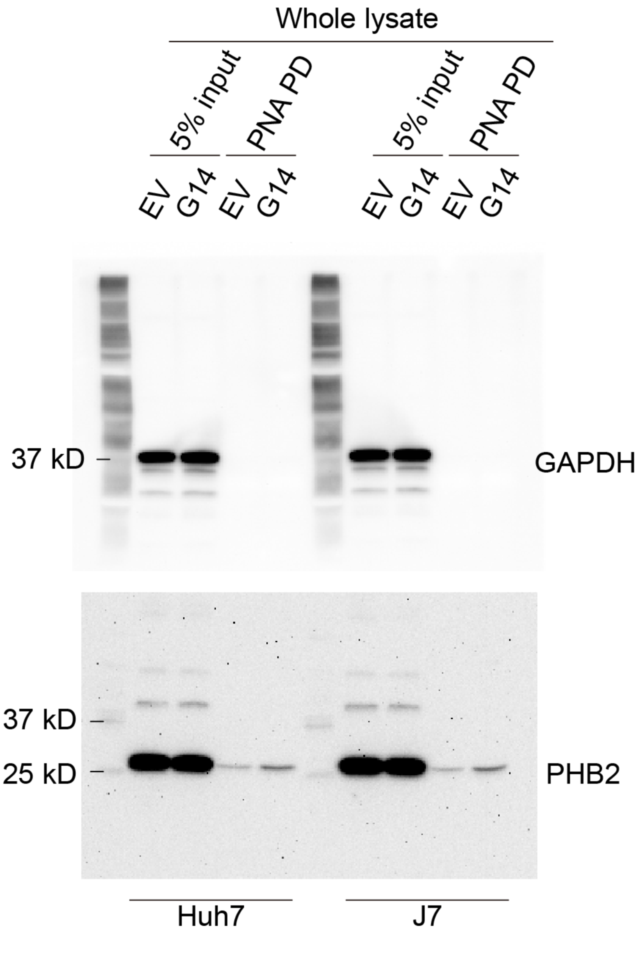

Original western blots for Fig 3C

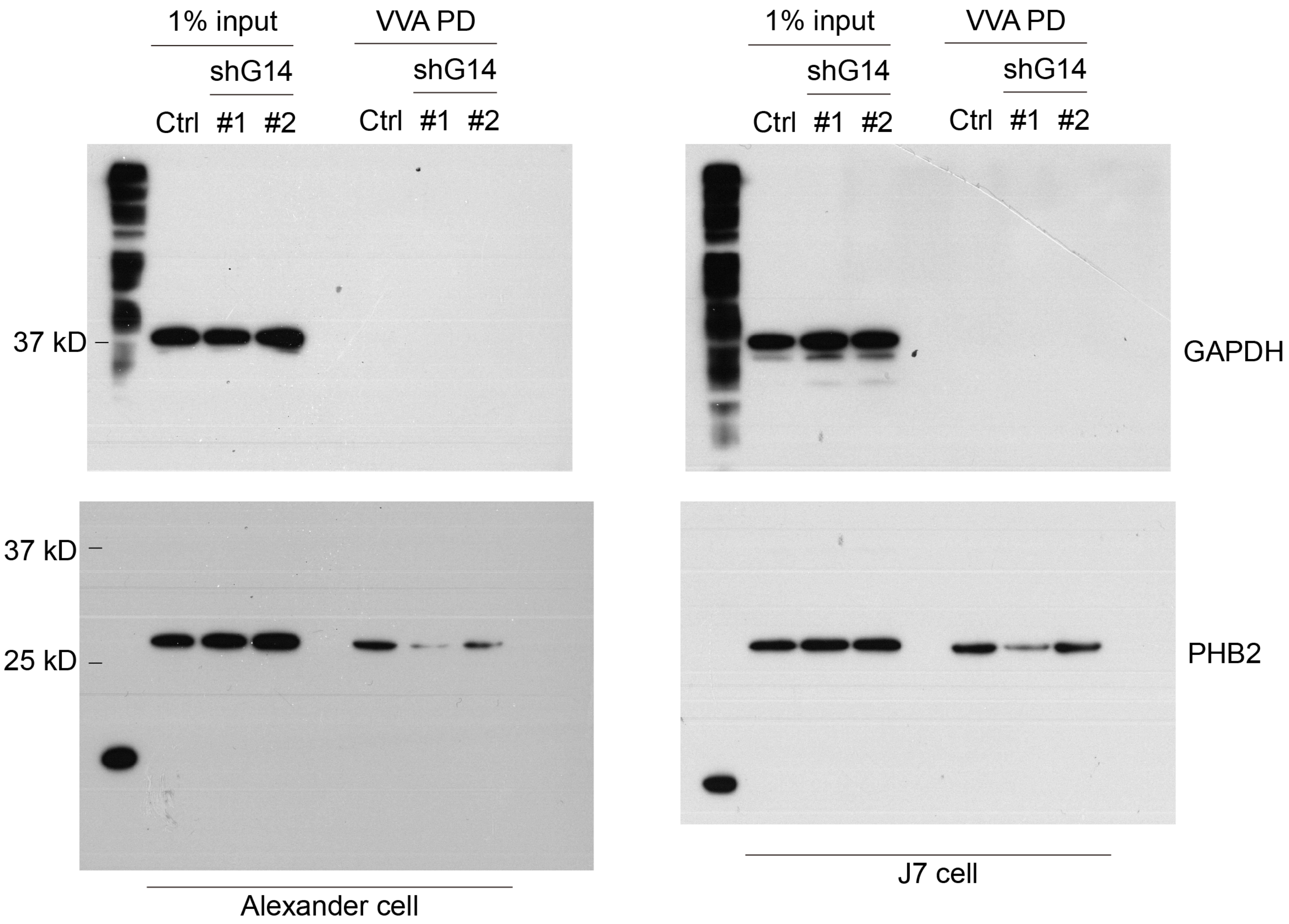

Original western blots for Fig 3D

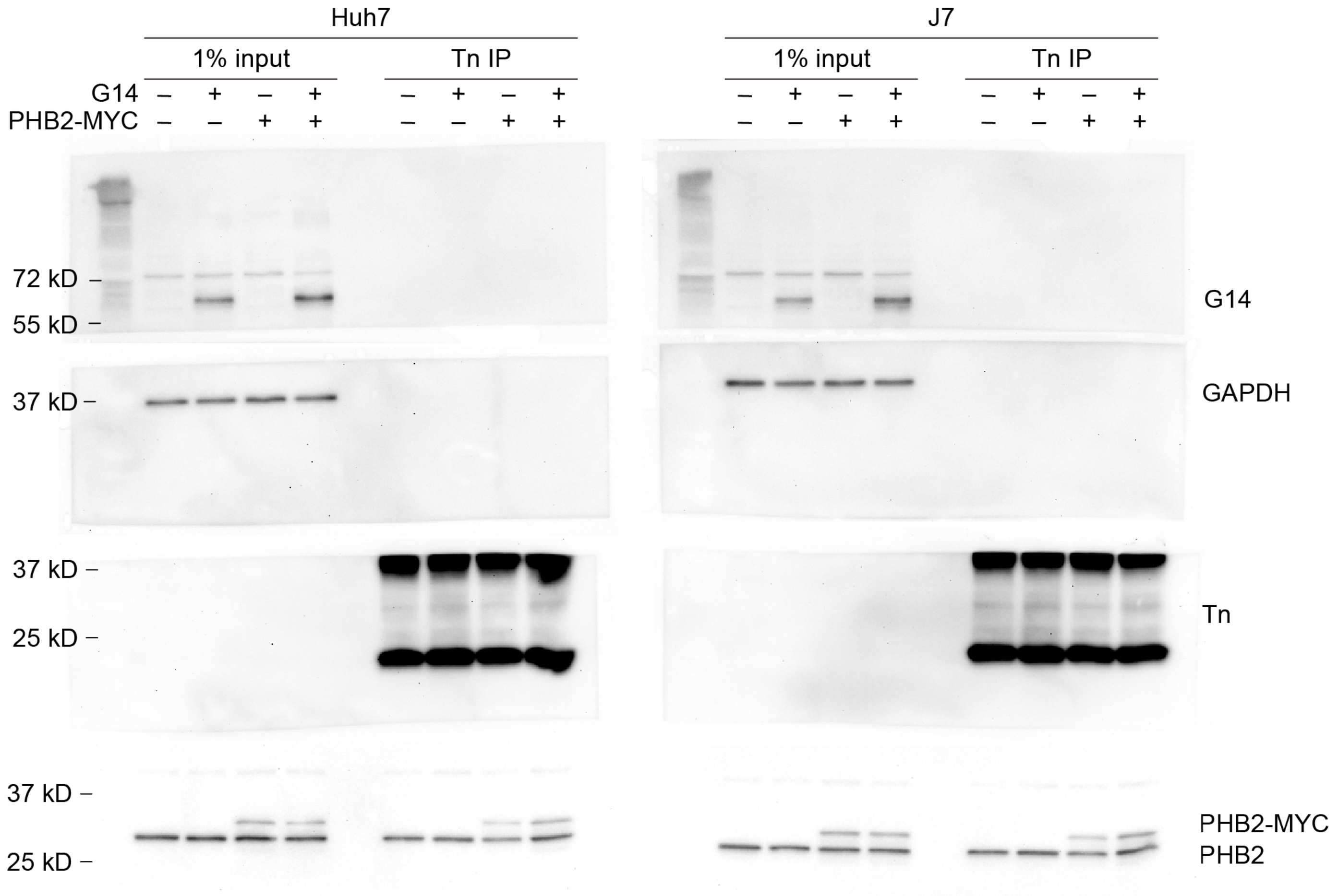

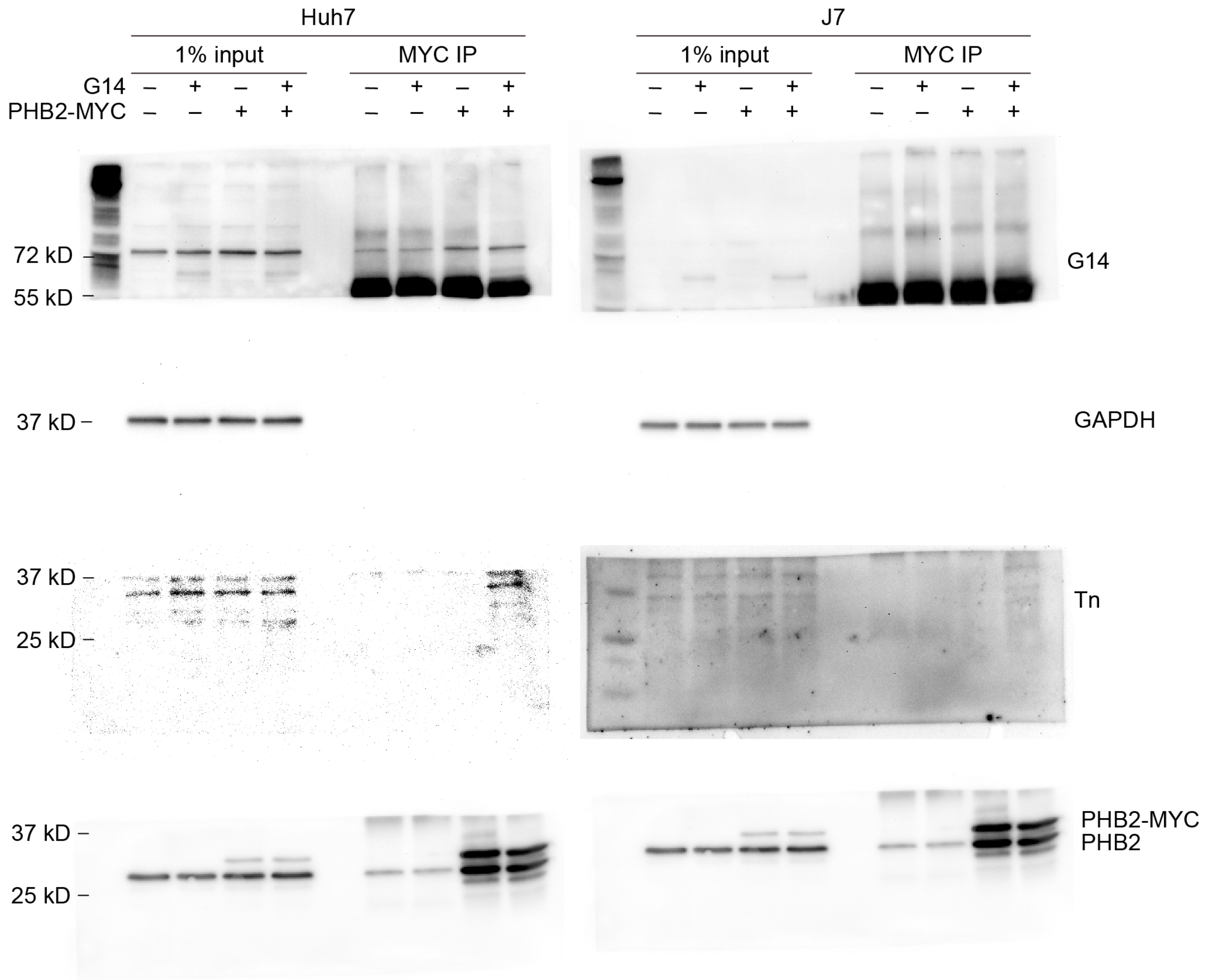

Original western blots for Fig 3E

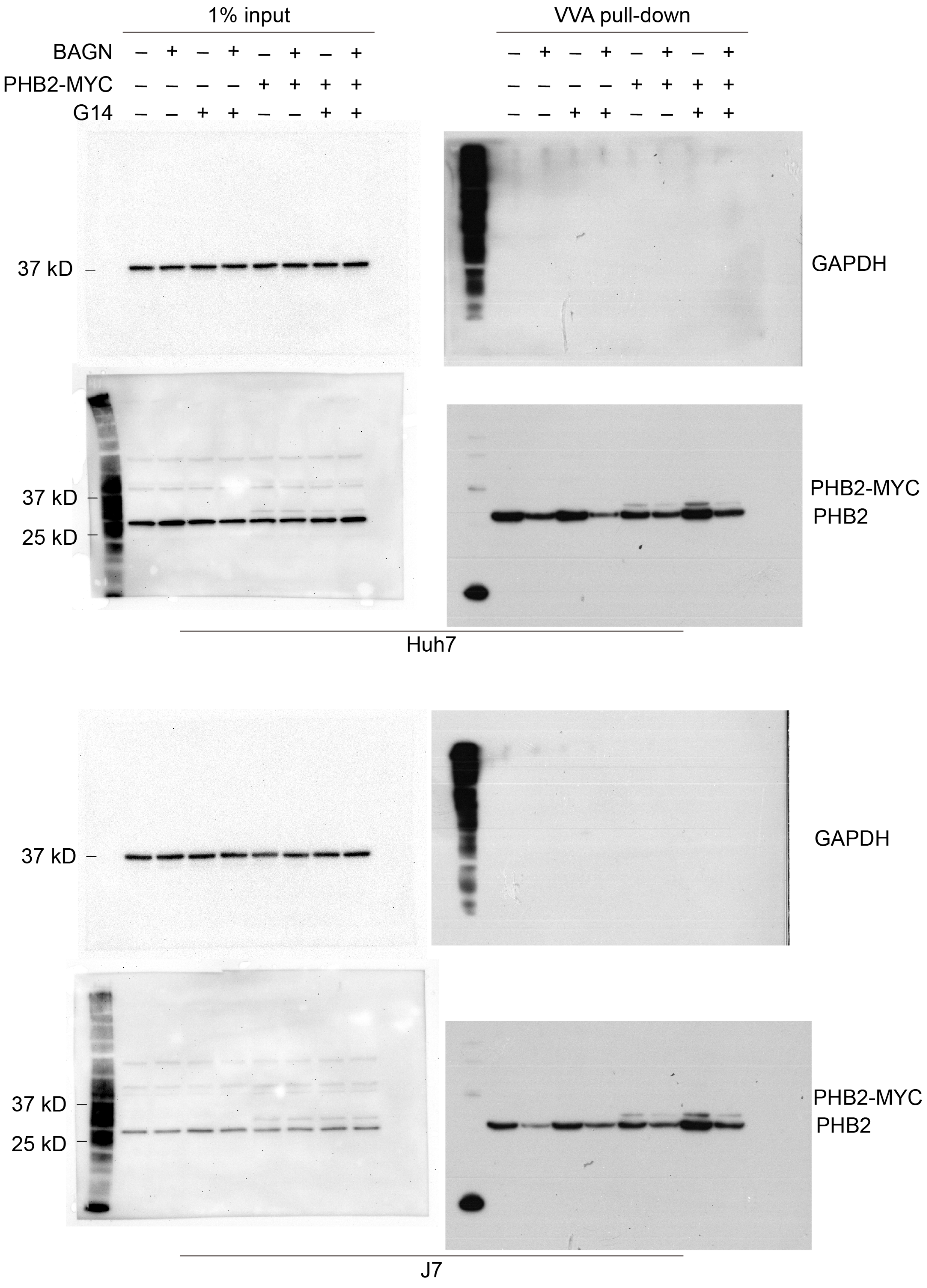

Original western blots for Fig 3F

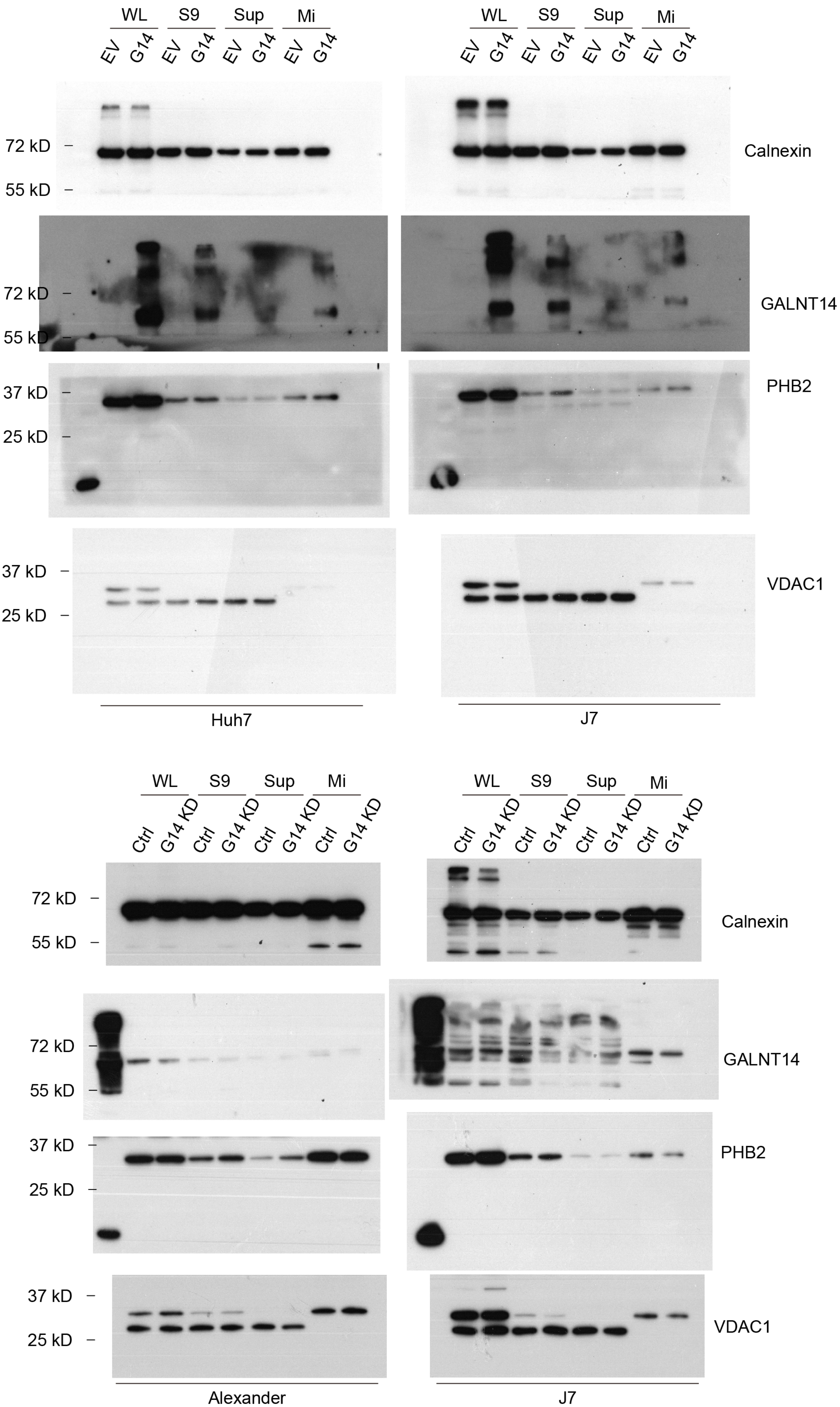

Original western blots for Fig 4B

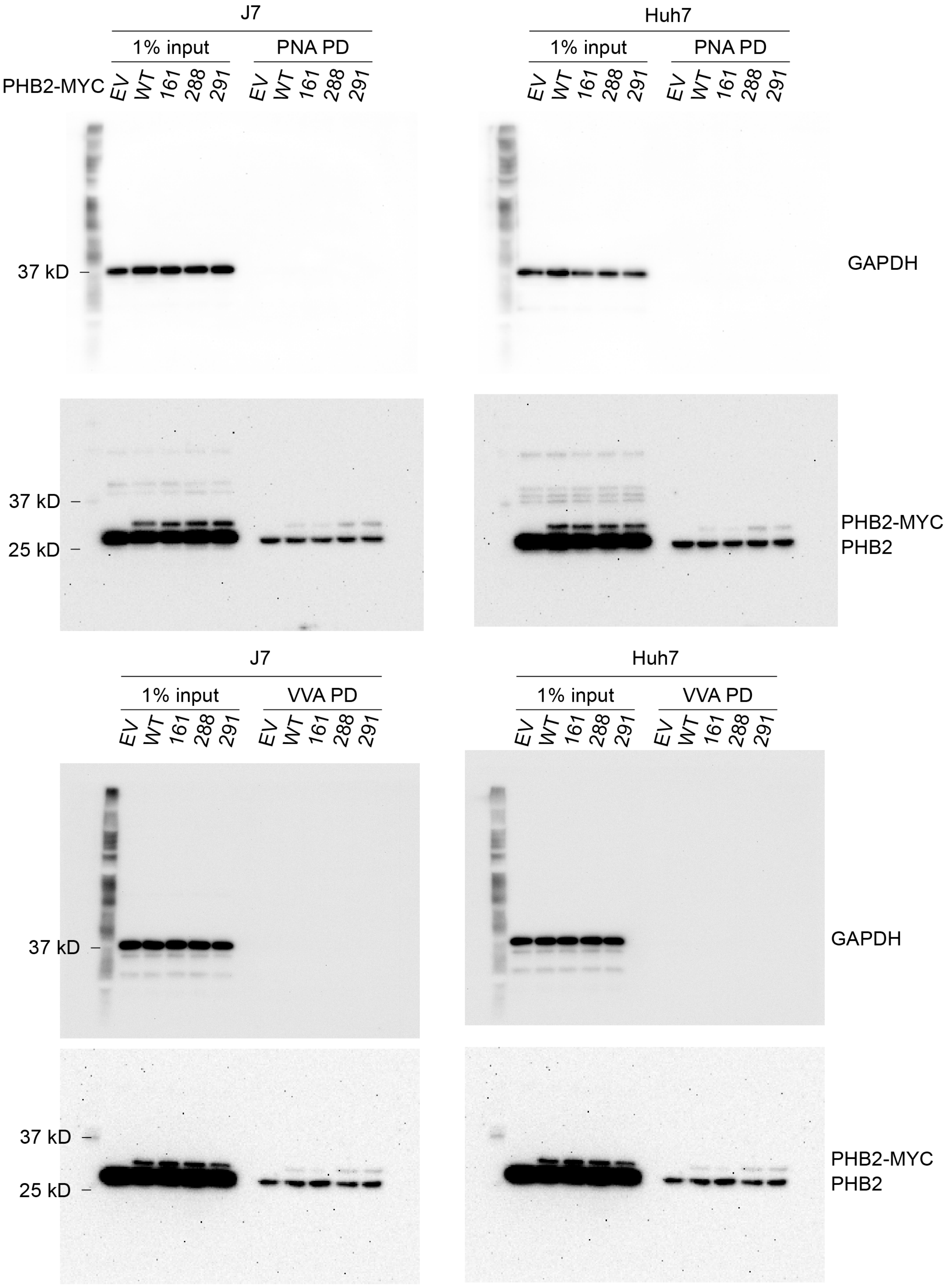

Original western blots for Fig 4D

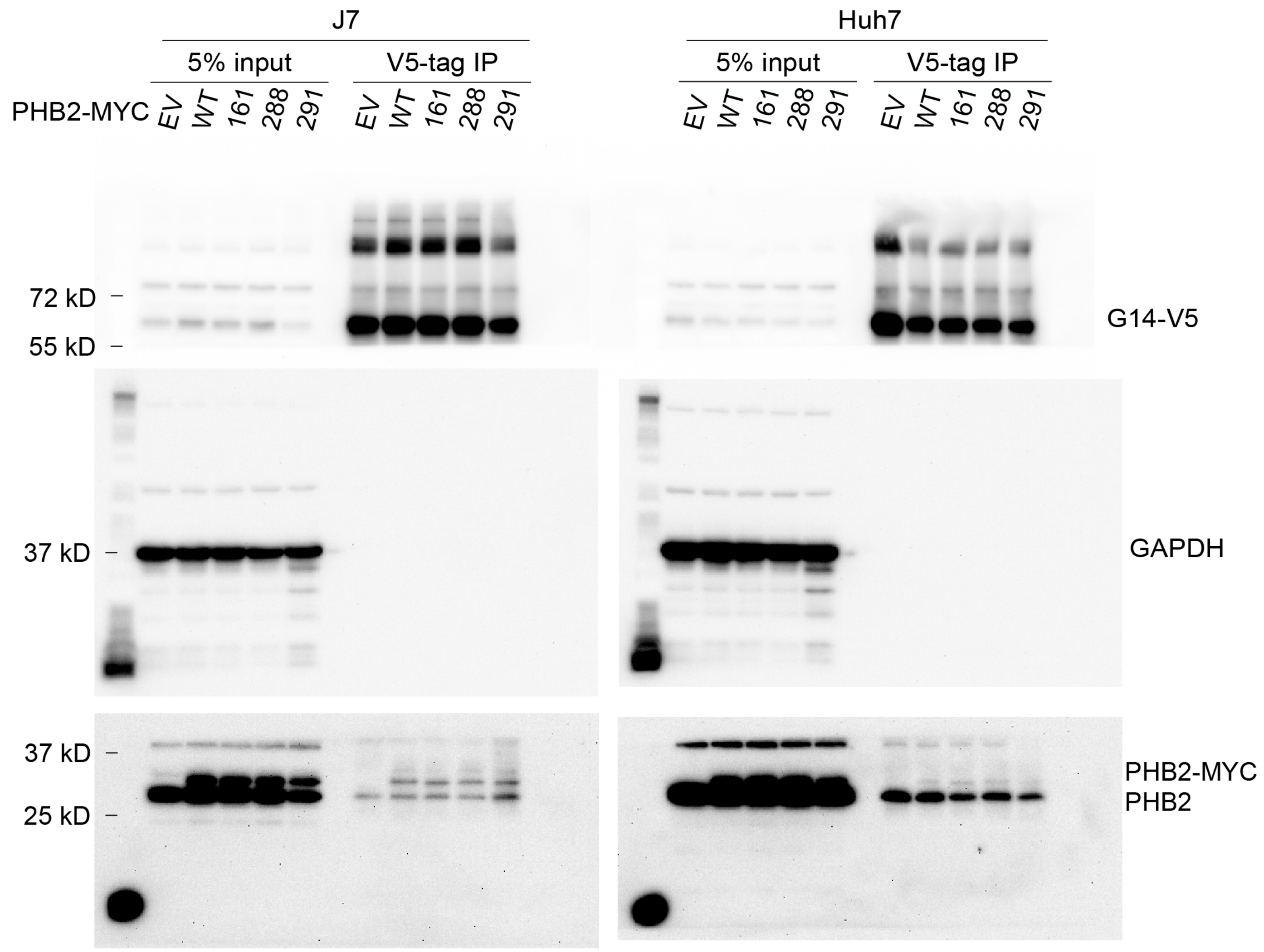

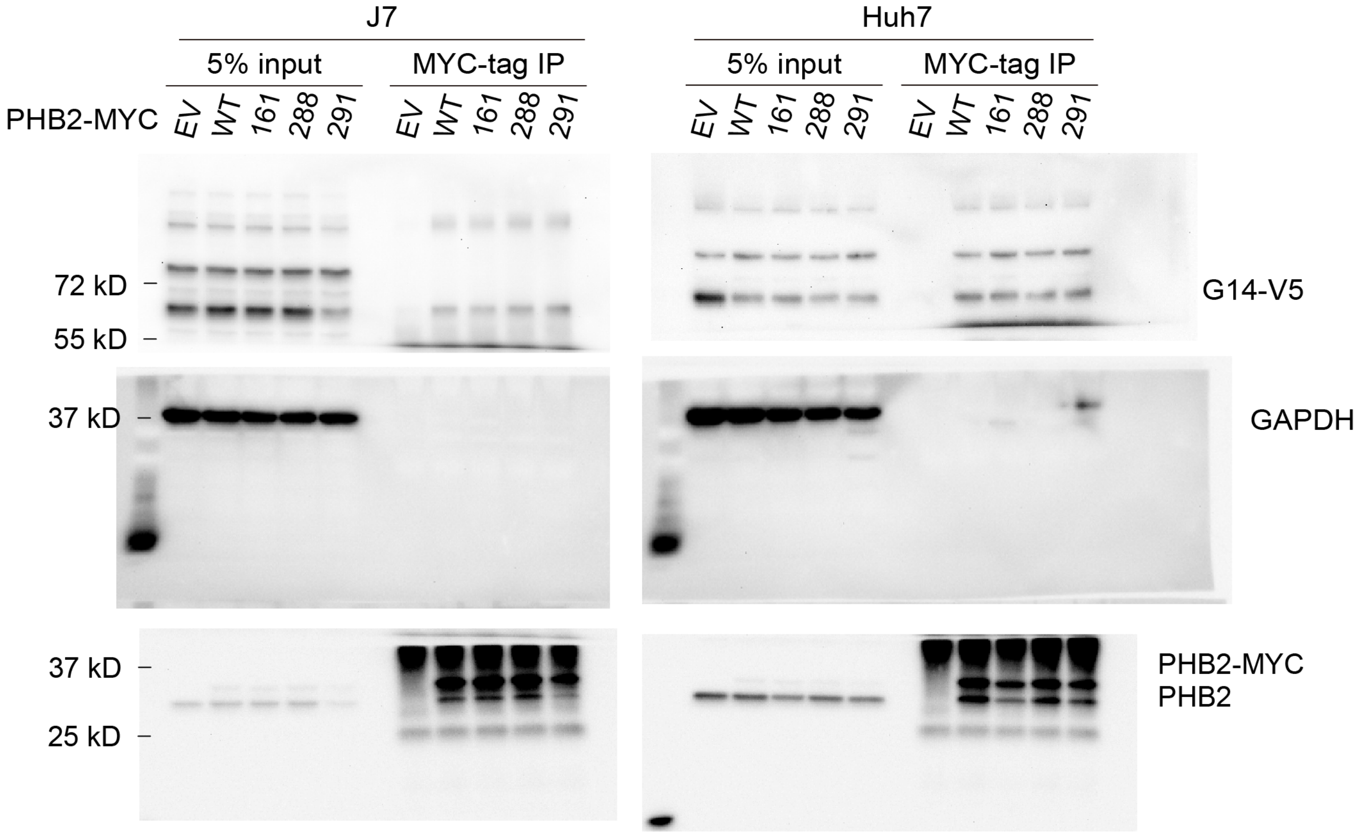

Original western blots for Fig 4E

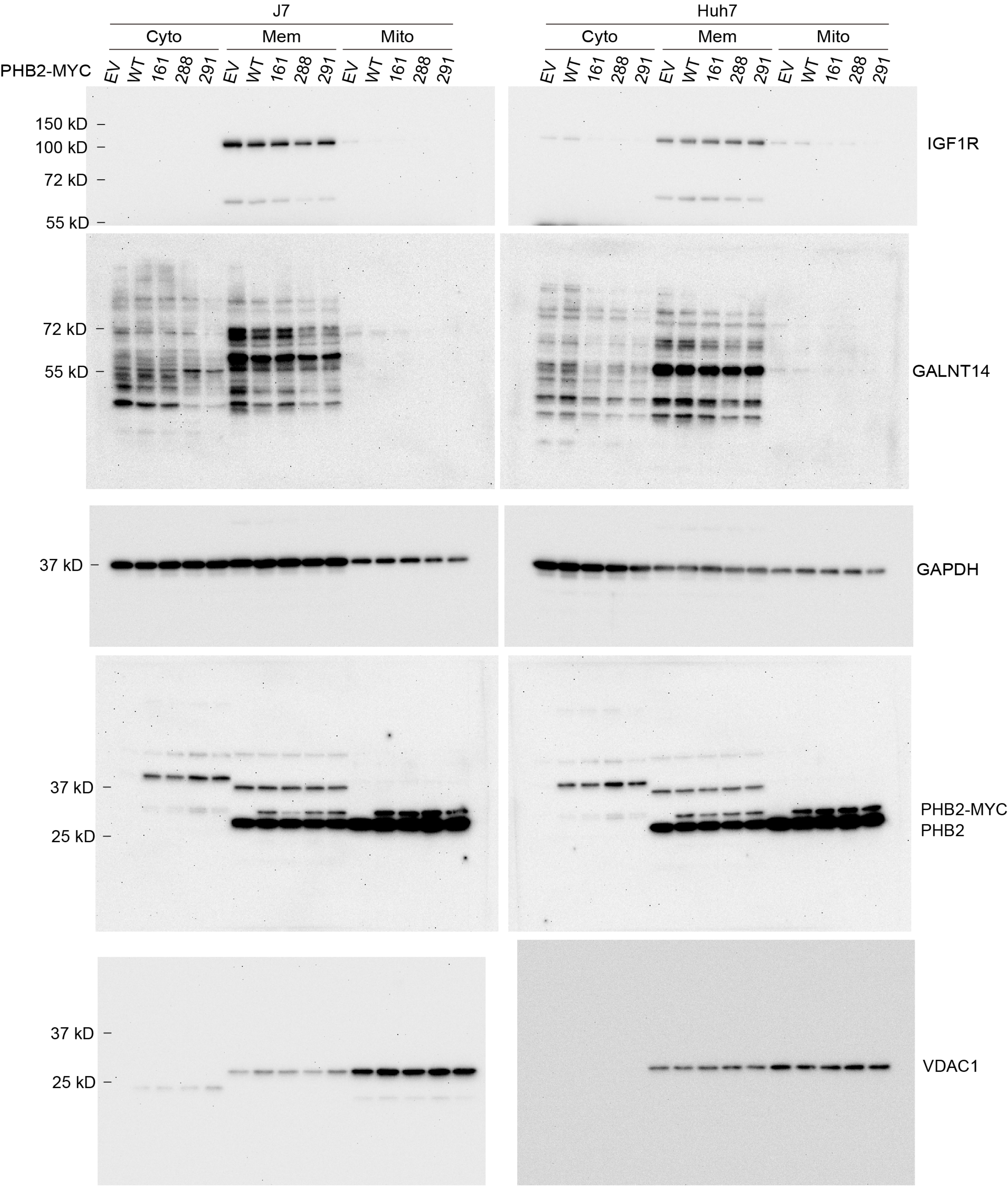

Original western blots for Fig 5A

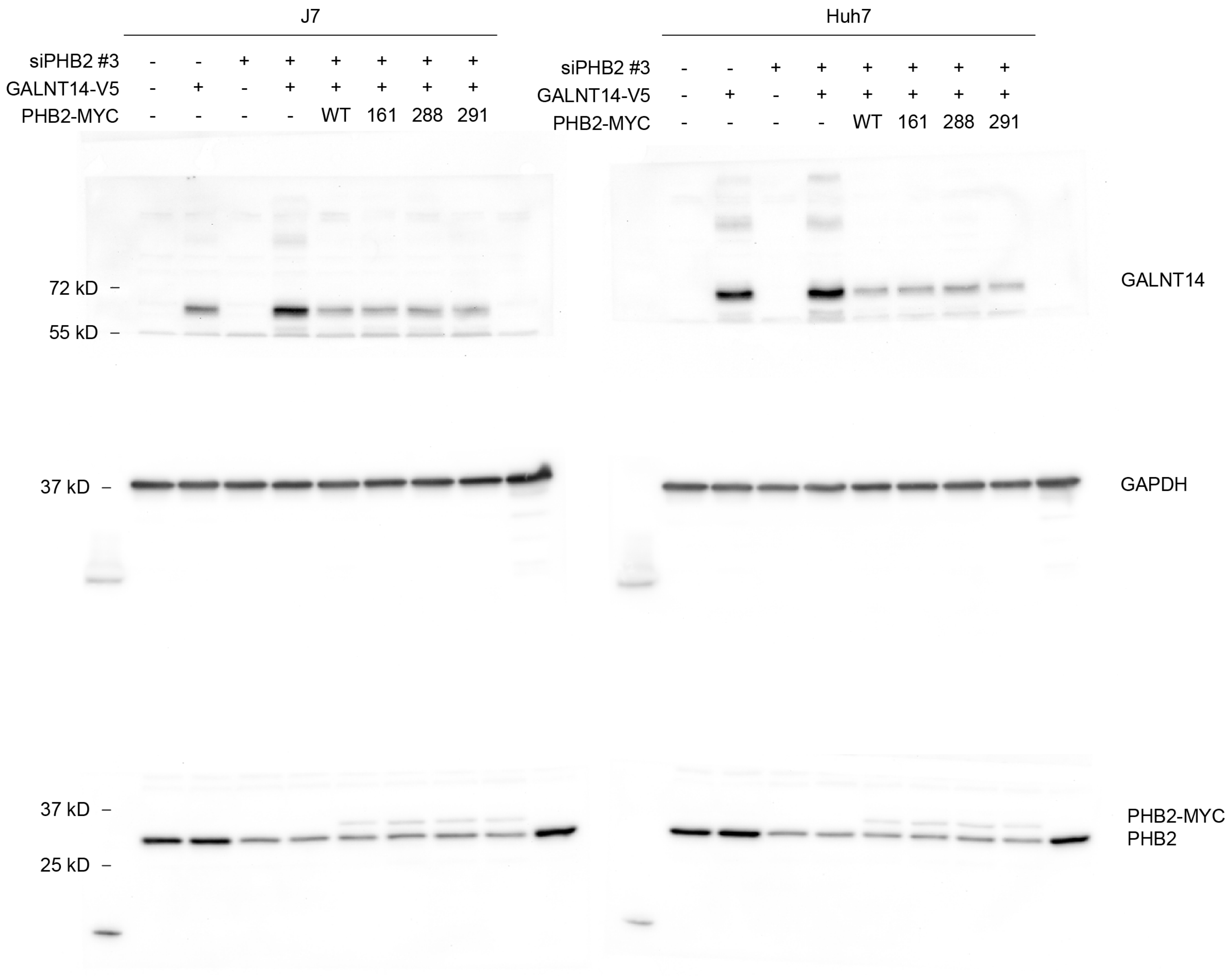

Original western blots for Fig 6A

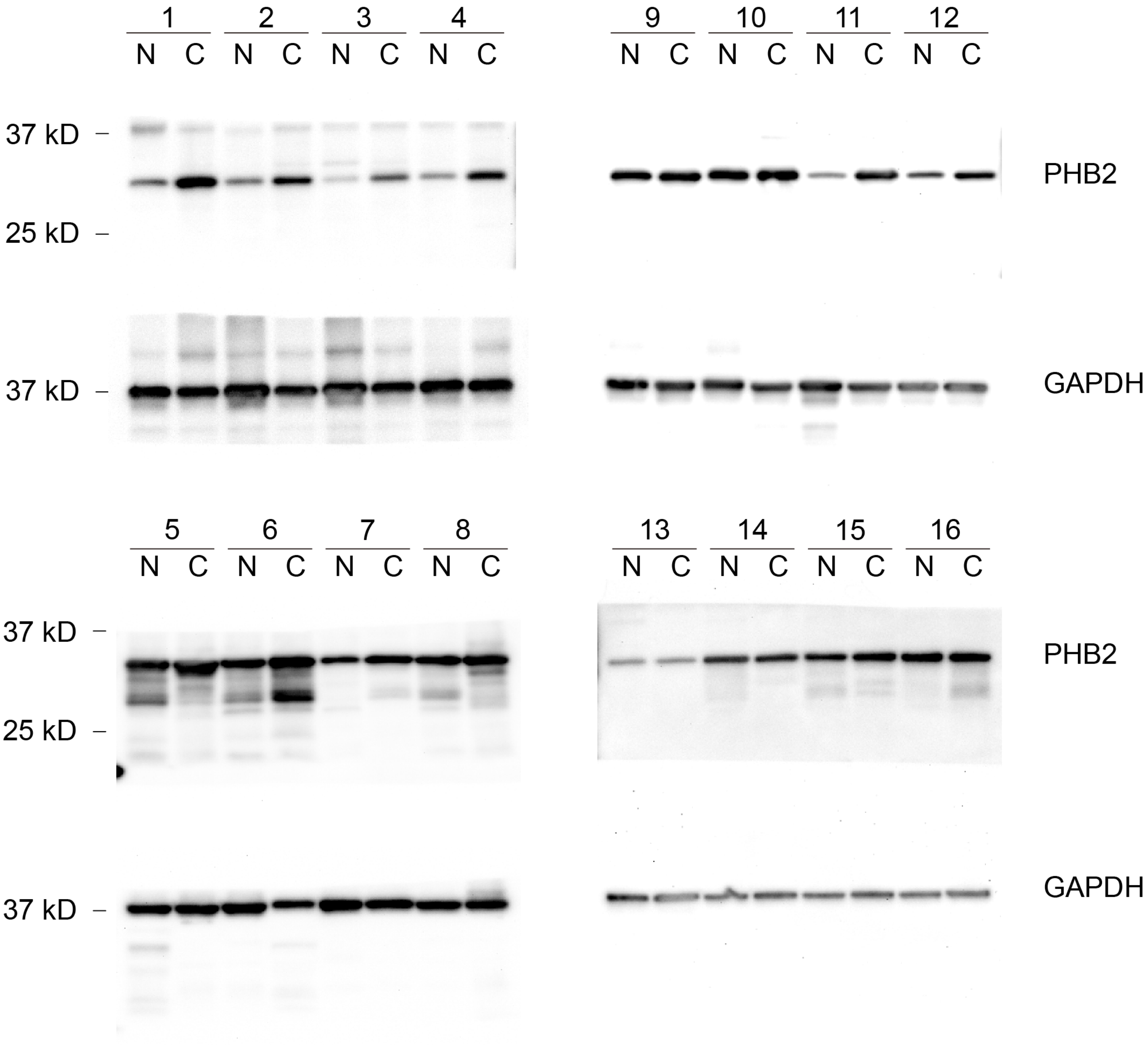

Original western blots for Fig 6E

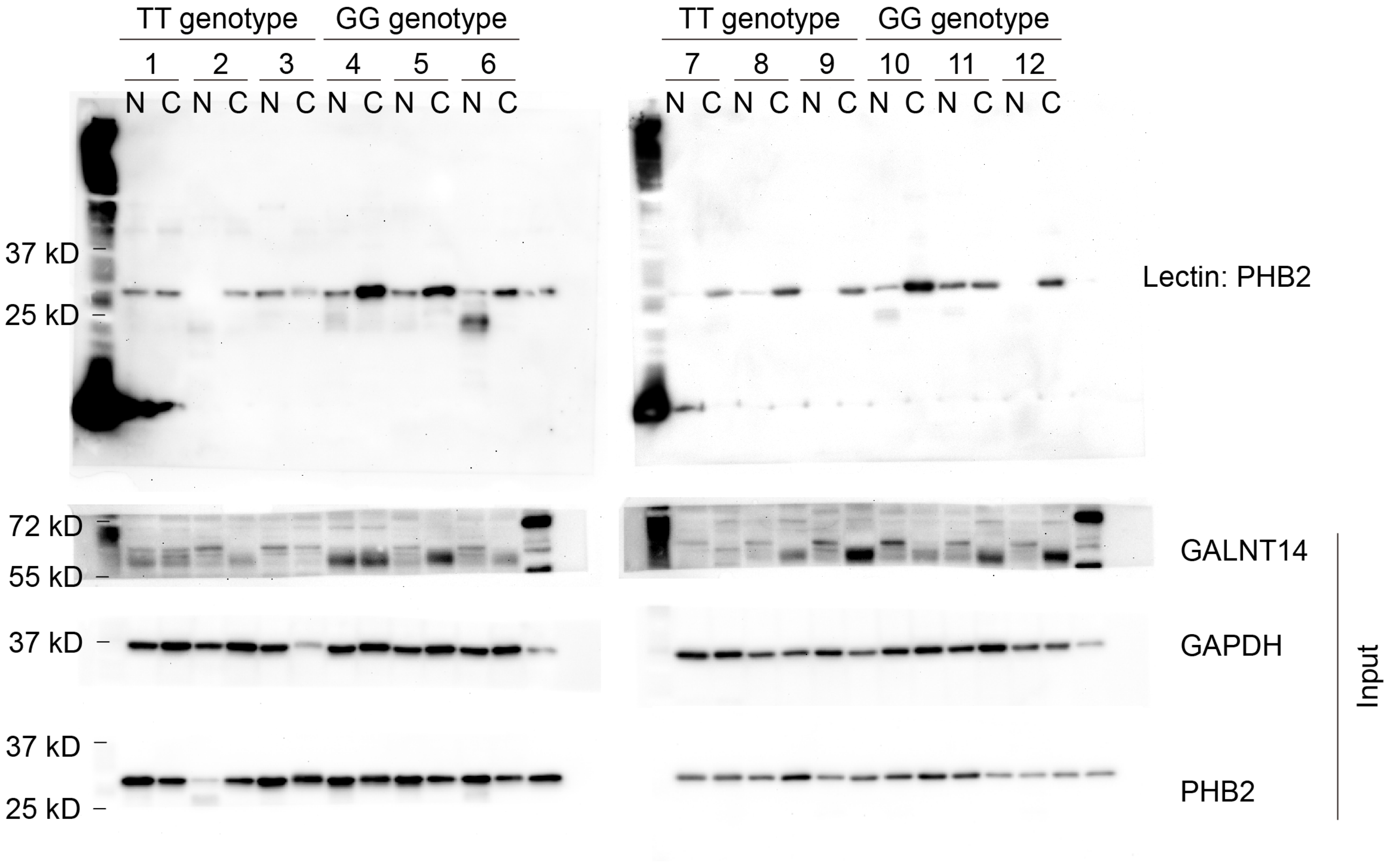

Original western blots for Fig 7A

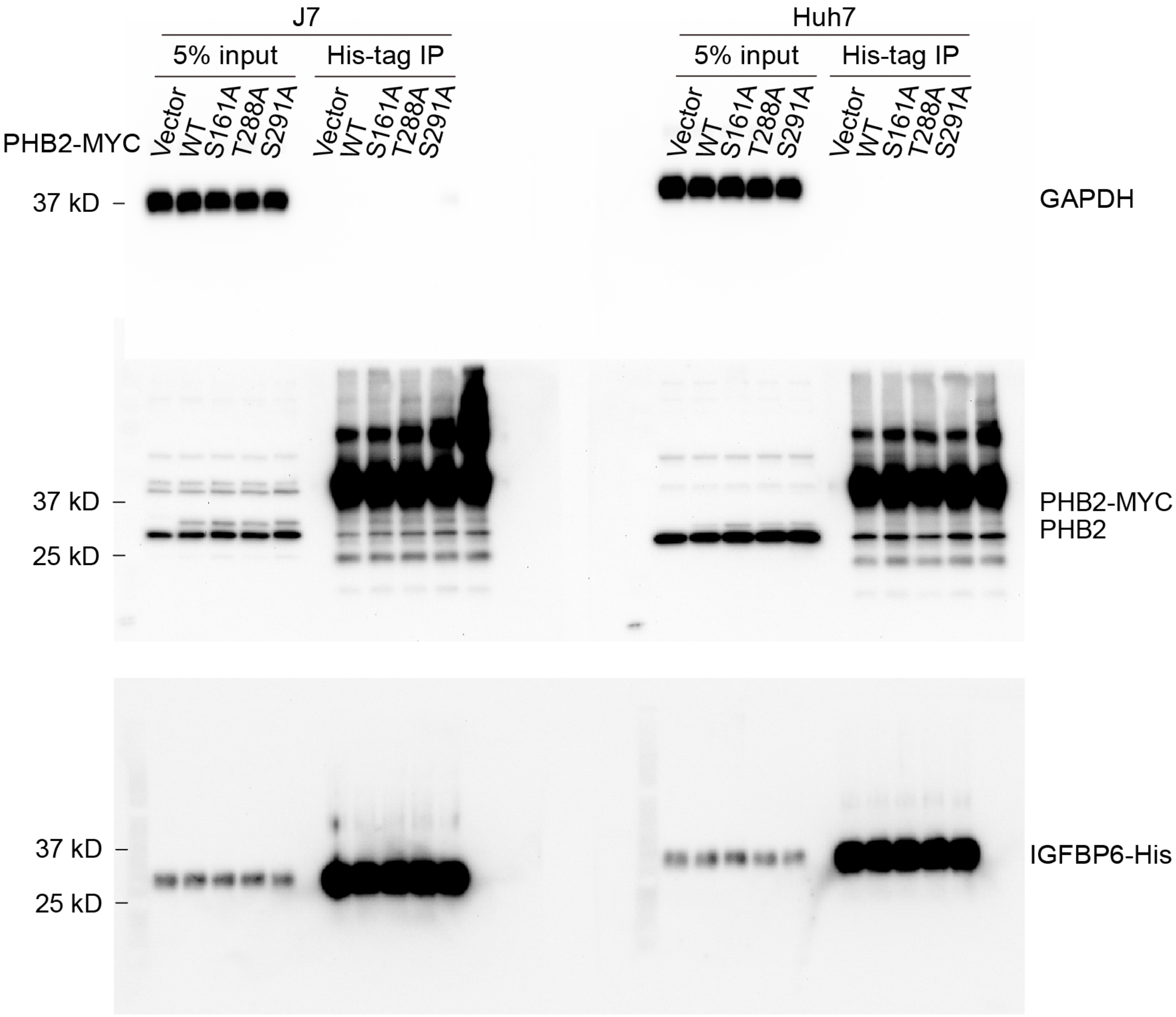

Original western blots for Fig 7B

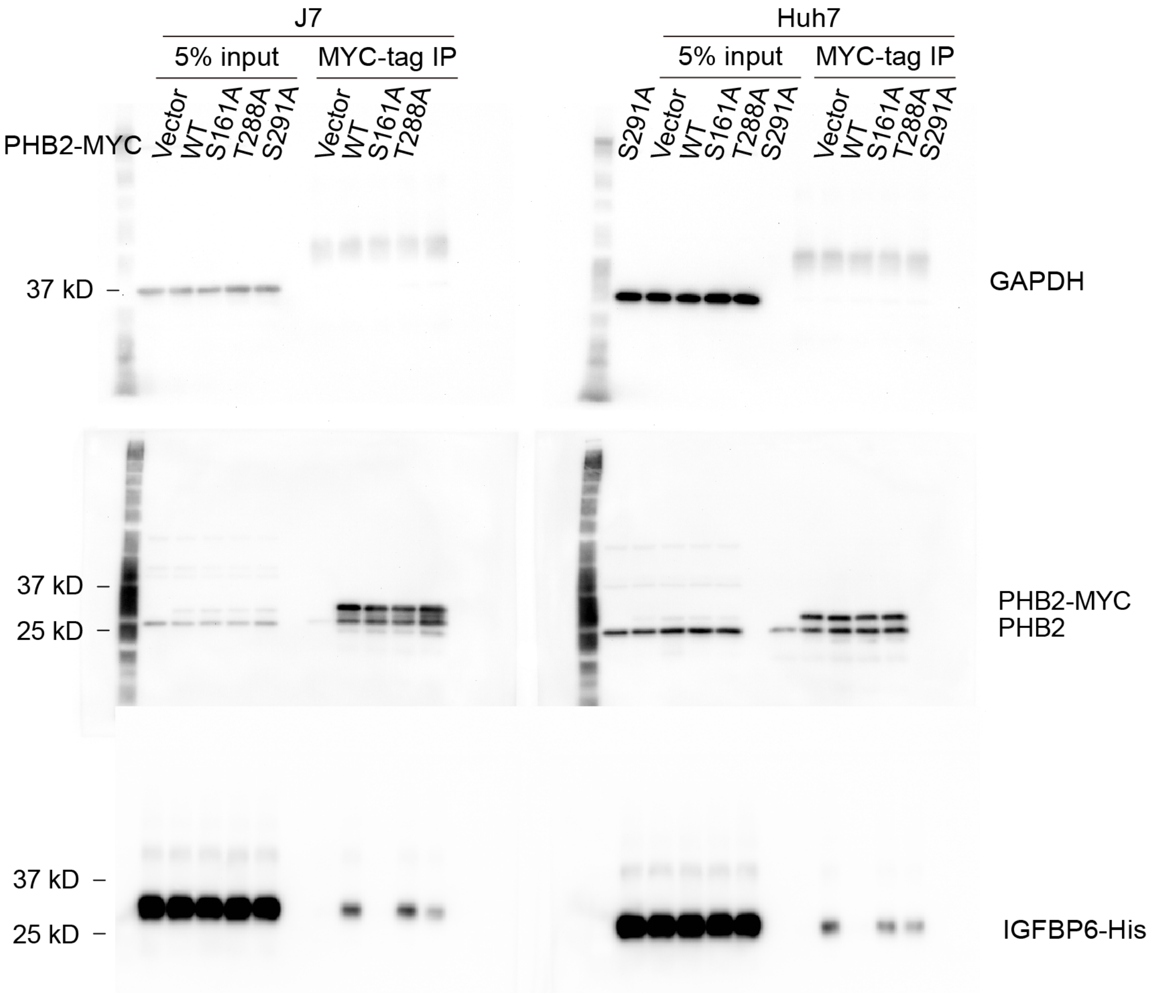

Original western blots for Fig 7C

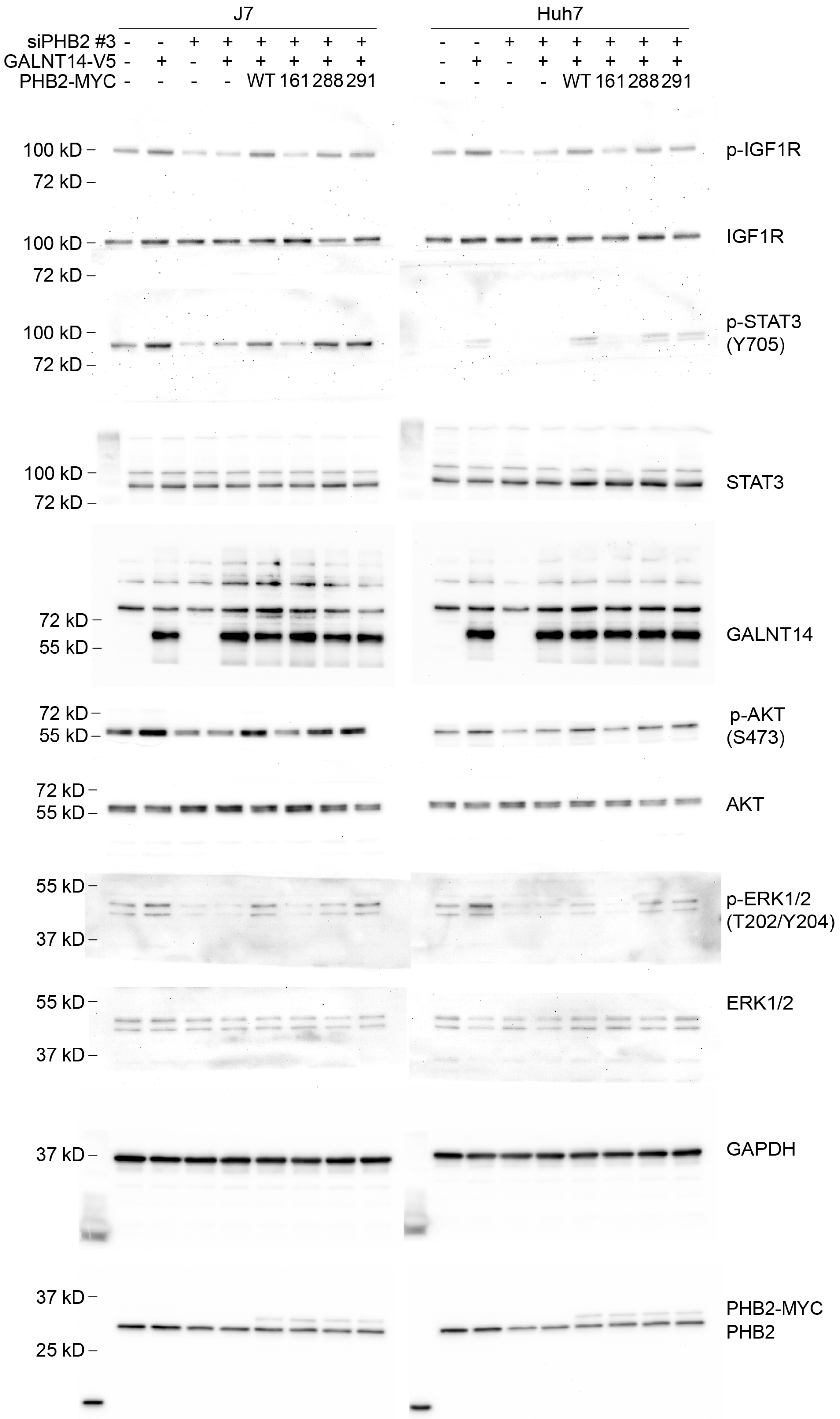

Supplement: Supplementary file 3 — Original Data File [file 41419_2022_5419_MOESM3_ESM.pdf]
